# A practical two-step procedure for the preparation of enantiopure pyridines: Multicomponent reactions of alkoxyallenes, nitriles and carboxylic acids followed by a cyclocondensation reaction

**DOI:** 10.3762/bjoc.7.108

**Published:** 2011-07-13

**Authors:** Christian Eidamshaus, Roopender Kumar, Mrinal K Bera, Hans-Ulrich Reissig

**Affiliations:** 1 Freie Universität Berlin, Institut für Chemie und Biochemie, Takustr. 3, D-14195 Berlin, Germany

**Keywords:** allenes, enantiopure pyridines, ketoenamides, multicomponent reactions, nonaflates

## Abstract

A practical approach to highly functionalized 4-hydroxypyridine derivatives with stereogenic side chains in the 2- and 6-positions is described. The presented two-step process utilizes a multicomponent reaction of alkoxyallenes, nitriles and carboxylic acids to provide β-methoxy-β-ketoenamides which are transformed into 4-hydroxypyridines in a subsequent cyclocondensation. The process shows broad substrate scope and leads to differentially substituted enantiopure pyridines in good to moderate yields. The preparation of diverse substituted lactic acid derived pyrid-4-yl nonaflates is described. Additional evidence for the postulated mechanism of the multicomponent reaction is presented.

## Introduction

The pyridine core is ubiquitous in pharmacologically active agents, agrochemicals and natural products [1–5]. The HMG-CoA reductase inhibitors Glenvastatin and Cerivastatin are exemplarily mentioned as pharmaceuticals that feature the pyridine nucleus [6–10]. Natural products that contain a pyridine ring include the 3-alkylpyridine alkaloid niphatesine C and the fuzanin family [11–12]. Moreover, the ability to form coordination compounds makes pyridines ideal ligands for transition metal-catalyzed processes and for the construction of supramolecular architectures [13]. Pyridines with chiral side chains are widely employed as ligands in asymmetric transformations, for instance, in the asymmetric hydrogenation of olefins, in enantioselective additions of metal organyls to aldehydes and enones, as well as in palladium-catalyzed allylic substitution reactions [14–20]. Thus, the synthesis of specifically functionalized pyridines is of considerable interest, and many approaches toward this heterocyclic structure have been disclosed in the literature [21]. In addition to classical pyridine syntheses such as the Kröhnke reaction, many new approaches have recently been developed [22–26]. Despite the wide range of conceptually different syntheses, only few methods for introducing chirality into pyridine side chains have been described: Enantioselective reduction of pyridine carbonyl compounds, the addition of lithiated pyridine derivatives to chiral ketones or the resolution of racemates being the most common approaches. The preparation of chiral pyridine derivatives starting from simple enantiopure precursors is less common [27–28].

**Scheme 1:**
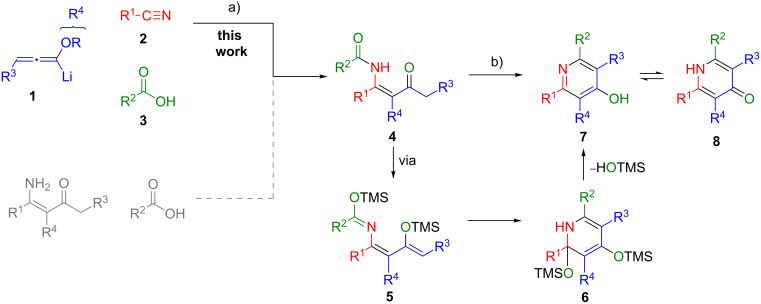
Preparation of β-ketoenamides and subsequent cyclocondensation to 4-hydroxypyridines. a) Et_2_O, −40 °C to r.t. 16 h, b) TMSOTf, NEt_3_, CH_2_Cl_2_ or ClCH_2_CH_2_Cl reflux.

Recently, we reported a new synthesis of pyridines based on the trimethylsilyl trifluoromethanesulfonate (TMSOTf) induced cyclocondensation reaction of β-ketoenamides [29–34]. This cyclocondensation step can be rationalized as a 6π-electrocyclization of the disilylated intermediate **5** to provide dihydropyridine **6**. Elimination of trimethylsilanol and subsequent *O*-desilylation affords the 4-hydroxypyridine **7** (Scheme 1). The desired β-ketoenamides **4** are either accessible by acylation of enaminoketones or by a multicomponent reaction of lithiated alkoxyallenes, nitriles and carboxylic acids [35–36].

In the past, we devoted considerable interest to the synthesis of substituted pyridine derivatives by this route and investigated their use in subsequent transformations [37–39]. Broadening the scope of the process to functionalized, chiral starting materials is the subject of this report. Additionally, herein we disclose the full experimental data for compounds reported in a preliminary communication [40].

## Results and Discussion

**Scheme 2:**
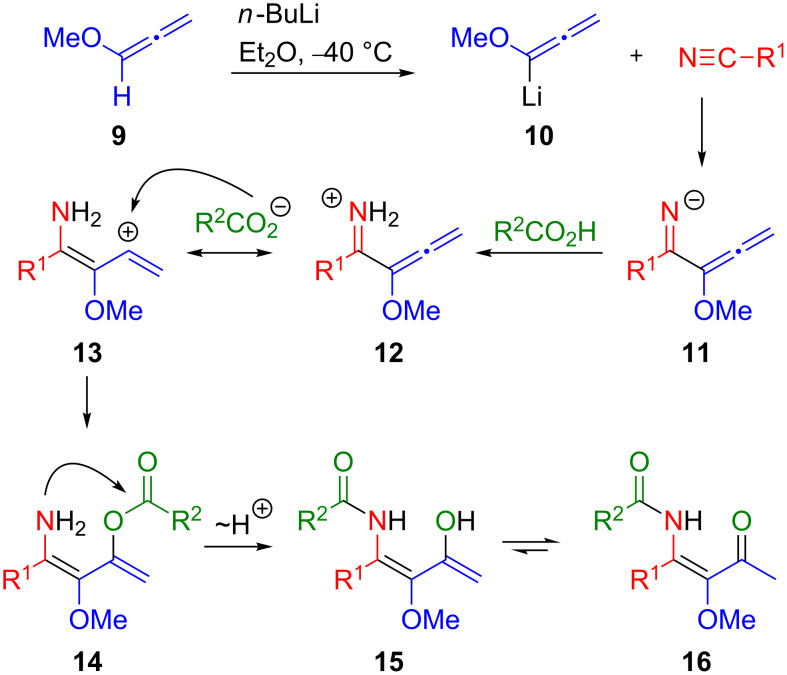
Mechanistic rational for the formation of β-ketoenamides **16**.

In continuation of our previous work, we addressed the question whether chiral starting materials react in the above sequence without loss of enantiopurity [40]. Chiral carboxylic acids are readily available and their use would allow for a rapid access to pyridines with side chains bearing stereogenic centers. In recent years we studied intensively the multicomponent reactions of lithiated alkoxyallenes with nitriles and carboxylic acids and could demonstrate that precursor compounds with alkyl, alkenyl or aryl substituents are smoothly converted into β-ketoenamides and subsequently transformed into the desired 4-hydroxypyridines. The mechanism of the multicomponent reaction is depicted in Scheme 2. In the first step, a lithiated alkoxyallene such as **10** adds to a nitrile to yield an iminoallenyl anion **11**. Protonation of **11** then gives a resonance stabilized cation **12** which can be attacked in β-position by a carboxylate to afford an enol ester **14**. The β-ketoenamide **16** is then formed by transfer of the acyl group to the amino group and subsequent tautomerization.

In some cases we observed the formation of minor amounts of 4-hydroxypyridines along with the β-ketoenamides. Depending on the substitution pattern of the β-ketoenamide, a condensation to the corresponding pyridine can spontaneously occur. In most cases a second step is necessary and the cyclocondensation must be induced or completed by treatment with TMSOTf and an amine base in a suitable solvents at elevated temperatures. In order to minimize the operational effort, the process can be performed as quasi-one-pot procedure without purification of the intermediary β-ketoenamide.

## Scope and limitations

Following the protocol mentioned before, we successfully prepared a series of 4-hydroxypyridines with chiral functional groups present in a side chain. As can be seen in Table 1 not merely chiral aliphatic carboxylic acids and nitriles such as **17** and **31** can be transformed into 4-hydroxypyridines, but rather complex substrates with appropriately protected functional groups. For instance, when lithiated methoxyallene was added to pivalonitrile and reacted with *O*-silylated mandelic acid **21**, pyridine derivative **22** was obtained in good yield over two steps. Furthermore, readily available *N*,*N*-dibenzylated amino acids, such as those derived from valine and phenylalanine, **23** and **25** gave the respective pyridines **24** and **26** in 45% and 50% yield, respectively. Carboxylic acids featuring aromatic units and branched side chains including quaternary α-carbon atoms were also tolerated. Besides chiral carboxylic acids, enantiopure nitriles were also successfully converted into pyridine derivatives in comparable yields. As an example, (*S*)-2-methylbutyronitrile (**31**) could be converted into compound **40** in good yield. The use of carboxylic acids and nitriles with structurally identical substituents allows a rapid access to pyridine derivatives such as **32** which almost has *C*_2_-symmetry. Compound **32** is derived from nitrile **31** and carboxylic acid **17** and was obtained in high yield after two steps. Products **34** and **35** were prepared from enantiopure acid **29** and racemic *O*-TBS-mandelonitrile **33**. The diastereomeric pyridines obtained from this reaction are easily separable by column chromatography to give **34** and **35** in moderate yields. If not commercially available, the desired nitriles were prepared from the corresponding acids by an amide formation/dehydration sequence according to literature procedures [41–42]. Not all transformations proceeded in very good yields, however, it should be noted that in only a few cases attempts to optimize the conditions have been undertaken. Hence, there may be room for improvement of yields in cases where the standard conditions led only to moderate yields.

**Table 1:** Scope of the synthesis of 4-hydroxypyridine derivatives from lithiated methoxyallene, nitriles and carboxylic acids.

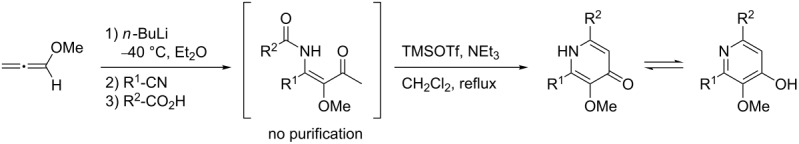			

Carboxylic Acid R^2^CO_2_H	Nitrile R^1^-CN	Product^a^	Yield^b^

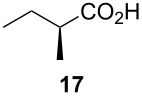	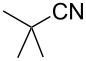	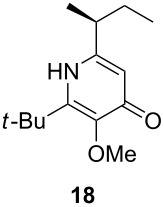	24%
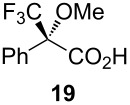	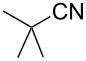	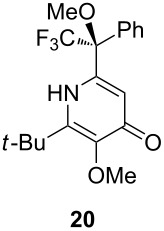	30%
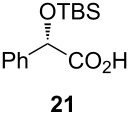	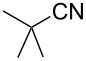	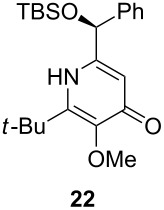	50%
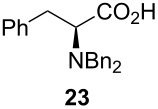	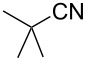	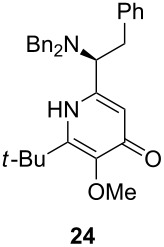	45%
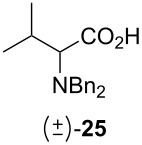	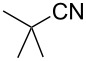	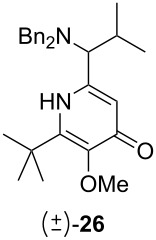	50%
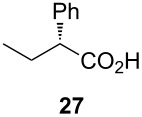	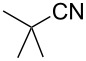	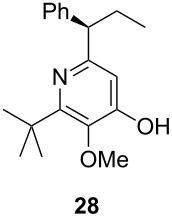	45%
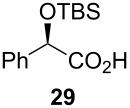	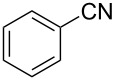	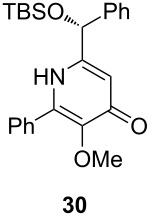	24%
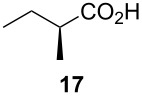	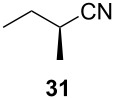	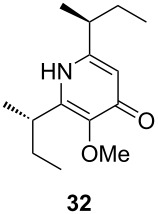	85%
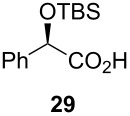	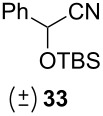	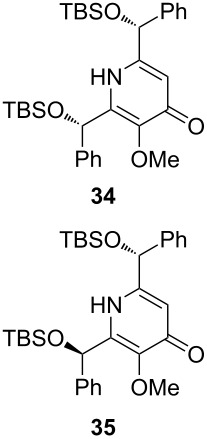	26% (as a separable 1:1 mixture of diastereomers)
CF_3_CO_2_H	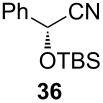	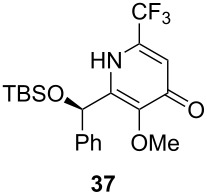	37%
CF_3_CO_2_H	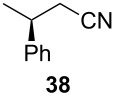	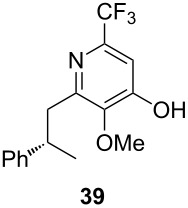	28%
CF_3_CO_2_H	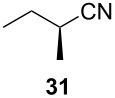	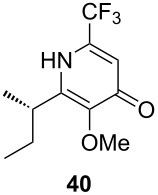	56%
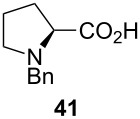	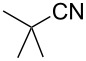	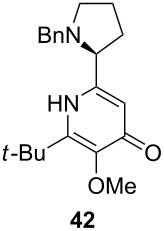	–
CF_3_CO_2_H	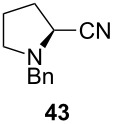	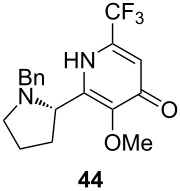	–

^a^Only the predominant tautomer in CDCl_3_ is depicted; ^b^All yields are based on the nitrile.

**Scheme 3:**
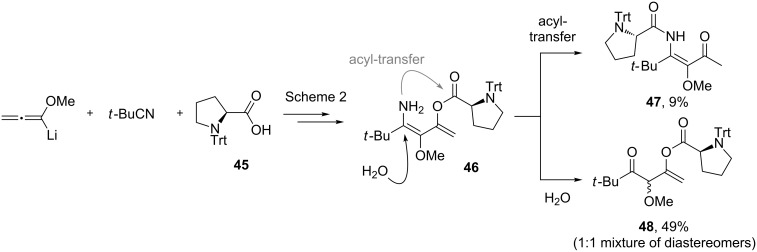
Reaction of proline derivative **45** and formation of β-ketoenamide **47** and enolester **48**.

Unfortunately, all attempts to incorporate proline-derived moieties failed. *N*-Benzylproline (**41**) turned out to be almost insoluble in ethereal solvents, which might explain why the desired β-ketoenamide was not formed [43]. To increase the solubility of the proline component, we changed the protective group from benzyl to the more lipophilic trityl group [44]. Surprisingly, the use of trityl-protected proline did not give the β-ketoenamide **47** as main product (Scheme 3). Instead, a diastereomeric 1:1 mixture of the β-keto-enolester **48** was isolated in 49% yield together with minor amounts of the expected product **47**. The formation of **48** is additional evidence for our previously suggested mechanism (Scheme 2). We assume that the bulkiness of the trityl group hampers the transfer of the acyl group from intermediate **46** to **47**. Upon the addition of water, the enamine moiety of **46** was hydrolyzed to furnish enol ester **48**.

The pyridines in Table 1 are depicted in their predominant tautomeric form as found in CDCl_3_ at ambient temperature. Interestingly, the pyridone/pyridinol equilibrium seems to depend on the substituents at the C-2 or C-6 side chains. In general, a hydrogen bond-acceptor seems to stabilize the pyridone tautomer, whereas the pyridinol tautomer is favored when a hydrogen bond-donor is present. Compound **37** exists exclusively as pyridone tautomer, but after desilylation the resulting product, with a free hydroxy group in the side chain, strongly prefers the pyridinol tautomer. It seems reasonable to assume that the pyridone tautomer is stabilized through an internal hydrogen bond between a silyl ether or a tertiary amine moiety of the side chain as observed for compounds **22** and **24**. Moreover, we found that the equilibrium is strongly influenced by the solvent. In CDCl_3_ pyridine **40** exclusively exists in its pyridone form, but in methanol-*d*_4_ the equilibrium shifts completely to the pyridinol tautomer.

## Subsequent transformations of the prepared pyridine derivatives

**Figure 1:**
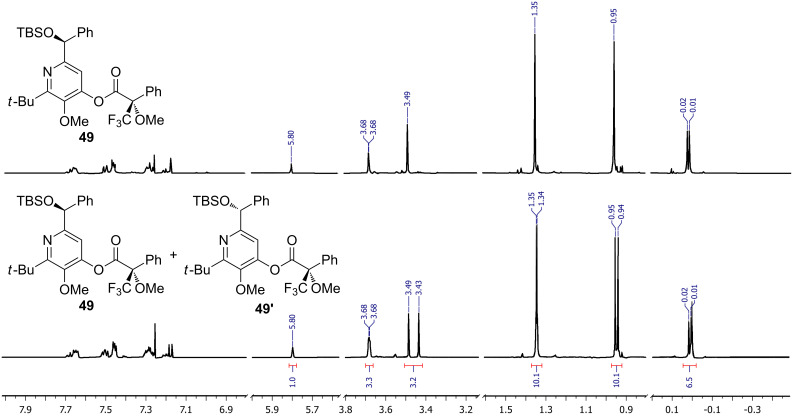
^1^H NMR spectra of **49** and the mixture of diastereoisomers **49** and **49’**.

To prove the enantiopurity of the pyridines derived from carboxylic acids and nitriles, which are prone to racemization, i.e., substrates with tertiary stereogenic centers in α-position, compounds **18**, **22** and **40** were transformed into esters **50**, **49** and **51**, respectively (Table 2). Treatment of the pyridones with Mosher acid chloride in a mixture of pyridine and dichloromethane as solvent afforded the desired esters in good yields. Comparison with the diastereomeric compounds obtained from racemic starting materials unambiguously shows that the sequence proceeds without noticeable racemization, since **49**, **50** and **51** were obtained in diastereomeric pure form (Table 2) as judged by ^1^H NMR analysis (estimated error 3–5%). For instance, the signal of the methoxy group at C-3 of diastereomeric **49’** (obtained by starting with racemic mandelic acid) appears at 3.43 ppm in the ^1^H NMR-spectrum, whereas this signal for **49** occurs at 3.49 ppm (Figure 1). In addition, the *tert*-butyl group of the OTBS groups of the two diastereoisomers **49** and **49’** show signals at different frequencies.

**Table 2:** Esterification of different pyridinol derivatives with the 3,3,3-trifluoro-2-methoxy-2-phenylpropanoic acid.

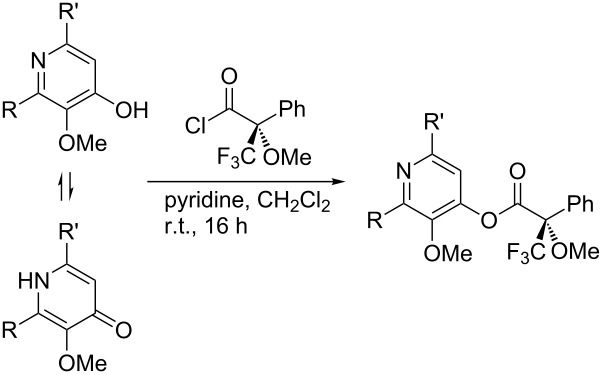		

Pyridine	Product	Yield

**22**	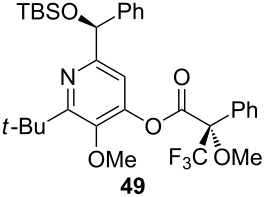	68% dr >95:5^a^
**18**	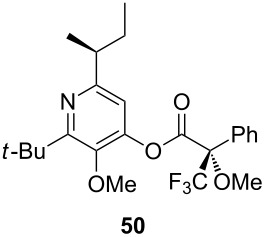	55% dr >95:5^a^
**40**	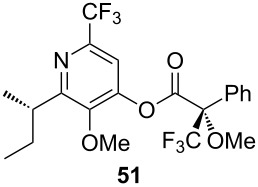	67% dr >95:5^a^

^a^Determined by ^1^H NMR spectroscopic analysis of the crude products.

**Scheme 4:**
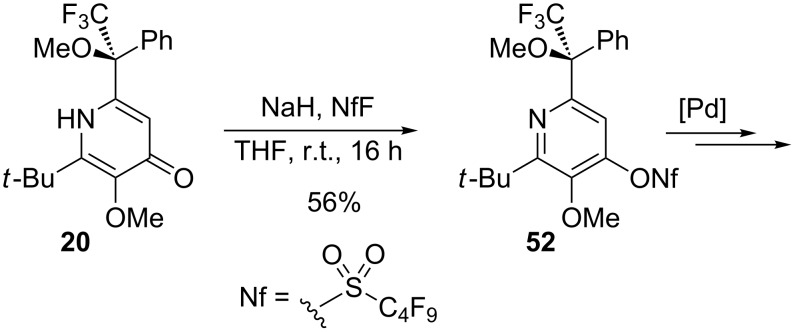
Synthesis of pyrid-4-yl nonaflate **52**.

To explore the chemistry of the synthesized pyridine derivatives, we investigated the selective functionalization of the 4-hydroxy group. Pyridone **20** was nonaflated according to previously established conditions to provide **52** in 56% yield (Scheme 4). As we have already demonstrated, pyrid-4-yl nonaflates are excellent coupling partners in palladium-catalyzed transformations such as Suzuki, Stille, Heck and Sonogashira reactions [45].

**Scheme 5:**
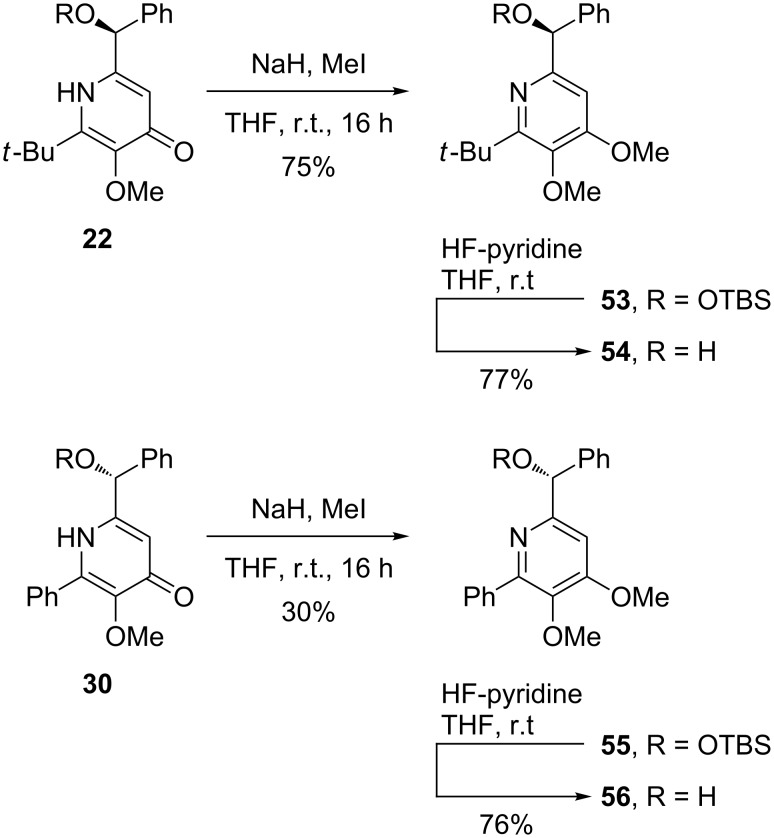
*O*-Methylation of pyridine derivatives **22** and **30** followed by desilylation.

However, in contrast to the smooth nonaflation, the selective *O*-alkylation of the synthesized pyridines turned out to be more challenging (Scheme 5). Whereas pyridone **22** could be *O*-methylated in good yield with methyl iodide in THF, the same conditions converted **30** into **55** in a disappointing 30% yield. Desilylation of **53** and **55** with HF in pyridine gave the desired deprotected pyridine derivatives **54** and **56** in high yields. This type of enantiopure hydroxymethyl-substituted pyridine derivatives is of particular interest as they are known to be efficient catalysts for the asymmetric addition of zinc organyls to aldehydes [14,46].

## Preparation of lactic acid derived pyrid-4-yl nonaflates

**Scheme 6:**
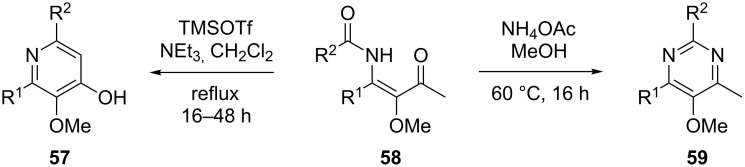
Formation of 5-alkoxypyrimidines from β-alkoxy-β-ketoenamides.

In the course of our investigations on the scope of the present procedure, we also discovered that easily available *O*-TBS-protected lactic acid and *O*-TBS-protected lactic nitrile are excellent reaction partners. We became interested in exploring the scope of this reaction with respect to lactic acid derived starting materials in more detail. In contrast to the previously described procedures, we decided to purify the reaction mixture at the stage of the β-alkoxy-β-ketoenamides **58** obtained by the three-component reaction. Recently we demonstrated that β-alkoxy-β-ketoenamides are not only valuable intermediates in the synthesis of 4-hydroxypyridines **57**, but that they can also serve as precursors in the synthesis of 5-alkoxypyrimidines **59** (Scheme 6). When β-alkoxy-β-ketoenamides **58** were treated with an ammonia source such as NH_4_OAc in MeOH, 5-alkoxypyrimidines with the general structure of **59** were formed in high yields [37–38,47]. By this simple change in the reaction conditions not only pyridine but also pyrimidine derivatives with lactic acid based side chains should be accessible.

*O*-TBS-protected lactic nitrile **63** was prepared following a literature procedure in four steps starting from enantiopure methyl lactate [48]. The scope of the multicomponent reaction with respect to lactic acid derived precursors is summarized in Table 3.

**Table 3:** Scope of the synthesis of β-alkoxy-β-ketoenamides derived from lactic acid based precursors.

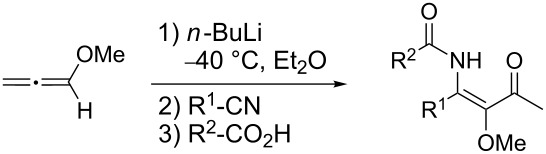			

Carboxylic Acid R^2^CO_2_H	Nitrile R^1^-CN	Product	Yield

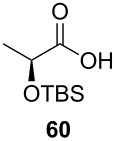	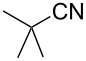	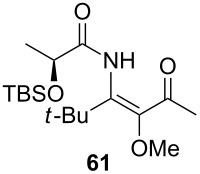	58%
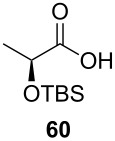	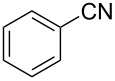	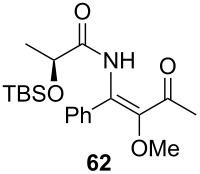	58%
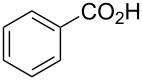	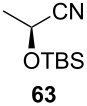	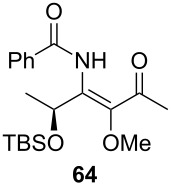	73%
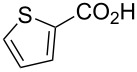	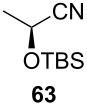	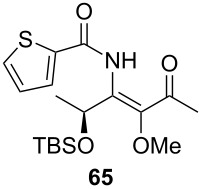	73%
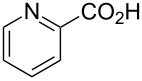	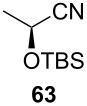	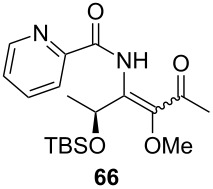	51%
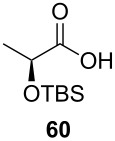	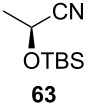	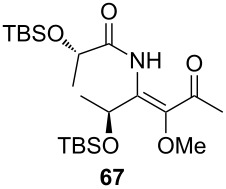	25%

Table 3 shows that *O*-TBS-protected lactic acid **60** and *O*-TBS-protected lactic nitrile **63** gave the desired ketoenamides in moderate to high yields. When lithiated methoxyallene was reacted with pivalonitrile or benzonitrile followed by the addition of *O*-TBS-protected lactic acid, the corresponding ketoenamides **61** and **62** were isolated in 58% yield. Reaction of lithiated methoxyallene with *O*-TBS-protected lactic nitrile and benzoic acid furnished **64** in high yield. Heterocyclic moieties were also well tolerated as demonstrated by the efficient reaction of 2-thiophene carboxylic acid. The relatively low yield in the formation of **66** might be explained by the poor solubility of 2-picolinic acid in ethereal solvents rather than for reactivity reasons. In contrast to the other examples, enamide **66** was obtained as a 1:1 mixture of (*E*)- and (*Z*)-isomers. This may be due to alternative hydrogen bond formation with the NH unit to the pyridine nitrogen rather than to the carbonyl group. Subsequent cyclocondensation with TMSOTf and NEt_3_ in 1,2-dichloroethane gave the expected pyridine derivatives, which were directly converted into pyrid-4-yl nonaflates in a second step. The results are depicted in Table 4.

**Table 4:** Cyclization and nonaflation of lactic acid derived β-alkoxy-β-ketoenamides.

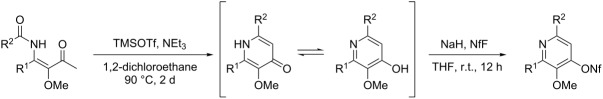		

β-Alkoxy-β-ketoenamide	Product	Yield^a^

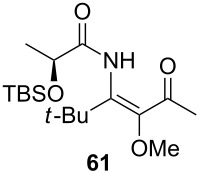	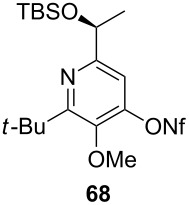	56%
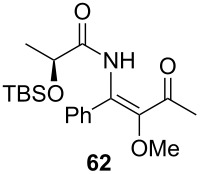	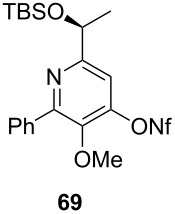	61%
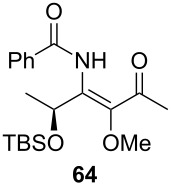	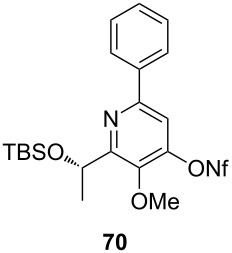	63%
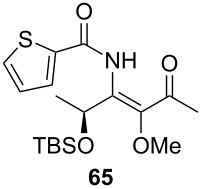	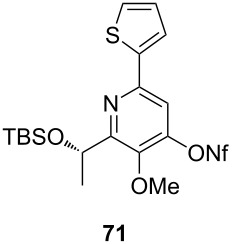	52%
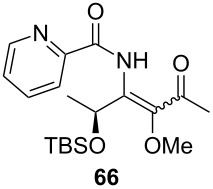	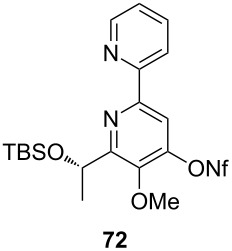	72%
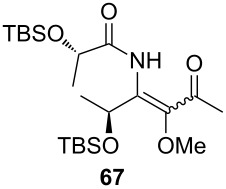	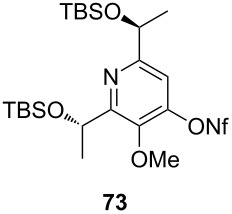	39%

^a^Yields over two steps based on the ketoenamide.

In all examples the cyclization/nonaflation sequence provided the pyrid-4-yl nonaflates in good yields. Apparently, the reactivity in this sequence is not strongly governed by the structure of the original ketoenamide. Even the configuration of the enamide double bond seems to have no influence on the cyclization, since the (*E*/*Z*)-mixture of enamide **66** also gave the corresponding pyridine in good yield. Obviously, the diastereomers are in equilibrium under the cyclization conditions. Of particular interest are the pyrid-4-yl nonaflates **71** and **72**, possessing a chiral side chain as well as heteroaromatic units. 2,2’-Bipyridines with structures similar to **72** might show interesting properties when used as ligands in asymmetric transformations. The nonaflate moiety should allow electronic fine tuning of the ligand properties in palladium-catalyzed or nucleophilic substitution reactions.

## Conclusion

We have demonstrated that enantiopure functionalized carboxylic acids and nitriles can be used without problems in our previously reported pyridine synthesis. The starting materials were successfully transformed into the corresponding pyridines without loss of enantiopurity to yield enantiopure 4-hydroxypyridine derivatives with stereogenic side chains at C-2 and C-6. The 4-hydroxy group allows further variations. Applications of the prepared pyridines as ligands or catalysts in asymmetric transformations will be studied and will be the subject of future reports.

## Experimental

**General methods:** Reactions were generally performed under an argon atmosphere in flame-dried flasks, and the components were added by syringe. Methanol was purchased in p.a. quality and stored under an argon atmosphere over molecular sieves (4 Å). Triethylamine was distilled from CaH_2_ and stored over KOH under an atmosphere of argon. Pyridine was used as purchased and stored over KOH under an atmosphere of argon. 1,2-Dichloroethane was purchased in p.a. quality and stored over molecular sieves (4 Å) under an atmosphere of argon. Tetrahydrofuran, diethyl ether, toluene and dichloromethane were obtained from the solvent purification system MB-SPS-800 (M. Braun). Products were purified by flash chromatography on silica gel (230–400 mesh, Merck). Unless otherwise stated, yields refer to analytically pure samples. Internal standards: for ^1^H NMR CDCl_3_ (δ = 7.26 ppm), TMS (δ = 0.00 ppm), CD_3_OD (δ = 3.31 ppm), C_6_D_6_ (δ = 7.16 ppm), for ^13^C NMR CDCl_3_ (δ = 77.0 ppm), CD_3_OD (δ = 49.0 ppm), C_6_D_6_ (δ = 128.1 ppm). NMR spectra were recorded on Bruker AC 250, ECP 400, AC 500, AVIII 700, or Jeol Eclipse 500 instruments in CDCl_3_, CD_3_OD, or C_6_D_6_ solution. Integrals are in accord with assignments; coupling constants are given in Hz. IR spectra were measured with a FT-IR spectrometer Nicolet 5 SXC or with a Nexus FT-IR equipped with a Nicolet Smart DuraSamplIR ATR. MS and HRMS analyses were obtained with Finnigan Varian Ionspec QFT-7 (ESI-FT-ICR) and Agilent ESI-TOF 6210 (4 μL/min, 1 bar, 4000 V) instruments. Elemental analyses were obtained with “Elemental-Analyzers“ (Perkin–Elmer or Carlo Erba). Melting points were measured with a Reichert apparatus (Thermovar) and are uncorrected. Optical rotations ([α]_D_) were determined with a Perkin–Elmer 241 polarimeter at the temperatures given. Commercially available chemicals were used without further purification unless otherwise stated.

### Typical procedure for the preparation of 3-methoxy-4-hydroxypyridines without isolation of the intermediate β-alkoxy-β-ketoenamide (Procedure 1)

A solution of *n*-BuLi (2.5 M in hexanes, 0.31 mL, 0.79 mmol) was added dropwise to a solution of methoxyallene (59 µL, 0.71 mmol) in diethyl ether (5 mL) at −40 °C. After stirring at that temperature for 15 min, pivalonitrile was added (59 mg, 0.71 mmol) and the resulting yellow solution stirred for 4 h at −40 °C. The solution was then cooled to −78 °C and (*S*)-2-methylbutyric acid (0.23 mL, 2.14 mmol) added. Stirring was continued overnight during which time the mixture was slowly allowed to reach r.t. The reaction was quenched by the addition of sat. aq. NaHCO_3_ solution (10 mL) and the aqueous phase extracted with diethyl ether (2 × 20 mL). The combined organic layers were washed with brine, dried with Na_2_SO_4_, filtered and the solvent was evaporated under reduced pressure. The residue was re-dissolved in CH_2_Cl_2_ (14 mL) and TMSOTf (0.41 mL, 2.1 mmol) and NEt_3_ (0.30 mL, 2.1 mmol) were added. The mixture was heated under reflux under an atmosphere of argon for 2 d. After complete consumption of the starting material (by TLC), the reaction was quenched by the addition of aq. sat. NH_4_Cl solution (20 mL) and the aqueous layer extracted with CH_2_Cl_2_ (2 × 30 mL). The combined organic layers were dried with Na_2_SO_4_, filtered and evaporated. The residue was purified by flash column chromatography on silica gel (eluent: hexane/ethyl acetate 1:9) to afford **18** (41 mg, 24%) as colorless crystals.

**(*S*)-6-*sec*-Butyl-2-*tert*-butyl-3-methoxypyridin-4-one (18):** mp 109–110 °C; [α]_D_^22^ +20.9 (*c* 2.3, CHCl_3_); ^1^H NMR (500 MHz, CDCl_3_) δ 0.90 (t, *J* ≈ 7 Hz, 3H, 4’-H), 1.24 (d, *J =* 6.9 Hz, 3H, 1’-H), 1.43 (s, 9H, *t*-Bu), 1.59 (quint, *J* ≈ 7 Hz, 2H, 3’-H), 2.47 (sext, *J ≈* 7 Hz, 1H, 2’-H), 3.94 (s, 3H, OMe), 6.26 (s, 1H, 5-H), 7.74 (s, 1H, NH) ppm; ^13^C NMR (101 MHz, CDCl_3_) δ 11.8 (q, C-4’), 19.5 (q, C-1’), 28.4, 29.5 (2, s, *t*-Bu), 35.1 (t, C-3’), 39.9 (d, C-2’), 58.9 (q, OMe), 114.2 (d, C-5), 146.0, 146.4, 150.7 (3 s, C-2, C-3, C-6), 176.1 (s, C-4) ppm; IR (KBr) 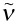: 3250 (N-H), 2965–2910 (=C-H, C-H), 1620 (C=O), 1580, 1540 (C=C) cm^−1^; HRMS–ESI (*m*/*z*): [M + H]^+^ calcd for C_14_H_24_NO_2_, 238.1807; found, 238.1803; Anal. calcd for C_14_H_23_NO_2_: C, 70.85; H, 9.77; N, 5.90; found: C, 70.81; H, 9.79; N, 5.38.

### Typical procedure for the preparation of β-alkoxy-β-ketoenamides (Procedure 2)

A solution of *n*-BuLi (1.30 mL, 3.28 mmol, 2.5 M in hexanes) was added to a solution of methoxyallene (0.30 mL, 3.28 mmol) in diethyl ether (20 mL) at −50 °C. After stirring for 30 min at −50 °C, the reaction mixture was cooled to −78 °C and (*S*)-TBS-lactic nitrile (200 mg, 1.14 mmol) in anhydrous diethyl ether (5 mL) was added to the mixture. After stirring for 4 h, a solution of benzoic acid (0.84 g, 6.88 mmol) in anhydrous DMF (10 mL) was added. The mixture was stirred overnight and slowly allowed to reach r.t. The reaction was quenched with sat. aq. NaHCO_3_ solution (15 mL) and the product extracted with diethyl ether (3 × 40 mL). The combined organic layers were washed with brine, dried with Na_2_SO_4_, filtered and the solvent was removed under reduced pressure. The crude product was purified by column chromatography on silica gel (eluent:hexane/EtOAc 3:1) to give **64** as a pale yellow oil (300 mg, 73%).

**(*S*)-*N*-{1-[1-(*tert*-Butyldimethylsiloxy)ethyl]-2-methoxy-3-oxo-but-1-enyl}benzamide (64) **^1^H NMR (500 MHz, CDCl_3_) δ 0.07, 0.11, 0.88 (3 s, 3H, 3H, 9H, OTBS), 1.46 (d, *J* = 6.4 Hz, 3H, 2’’-H), 2.32 (s, 3H, 4’-H), 3.51 (s, 3H, OMe), 5.33 (q, *J* = 6.4 Hz, 1H, 1’’-H), 7.43–7.53, 7.85–7.87 (2 m, 3H, 2H, Ph), 10.10 (br s, 1H, NH) ppm; ^13^C NMR (125 MHz, CDCl_3_) δ −4.8, −4.7 (2 q, OTBS), 18.2 (q, C-4’), 21.9 (q, C-2’’), 25.9, 27.2 (2, q, OTBS), 60.9 (q, OMe), 65.6 (d, C-1’’), 127.5, 128.8, 132.2, 134.2, 139.6, 141.9 (3 d, 3 s, Ph, C-1’, C-2’), 165.0 (s, C-1), 200.2 (s, C-3’) ppm; IR (ATR) 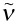: 3315 (NH), 2955–2855 (=CH, C-H), 1720–1515 (C=O, C=C) cm^−1^; HRMS–ESI (*m*/*z*): [M + H]^+^ calcd for C_20_H_31_NNaO_4_Si, 400.1915; found, 400.1930.

### Typical procedure for the cyclization of β-alkoxy-β-ketoenamides to 4-hydroxypyridines and subsequent nonaflation (Procedure 3)

Enamide **64** (40 mg, 0.11 mmol) was dissolved in 1,2-dichloroethane (2 mL) and placed in a sealable tube. Triethylamine (48 µL, 0.32 mmol) and TMSOTf (58 µL, 0.32 mmol) were added at r.t., and the resulting mixture was heated at 90 °C for 2 d. After complete consumption of the starting material (TLC), the reaction was quenched with sat. aq. NH_4_Cl solution (2 mL). After extraction with dichloromethane (3 × 10 mL), the combined organic layers were dried with Na_2_SO_4_ filtered and the solvent was removed under reduced pressure. The crude product was purified by flash column chromatography (SiO_2_, EtOAc/Methanol 10:1) to afford the respective pyridine derivative (36 mg, 94%) as a brown liquid.

The pyridine derivative (33 mg, 0.09 mmol) was dissolved in THF (3 mL) and NaH (6.6 mg, 0.28 mmol) added under an argon atmosphere. Nonafluorobutanesulfonyl fluoride (50 µL, 0.28 mmol) was added dropwise at room temperature. The mixture was stirred at the same temperature for 12 h and quenched by the slow addition of water. The resulting product was extracted with diethyl ether (3 × 10 mL), dried with Na_2_SO_4_, filtered and concentrated to dryness. The residue was purified by column chromatography on silica gel (eluent: 2–5% EtOAc in hexane) to afford **70** (39 mg, 67%) as a colorless oil.

**(*S*)-2-[1-(*tert*-Butyldimethylsiloxy)ethyl]-3-methoxy-6-phenyl-pyridin-4-yl nonaflate (70):** [α]_D_^22^ −21.2 (*c* 0.3, CHCl_3_); ^1^H NMR (500 MHz, CDCl_3_) δ 0.00, 0.05, 0.87 (3 s, 3H, 3H, 9H, OTBS), 1.60 (d, *J* = 6.6 Hz, 3H, 2’-H), 3.95 (s, 3H, OMe), 5.30 (q, *J* = 6.6 Hz, 1H, 1’-H), 7.42–7.48 (m, 3H, Ph), 7.51 (s, 1H, 5-H), 7.97–7.98 (m, 2H, Ph) ppm; ^13^C NMR (125 MHz, CDCl_3_) δ −4.6, −4.4 (2 q, OTBS), 18.3 (q, C-2’), 25.9, 30.3 (2, s, OTBS), 62.6 (q, OMe), 68.8 (d, C-1’), 112.3, 126.9, 128.8, 129.5, 137.7, 144.4, 150.1, 153.7, 160.0 (4 d, 5 s, Ph, C-2, C-3, C-4, C-5, C-6) ppm; IR (ATR) 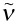: 3310 (NH), 3010–2835 (=CH, C-H), 1685–1510 (C=O, C=C) cm^−1^; HRMS–ESI (*m*/*z*): [M + H]^+^ calcd for C_24_H_29_F_9_NO_5_SSi, 642.1387; found, 642.1403.

### Typical procedure for the esterification of 4-pyridones with 3,3,3-trifluoro-2-methoxy-2-phenylpropanoic acid (Procedure 4)

Pyridone **22** (22 mg, 0.06 mmol) was dissolved in anhydrous CH_2_Cl_2_ (0.3 mL) and anhydrous pyridine (0.3 mL), and (*S*)-3,3,3-trifluoro-2-methoxy-2-phenylpropanoic acid (17 µL, 0.09 mmol) added. The mixture was stirred under an atmosphere of argon at r.t. for 16 h. After complete consumption of the starting material (TLC), the mixture was diluted with CH_2_Cl_2_ (10 mL) and the organic layer was successively washed with sat. NaHCO_3_ solution, 1 M HCl and H_2_O (10 mL each). The organic layer was dried with Na_2_SO_4_, filtered and the solvent was removed under reduced pressure to afford **49** (25 mg, 68%) as a colorless oil.

**(*S,S)*-2-*tert*-Butyl-6-[(*tert*-butyldimethylsiloxy)phenylmethyl]-3-methoxypyridin-4-yl 3,3,3-trifluoro-2-methoxy-2-phenylpropanoate (49):** [α]_D_^22^ +14.0 (*c* 1.0, CHCl_3_); ^1^H NMR (500 MHz, CDCl_3_) δ 0.01, 0.02, 0.96 (s, 3H, 3H, 9H, OTBS), 1.35 (s, 9H, *t*-Bu), 3.49, 3.68 (2 s, 3H each, OMe), 5.80 (s, 1H, 1’-H), 7.17 (s, 1H, 5-H), 7.19–7.22, 7.27–7.31, 7.48–7.53, 7.61–7.71 (4 m, 10H, Ph) ppm; ^19^F NMR (376 MHz, CDCl_3_) δ −71.1 (s, CF_3_) ppm; IR (neat) 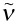: 2955–2930 (C-H), 1775 (C=O), 1570–1450 (C=C), 1170–1105 (=C-H), 780–700 (C-F) cm^−1^; HRMS–ESI (*m*/*z*): [M + H]^+^ calcd for C_33_H_43_F_3_NO_5_Si, 618.2857; found, 618.2896.

## Supporting Information

File 1 Experimental procedures and characterization data.

File 2 ^1^H NMR and ^13^C NMR spectra of synthesized compounds.

## References

[1] Newkome G R, Newkome G R (1984). Chemistry of Heterocyclic Compounds.

[2] McKillop A, Boulton A J, Katritzky A R, Rees C W (1984). Comprehensive Heterocyclic Chemistry.

[3] Kleemann A, Engel J, Kutscher B (2000). Pharmaceutical Substances.

[4] Spitzner D (2004). Science of Synthesis.

[5] Jones G, McKillop A (1996). Comprehensive Heterocyclic Chemistry II.

[6] Wess G, Kesseler K, Baader E, Bartmann W, Beck G, Bergmann A, Jendralla H, Bock K, Holzstein O, Kleine H (1990). Tetrahedron Lett.

[7] Miyachi N, Yanagawa Y, Iwasaki H, Ohara Y, Hiyama T (1993). Tetrahedron Lett.

[8] Beck G (2002). Synlett.

[9] Beck G, Kesseler K, Baader E, Bartmann W, Bergmann A, Granzer E, Jendralla H, Von Kerekjarto B, Krause R (1990). J Med Chem.

[10] Cattaneo D, Baldelli S, Merlini S, Zenoni S, Perico N, Remuzzi G (2004). Expert Opin Ther Pat.

[11] O'Hagan D (1997). Nat Prod Rep.

[12] Aida W, Ohtsuki T, Li X, Ishibashi M (2009). Tetrahedron.

[13] Lehn J-M (1995). Supramolecular Chemistry - Concepts and Perspectives.

[14] Bolm C, Schlingloff G, Harms K (1992). Chem Ber.

[15] Kwong H-L, Yeung H-L, Yeung C-T, Lee W-S, Lee C-S, Wong W-L (2007). Coord Chem Rev.

[16] Bolm C, Ewald M, Felder M (1992). Chem Ber.

[17] Bolm C, Ewald M, Zehnder M, Neuburger M A (1992). Chem Ber.

[18] Trost B M, Weiss A H (2009). Adv Synth Catal.

[19] Drury W J, Zimmermann N, Keenan M, Hayashi M, Kaiser S, Goddard R, Pfaltz A (2003). Angew Chem, Int Ed.

[20] Roseblade S J, Pfaltz A (2007). Acc Chem Res.

[21] Henry G D (2004). Tetrahedron.

[22] Movassaghi M, Hill M D, Ahmad O K (2007). J Am Chem Soc.

[23] Liu S, Liebeskind L S (2008). J Am Chem Soc.

[24] Manning J R, Davies H M L (2008). J Am Chem Soc.

[25] Xin X, Wang Y, Kumar S, Liu X, Lin Y, Dong D (2010). Org Biomol Chem.

[26] Hill M D (2010). Chem–Eur J.

[27] Rahm F, Stranne R, Bremberg U, Nordstrom K, Cernerud M, Macedo E, Moberg C (2000). J Chem Soc, Perkin Trans 1.

[28] Rahm F, Fischer A, Moberg C (2003). Eur J Org Chem.

[29] Flögel O, Dash J, Brüdgam I, Hartl H, Reissig H-U (2004). Chem–Eur J.

[30] Dash J, Lechel T, Reissig H-U (2007). Org Lett.

[31] Lechel T, Dash J, Hommes P, Lentz D, Reissig H-U (2009). J Org Chem.

[32] Lechel T, Reissig H-U (2010). Pure Appl Chem.

[33] Brasholz M, Reissig H-U, Zimmer R (2009). Acc Chem Res.

[34] Lechel T, Dash J, Brüdgam I, Reissig H-U (2008). Eur J Org Chem.

[35] Zimmer R, Reissig H-U, Krause N, Hashmi A S K (2004). Donor-Substituted Allenes. Modern Allene Chemistry.

[36] Reissig H-U, Zimmer R, Krause N (2007). Cumulenes and Allenes: Synthesis from Other Allenes. Science of Synthesis.

[37] Bera M K, Reissig H-U (2010). Synthesis.

[38] Zimmer R, Lechel T, Rancan G, Bera M K, Reissig H-U (2010). Synlett.

[39] Lechel T, Dash J, Eidamshaus C, Brüdgam I, Lentz D, Reissig H-U (2010). Org Biomol Chem.

[40] Eidamshaus C, Reissig H-U (2009). Adv Synth Catal.

[41] Couty F, David O, Larmanjat B, Marrot J (2007). J Org Chem.

[42] Aureggi V, Frankevicius V, Kitching M O, Ley S V, Longbottom D A, Oelke A J, Sedelmeier G (2008). Org Synth.

[43] Traverse J F, Zhao Y, Hoveyda A H, Snapper M L (2005). Org Lett.

[44] Barlos K, Papaioannou D, Theodoropoulos D (1982). J Org Chem.

[45] Högermeier J, Reissig H-U (2009). Adv Synth Catal.

[46] Liebehentschel S, Cvengroš J, Jacobi von Wangelin A (2007). Synlett.

[47] Lechel T, Möhl S, Reissig H-U (2009). Synlett.

[48] Massad S K, Hawkins L D, Baker D C (1983). J Org Chem.

