# Supplementary material for: A practical two-step procedure for the preparation of enantiopure pyridines: Multicomponent reactions of alkoxyallenes, nitriles and carboxylic acids followed by a cyclocondensation reaction
Source: Beilstein J Org Chem. 2011 Jul 13;7:962–75. doi: 10.3762/bjoc.7.108 (PMC3170199; doi:10.3762/bjoc.7.108)
Supplement: File 1 — Experimental procedures and characterization data. [file Beilstein_J_Org_Chem-07-962-s001.pdf]

Supporting Information

for

A practical two-step procedure for the preparation of enantiopure pyridines: Multicomponent reactions of alkoxyallenes, nitriles and carboxylic acids followed by a cyclocondensation reaction

Christian Eidamshaus, Roopender Kumar, Mrinal K. Bera and Hans-Ulrich Reissig*

Address: Freie Universität Berlin, Institut für Chemie und Biochemie, Takustr. 3, D-14195 Berlin, Germany

Email: Hans-Ulrich Reissig* - hreissig@chemie.fu-berlin.de

* Corresponding author

Experimental procedures and characterization data

Compounds **21**[1], **23**[2], **25**[3], **41**[4] and **43**[5] were prepared following literature procedures.

(*S*)-3-Phenylbutanamide

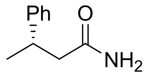

Boc_2O (558 mg, 2.55 mmol), NH_4HCO_3 (186 mg, 2.36 mmol) and pyridine (96 µL, 1.18 mmol) were added to a solution of (*S*)-3-phenylbutyric acid (323 mg, 1.97 mmol) in anhydrous CH_3CN (10 mL). The resulting mixture was stirred at r.t. under an atmosphere of argon for 16 h. After complete consumption of the starting material (as indicated by TLC) all the volatile components were removed under reduced pressure. The residue was dissolved in EtOAc and water added. The phases were separated and the aqueous layer was extracted with EtOAc. The combined organic layers were washed with brine, dried with Na_2SO_4, filtered, evaporated and the product used without further purification.

(*S*)-3-Phenylbutanenitrile (38)

---

[1] Kuisle, O.; Quinoá, E.; Riguera, R. *J. Org. Chem.* **1999,** *64,* 8063–8075. doi:10.1021/jo981580+

[2] Reetz, M. T.; Drewes, M. W.; Lennick, K. Process for the stereoselective preparation of optically active S,S or R,R-beta-aminoalcohols. Eur. Pat. Appl. EP 387605, September 9, 1990.

[3] Velluz, L.; Amiard, G.; Heymes, R. *Bull. Soc. Chim. Fr.* **1955,** 201–204.

[4] Traverse, J. F.; Zhao, Y.; Hoveyda, A. H.; Snapper, M. L. *Org. Lett.* **2005,** *7,* 3151–3154. doi:10.1021/ol050814q

[5] Couty, F.; David, O.; Larmanjat, B.; Marrot, J. *J. Org. Chem.* **2007,** *72,* 1058–1061. doi:10.1021/jo062221e

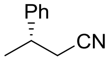

Cyanuric choride (56 mg, 0.30 mmol) was added to a solution of (*S*)-3-phenylbutanamide (75 mg, 0.46 mmol) in DMF (2.5 mL) at 0 °C. The mixture was slowly allowed to reach r.t. and stirring at that temperature continued overnight. The reaction was quenched by the addition of water and diluted with EtOAc. The phases were separated and the aqueous layer was extracted with EtOAc. The combined organic layers were dried with Na_2SO_4, filtered and evaporated. The residue was purified by flash column chromatography on silica gel (eluent: hexane:EtOAc 7:3) to provide **38** (54 mg, 81%) as a colorless oil.

$[\alpha]_D^{22}$= -4.7 (*c* = 0.15, $CHCl_3$). ^{1}H NMR ($CDCl_3$, 400 MHz): δ = 1.37 (d, *J* = 7.1 Hz, 3 H, CH_3), 2.46 (dd, *J* = 7.5, 16.7 Hz, 1 H, CH_2), 2.53 (dd, *J* = 6.5, 16.7 Hz, 1 H, CH_2), 3.08 (sext., *J* = 7.1 Hz, 1 H, CH), 7.15-7.29 (m 5 H, Ph) ppm.

The spectroscopic data correspond with those previously reported[6].

[6] Kangani, C. O.; Day, B. W.; Kelley, D. E. *Tetrahedron Lett.* **2007,** *48,* 5933–5937. doi:10.1016/j.tetlet.2007.06.119

(*S*)-2-*tert*-Butyl-3-methoxy-6-(2,2,2-trifluoro-1-methoxy-1-phenylethyl)pyridin-4(1*H*)-yl nonaflate (52)

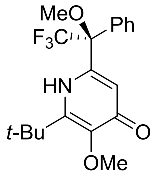

According to procedure 1, methoxyallene (0.15 mL, 1.85 mmol), *n*-BuLi (2.5 M in hexanes, 0.65 mL, 1.63 mmol), pivalonitrile (51 mg, 0.60 mmol) and (*S*)-α-methoxy-(trifluoromethyl)phenylacetic acid (843 mg, 3.60 mmol – dissolved in the minimum amount of DMF were reacted to afford the intermediate β-ketoenamide, which was then treated with TMSOTf (0.70 mL, 3.60 mmol) and NEt_3 (0.50 mL, 3.60 mmol). The crude material was purified by flash column chromatography on silica gel (eluent: hexane/ethyl acetate 80:20) to give 68 mg (30%) of **20** as a colorless oil. Compound **20** was found to be in equilibrium with the corresponding pyridone. The ratio estimated by ^{1}H NMR ($CDCl_3$) is 25:75 in favour of the pyridine tautomer. Pyridone: ^{1}H NMR (250 MHz, $CDCl_3$): δ = 1.43 (s, 9 H, *t*-Bu), 3.39 (br. s, 3 H, OMe), 3.93 (s, 3 H, OMe at C-3), 6.09 (s, 1 H, 5-H), 7.35-7.50 (m, 5 H, Ph), 8.91 (br. s, 1 H, NH) ppm. Pyridinol: ^{1}H NMR (250 MHz, $CDCl_3$): δ = 1.17 (s, 9 H, *t*-Bu), 3.39 (br. s, 3 H, OMe), 3.52 (s, 3 H, OMe at C-3), 7.35-7.50 (m, 6 H, Ph, 5-H), 7.99 (br. s, 1 H, OH) ppm.

Since **20** could not completely be separated from unidentified side products, it was converted into the corresponding nonaflate **52**. Compound **20** (68 mg, 0.18 mmol) was dissolved in dry THF (0.11 mL) and treated with NaH (15 mg, 0.37 mmol). NfF (67 μL, 0.37 mmol) was added and stirred for 10 h. After complete conversion of the starting

material (as indicated by TLC), the reaction was quenched by the addition of MeOH. The aqueous layer was extracted with Et_2O, dried with Na_2SO_4, filtered and the solvent removed under reduced pressure. The crude product was purified by flash column chromatography on silica gel (eluent: hexane/ethyl acetate 100:0 to 70:30, linear gradient) to afford 61 mg (56%) of **52** as a colorless oil.

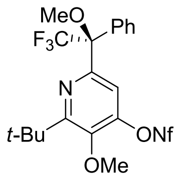

$[\alpha]_D^{22}$ = +14.5 (c = 2.9, $CHCl_3$). ^{1}H NMR (500 MHz, $CDCl_3$): δ = 1.34 (s, 9 H, *t*-Bu), 3.42 (s, 3 H, OMe), 3.94 (m_c, 3 H, OMe at C-3), 7.32-7.35, 7.37-7.42 (2 m, 5 H, Ph) ppm. ^{13}C NMR (101 MHz, $CDCl_3$): δ = 29.3, 39.1 (q, s, *t*-Bu), 54.3 (br. q, OMe), 61.9 (q, OMe at C-3), 84.5 (s, C-1'), 115.0 (d, C-5), 124.8 (q, J_{CF} = 290.7 Hz, CF_3), 127.9, 128.2, 128.6, 136.6 (3 d, s, Ph), 146.7, 150.2, 151.7 (3 s, C-2, C-3, C-6), 163.6 (s, C-4) ppm. ^{19}F NMR (376 MHz, $CDCl_3$): δ = -68.6 (s, CF_3), -80.5 (m_c, CF_3), -109.4 (m_c, CF_2), -120.6 (m_c, CF_2), -125.7 (m_c, CF_2) ppm. IR (neat): $\tilde{\nu}$ = 2980, 2930 (C-H), 1560, 1430 (C=C), 1200, 1140, 1030, 725 (C-F) cm^{-1}. ESI-TOF: *m/z* calc. for $[C_{19}H_{22}F_3NO_2]^+$ 652.1022, found 652.0994.

(*S*)-2-*tert*-Butyl-6-[(*tert*-butyldimethylsiloxy)phenylmethyl]-3-methoxypyridin-4(1*H*)-one (22)

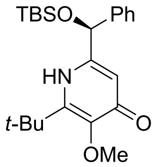

According to procedure 1, methoxyallene (0.25 mL, 3.08 mmol), *n*-BuLi (2.5 M in hexanes, 1.16 mL, 2.90 mmol), pivalonitrile (83 mg, 1.00 mmol) and TBS-mandelic acid **21** (1.60 g, 6.00 mmol) were converted into the β-ketoenamide, which was then treated with NEt_3 (1.66 mL, 12.0 mmol) and TMSOTf (2.61 mL, 12.0 mmol). The crude material was purified by flash column chromatography on silica gel (eluent: hexane/ethyl acetate 50:50 to 70:30, linear gradient) to afford 201 mg (50%) of **22** as a yellow oil. $[\alpha]_D^{22}$ = -7.1 (c = 10.7, $CHCl_3$). ^{1}H NMR (500 MHz, $CDCl_3$): δ = -0.08, 0.07, 0.90 (3 s, 3 H, 3 H, 9 H, OTBS), 1.40 (s, 9 H, *t*-Bu), 3.89 (s, 3 H, OMe), 5.56 (s, 1 H, 1'-H), 6.13 (s, 1 H, 5-H), 7.26-7.35 (m, 5 H, Ph), 8.83 (s, 1 H, NH) ppm. ^{13}C NMR (101 MHz, $CDCl_3$): δ = -4.9, -4.7, 18.2, 25.8 (3 q, s, OTBS), 28.5, 35.3 (q, s, *t*-Bu), 58.8 (q, OMe), 73.4 (d, C-1'), 113.9 (d, C-5), 126.4, 128.7, 128.9, 140.9 (3 d, s, Ph), 145.6, 146.7, 147.1 (3 s, C-2, C-3, C-6), 175.5 (s, C-4) ppm. IR (neat): $\tilde{\nu}$ = 3355 (N-H), 2940, 2930, 2855 (C-H), 1620 (C=O), 1250 (C=C), 1000 (C-N) cm^{-1}. ESI-TOF: *m/z* calc. for $[M+H]^+$ 402.2464, found 402.2472. Anal. calc. for $C_{23}H_{35}NO_2Si$ (401.6): C 68.78, H 8.78, N 3.49, found: C 68.48, H 8.84, N 3.55.

(*S*)-2-*tert*-Butyl-6-(1-dibenzylamino-2-phenylethyl)-3-methoxypyridin-4(1*H*)-one (24)

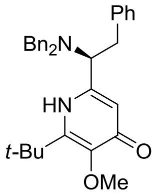

Compound **24** was prepared according to procedure 1, from methoxyallene (0.25 mL, 3.08 mmol), *n*-BuLi (2.5 M in hexanes, 1.16 mL, 2.90 mmol), pivalonitrile (83 mg, 1.00 mmol) and *N,N*-dibenzylated phenylalanine (**23**) (2.15 g, 6.00 mmol). The resulting β-ketoenamide was treated with NEt_3 (0.83 mL, 6.00 mmol) and TMSOTf (1.31 mL, 6.00 mmol). The crude material was purified by flash column chromatography on silica gel (eluent: hexane/ethyl acetate 50:50) to afford 208 mg (45%) of **24** as a brown, highly viscous oil. $[\alpha]_D^{22}$ = +50.0 (c = 0.2, $CHCl_3$). ^{1}H NMR (500 MHz, $CDCl_3$): δ = 1.36 (s, 9 H, *t*-Bu), 3.12 (dd, *J* = 14.6, 9.0 Hz, 1 H, 2'-H), 3.39 (dd, *J* = 14.6, 4.1 Hz, 1 H, 2'-H), 3.54, 3.80 (2 d, *J* = 13.3 Hz, 2 H each, NCH_2), 3.88 (s, 3 H, OMe), 3.96 (dd, *J* = 9.0, 4.1 Hz, 1 H, 1'-H), 6.67 (s, 1 H, 5-H), 7.15-7.35 (m, 15 H, Ph), 8.82 (s, 1 H, NH) ppm. ^{13}C NMR (101 MHz, $CDCl_3$): δ = 28.4 (q, *t*-Bu), 30.9 (t, C-2'), 35.6 (s, *t*-Bu), 53.9 (d, NCH_2), 58.9 (q, OMe), 60.1 (d, C-1'), 116.4 (d, C-5), 126.8, 127.9, 128.9, 129.0, 129.1, 129.2 (6 d, Ph), 138.2, 138.6 (2 s, Ph), 145.8, 146.2, 147.3 (3 s, C-2, C-3, C-6), 175.4 (s, C-4) ppm. IR (neat): $\tilde{\nu}$ = 3685, 3620 (N-H), 3020, 2975 (C-H), 1720 (C=O), 1520, 1425, 1215 (C=C) cm^{-1}. ESI-TOF: *m/z* calc. for $[M+H]^+$ 481.2849, found 481.2842. Anal. calc. for $C_{32}H_{36}N_2O_2$ (480.6): C 79.96, H 7.55, N 5.83, found: C 78.18, H 7.41, N 5.63.

rac-2-tert-Butyl-6-(1,1-dibenzylamino-2-methylpropyl)-3-methoxypyridin-4(1H)-one (26)

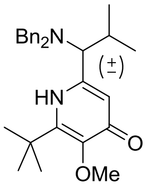

According to procedure 1, methoxyallene (83 µL, 0.99 mmol), *n*-BuLi (2.5 M in hexanes, 0.36 mL, 0.90 mmol), pivalonitrile (42 mg, 0.50 mmol) and *rac-N,N*-dibenzyl valine (**25**) (588 mg, 2.00 mmol) were reacted to afford the intermediate β-ketoenamide, which was subsequently treated with NEt_3 (0.83 mL, 6.00 mmol) and TMSOTf (1.31 mL, 6.00 mmol). The crude material was purified by flash column chromatography on silica gel (eluent: hexane/ethyl acetate 80:20) to afford 108 mg (50%) of **26** as a colorless oil. ^{1}H NMR (500 MHz, $CDCl_3$): δ = 0.74, 1.13 (2 d, *J* = 6.5 Hz, 3 H each, Me), 1.38 (s, 9 H, *t*-Bu), 2.28 (hept.d, *J* = 6.5, 8.3 Hz, 1 H, 2'-H), 3.12 (d, *J* = 8.3 Hz, 1 H, 1'-H), 3.37, 3.86 (d, *J* = 14.1 Hz, 2 H each, NCH_2), 3.98 (s, 3 H, OMe), 6.33 (s, 1 H, 5-H), 7.22-7.35 (m, 10 H, Ph), 7.91 (s, 1 H, NH) ppm. ^{13}C NMR (125 MHz, $CDCl_3$): δ = 19.3, 20.6 (2 q, Me), 28.4 (q, *t*-Bu), 28.8 (d, C-2'), 35.1 (s, *t*-Bu), 53.7 (t, NCH_2), 58.9 (q, OMe), 68.1 (d, C-1'), 116.9 (d, C-5), 127.5, 128.7, 128.8, 138.3 (3 d, s, Ph), 143.2, 145.9, 147.0 (3 s, C-2, C-3, C-6), 175.6 (s, C-4) ppm. IR (neat): $\tilde{\nu}$ = 3050-2930 (C-H), 1725 (C=O), 1420-1265 (C=C) cm^{-1}. ESI-TOF: *m/z* calc. for $[C_{28}H_{37}N_2O_2]^+$ 433.2855, found 433.2871.

(*R*)-2-*tert*-Butyl-3-methoxy-6-(1-phenylpropyl)pyridin-4-ol (28)

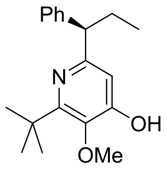

According to procedure 1, methoxyallene (92 µL, 1.13 mmol) was treated with *n*-BuLi (2.5 M in hexanes, 0.46 mL, 1.15 mmol) and reacted with pivalonitrile (89 mg, 1.07 mmol) and (*R*)-2-phenylbutyric acid **27** (0.35 mL, 3.21 mmol). The crude product was cyclized with TMSOTf (1.42 mL, 6.42 mmol) and NEt_3 (0.89 mL, 6.42 mmol) in CH_2Cl_2 (7 mL) at 50 °C for 3 d. The title compound (143 mg, 45%) was obtained in pure form after flash column chromatography on silica gel (eluent: EtOAc). $[\alpha]_D^{22}$ = -8.1 (c = 0.7, MeOH). ^{1}H NMR (500 MHz, CD_3OD + $CDCl_3$): δ = 0.92 (t, *J* = 7.3 Hz, 3 H, CH_3), 1.35 (s, 9 H, *t*-Bu), 2.01-2.12 (m, 2 H, CH_2), 3.82 (s, 3 H, OMe), 4.04 (t, *J* = 7.8 Hz, 1 H, PhC*H*), 6.44 (s, 1 H, 5-H), 7.25-7.36 (m, 5 H, Ph) ppm. ^{13}C NMR (101 MHz, CD_3OD + $CDCl_3$): δ = 11.3 (q, C-3'), 27.0 (t, C-2'), 27.6 (q, *t*-Bu), 35.4 (s, *t*-Bu), 49.6 (d, C-1'), 58.5 (q, OMe), 113.3 (d, C-5), 127.2, 127.7, 128.8 (3 d ,Ph), 126.7, 127.5, 128.1, 140.6, 151.9 (5 s, Ph, C-2, C-3, C-4, C-6) ppm. IR (neat): $\tilde{\nu}$ = 3375 (OH), 2965-2875 (=C-H, C-H), 1620-1535 (C=C) cm^{-1}. ESI-TOF: *m/z* calc. for $[C_{19}H_{26}NO_2]^+$ 300.1985, found 300.1957.

(*R*)-6-[(*tert*-Butyldimethylsiloxy)phenylmethyl]-3-methoxy-2-phenylpyridin-4(1*H*)-one (30)

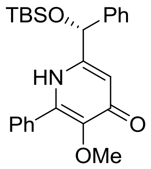

According to procedure 1, methoxyallene (0.25 mL, 3.1 mmol), *n*-BuLi (2.5 M in hexanes, 1.16 mL, 2.90 mmol), freshly distilled benzonitrile (102 μL, 1.00 mmol) and TBS-mandelic acid (**29**) (1.60 g, 6.00 mmol) were converted into the β-ketoenamide, which was then treated with NEt_3 (1.66 mL, 12.0 mmol) and TMSOTf (2.61 mL, 12.0 mmol). The crude product was purified by flash column chromatography on silica gel (eluent: hexane/ethyl acetate 100:0 to 50:50, linear gradient) to afford **30** as a brown, viscous oil (101 mg, 24%). $[\alpha]_D^{22}$ = 32.1 (c = 1.0 $CHCl_3$). ^{1}H NMR (500 MHz, $CDCl_3$): δ = -0.06, 0.09, 0.91 (s, 3 H, 3 H, 9 H, OTBS), 3.75 (br. s, 3 H, OMe), 5.63 (br. s, 1 H, 1'-H), 6.25 (br. s, 1 H, 5-H), 7.27 -7.66 (m, 10 H, Ph), 8.88 (br. s, 1 H, NH) ppm. ^{13}C NMR (101 MHz, $CDCl_3$): δ = -5.0, -4.7, 18.2, 25.8 (3 s, q, OTBS), 59.8 (q, OMe), 122.0 (d, C-5), 126.4, 128.1, 128.9 (3 d, Ph), 175.5 (br. s, C-4) ppm. IR (neat): $\tilde{\nu}$ = 3535 (N-H), 2970, 2875 (C-H), 1735 (C=O), 1470-1195 (C=C) cm^{-1}. ESI-TOF: *m/z* calc. for $[C_{25}H_{32}NO_3Si]^+$ 422.2146, found 422.2144.

(*S,S*)-2,6-Di-*sec*-butyl-3-methoxypyridin-4(1*H*)-one (32)

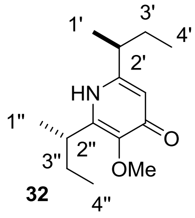

32

According to procedure 1, methoxyallene (0.38 mL, 4.7 mmol), *n*-BuLi (2.5 M in hexanes, 1.65 mL, 4.13 mmol), (*S*)-2-methylbutyronitrile (**31**) (162 µL, 1.53 mmol) and (*S*)-2-methylbutyric acid (**17**) (1.0 mL, 9.18 mmol) were converted into the β-ketoenamide, which was then treated with NEt_3 (1.91 mL, 13.8 mmol) and TMSOTf (3.01 mL, 13.8 mmol). The crude material was purified by flash column chromatography on silica gel (eluent: CH_2Cl_2:MeOH 20:1) to afford **18** as a brown, amorphous solid (309 mg, 85%). mp 113-116 °C. $[\alpha]_D^{22}$ = +39.3 (c = 1.5, $CHCl_3$). ^{1}H NMR (500 MHz, $CDCl_3$): δ = 0.85 (t, *J* ≈ 7 Hz, 3 H, 4’/4’’-H), 0.87 (t, *J* = 6.5 Hz, 3 H, 4’/4’’-H), 1.21, 1.27 (2 m_c, 3 H each, 1’-H/1’’-H), 1.56, 1.66 (2 m_c, 2 H each, 3’-H/3’’-H), 2.59, 3.19 (2 m_c, 1 H each, 2’-H/2’’-H), 3.86 (s, 3 H, OMe), 6.26 (s, 1 H, 5-H), 8.91 (br. s, 1 H, NH) ppm. ^{13}C NMR (125 MHz, $CDCl_3$): δ = 11.7, 12.4 (2 q, C-4’/C-4’’), 18.9, 19.9 (2 q, C-1’/ C-1’’), 27.8, 29.9 (2 t, C-3’/C-3’’), 35.2, 38.2 (2 d, C-2’/C-2’’), 59.6 (q, OMe), 112.3 (d, C-5), 145.3, 147.6, 155.2 (3 s, C-2, C-3, C-6), 174.5 (s, C-4) ppm. IR (ATR): $\tilde{\nu}$ = 3255 (NH), 3075-2825 (=C-H, C-H), 1610 (C=O), 1532-1200 (C=C) cm^{-1}. ESI-TOF: *m/z* calc. for $[C_{14}H_{24}NO_2]^+$ 238.1807, found 238.1806.

2,6-Bis{(2*R,6R*)-[*tert*-butyldimethylsiloxy)(phenyl)methyl]-3-methoxypyridin-4(1*H*)-one (35)

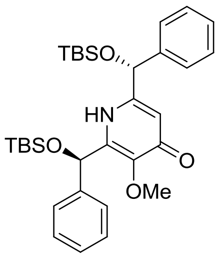

A solution of *n*-BuLi (2.5 M in hexanes, 1.58 mL, 3.95 mmol) was added dropwise to a solution of methoxyallene (0.36 mL, 4.4 mmol) in Et_2O (10 mL) at -40 °C. After 30 min, *rac*-2-(*tert*-butyldimethylsiloxy)-2-phenylacetonitrile (**33**) (360 mg, 1.46 mmol) was added and the mixture stirred at -50 °C for 3 h. Then the mixture was cooled to -78 °C and (*R*)-2-(*tert*-butyldimethylsiloxy)-2-phenylacetic acid (**29**) (2.33 g, 8.75 mmol) dissolved in Et_2O (2 mL) added. The mixture was stirred overnight during which time it was slowly allowed to reach r.t. The reaction was quenched by the addition of aq. sat. $NaHCO_3$ (15 mL), the phases were separated and the aqueous layer was extracted with Et_2O (3x20 mL). The combined organic layers were dried with Na_2SO_4, filtered and the solvent was removed under reduced pressure. The crude product was purified by flash column chromatography (silica gel, hexane/EtOAc = 9:1 to 8:2) to provide 482 mg (58%) of the β-methoxy-β-ketoenamide. Following the typical procedure, the β-methoxy-β-ketoenamide (70 mg, 0.12 mmol) was cyclized with TMSOTf (0.07 mL, 0.36 mmol) and NEt_3 (0.06 mL, 0.36 mmol) in 1,2-dichloroethane (2.5 mL) at 70 °C for 1 d. The title compound and the corresponding diastereomer (15 mg each, 22% each; 26% over 2 steps for both diastereomers) were obtained pure after flash column chromatography on

silica gel (eluent: hexane: EtOAc 8:2 to 7:3 linear gradient). $[\alpha]_D^{22}$ = -47.1 (c = 0.7, $CHCl_3$). ^{1}H NMR (700 MHz, $CDCl_3$): δ = -0.03, 0.03, 0.07, 0.10 (4 s, 3 H each, OTBS), 0.90, 0.94 (2 s, 9 H each, OTBS), 3.72 (s, 3 H, OMe), 5.57, 6.12 (2 s, 1 H, each, C*H*Ph), 6.24 (s, 1 H, 5-H), 7.23-7.36 (m, 10 H, Ph) ppm. ^{13}C NMR (176 MHz, $CDCl_3$): δ = -5.05, -4.98, -4.84, -4.77 (4 q, OTBS), 18.23, 18.27 (2 s, OTBS), 25.8 (q, OTBS), 58.9 (q, OMe), 69.0, 72.7 (2 d, *C*HPh), 114.5 (d, C-5), 126.0, 126.1, 128.1, 128.5, 128.6, 128.8 (6 d, Ph), 140.8 (s, Ph), 141.2 (s, C-3), 143.5, 147.9 (s, C-2/6), 175.0 (s, C-4) ppm. IR (neat): $\tilde{\nu}$ = 3360 (NH), 3145-2835 (=C-H, C-H), 1705-1550 (C=O, C=C) cm^{-1}. ESI-TOF: *m/z* calc. for $[C_{32}H_{48}NO_4Si_2]^+$ 566.3116, found 566.3145.

6-{(*R*)-[*tert*-Butyldimethylsiloxy)(phenyl]methyl}-2-{(*S*)-[*tert*-butyldimethylsilyloxy]-(phenyl)methyl}-3-methoxypyridin-4(1*H*)-one (34)

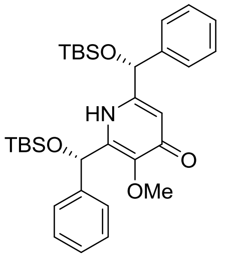

$[\alpha]_D^{22}$ = +41.6 (c = 0.6, $CHCl_3$). ^{1}H NMR (700 MHz, $CDCl_3$): δ = 0.00, 0.02 (2 s, 6 H each, OTBS), 0.90, 0.92 (2 s, 9 H each, OTBS), 3.75 (s, 3 H, OMe), 5.57, 6.11 (2 s, 1 H each, C*H*Ph), 6.26 (s, 1 H, 5-H), 7.25-7.45 (m, 10 H, Ph) ppm. ^{13}C NMR (176 MHz, $CDCl_3$): δ = -5.3, -4.9 (2 q, OTBS), 18.2 (s, OTBS), 25.7 (q, OTBS), 58.9 (q, OMe), 69.0, 72.5 (2 t, CH_2Ph), 114.2 (d, C-5), 125.8, 126.1, 128.1, 128.3, 128.5, 128.8 (6 d, Ph), 140.9 (s,

Ph), 141.1 (s, C-3), 141.3 (s, Ph), 143.7, 147.8 (2 s, C-2/C-6), 174.9 (s, C-4) ppm. IR (neat): $\tilde{\nu}$ = 3360 (NH), 3145-2835 (=C-H, C-H), 1705-1550 (C=O, C=C) $cm^{-1.}$ ESI-TOF: *m/z* calc. for $[C_{32}H_{48}NO_4Si_2]^+$ 566.3116, found 566.3145.

(*R*)-2-[(*tert*-Butyldimethylsiloxy)phenylmethyl]-3-methoxy-6-(trifluoromethyl)-pyridin-4(1*H*)-one (37)

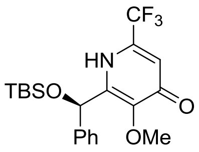

According to procedure 1, methoxyallene (0.25 mL, 3.1 mmol), *n*-BuLi (2.5 M in hexanes, 1.16 mL, 2.90 mmol), TBS-mandelonitrile **36** (247 mg, 1.00 mmol) and trifluoroacetic acid (1.54 mL, 6.00 mmol) were converted to the β-ketoenamide, which was then treated with NEt_3 (0.42 mL, 3.00 mmol) and TMSOTf (0.58 mL, 3.00 mmol). The crude material was purified by flash column chromatography on silica gel (eluent: hexane/ethyl acetate 100:0 to 50:50 linear gradient) to afford **37** as a colorless oil (152 mg, 37%). In $CDCl_3$ **37** was found to be in equilibrium with the corresponding pyridone. At r.t. the ratio pyridinol/pyridone was ≈ 4:6. Pyridinol**.** $[\alpha]_D^{22}$ = 38.4 (c = 1.1, $CHCl_3$). ^1H NMR (500 MHz, $CDCl_3$): δ = -0.07, 0.06, 0.90 (3 s, 3 H, 3 H, 9 H, OTBS), 3.67 (s, 3 H, OMe), 6.11 (s, 1 H, 1'-H), 7.23 (s, 1 H, 5-H), 7.15-7.50 (m, 5 H, Ph), 9.93 (br. s, 1 H, OH) ppm. ^{13}C NMR (101 MHz, $CDCl_3$): δ = -5.3, -4.7, 18.5, 26.0 (3 q, s, OTBS), 60.8 (q, OMe), 75.4 (d, C-1'), 109.6 (d, C-5), 121.4, 126.1, 127.9, 129.0 (3 d, s, Ph), 141.4 (q, J_{CF} = 265.9 Hz, CF_3), 142.7, 144.3, 157.1, 157.9 (4 s, C-2, C-3, C-4, C-6) ppm. Pyridone: ^1H NMR (500 MHz, $CDCl_3$): δ = -0.08, 0.13, 0.92 (3 s, 3 H, 3 H, 9 H, OTBS), 3.54 (s, 3 H, OMe), 6.00 (s, 1 H, 1'-H), 6.80 (s, 1 H, 5-H), 7.15-7.50 (m, 5 H, Ph), 9.42

(br. s, 1 H, NH) ppm. ^{13}C NMR (101 MHz, $CDCl_3$): δ = -4.9, -4.6, 18.2, 25.8 (3 q, s, OTBS), 58.7 (q, OMe), 69.7 (d, C-1’), 116.1 (d, C-5), 120.1 (q, J_{CF} = 273.8 Hz, CF_3), 126.9, 128.9, 129.0, 133.2 (3 d, s, Ph), 140.0, 142.9, 145.7 (s, C-2, C-3, C-6), 174.4 (s, C-4) ppm. IR (neat): $\tilde{\nu}$ = 3310 (N-H), 2950, 2930, 2860 (C-H), 1605 (C=O), 1405, 1255, 1135 (C=C) cm^{-1}. ESI-TOF: *m/z* calc. for $[C_{20}H_{26}F_3NaNO_3Si]^+$ 436.1532, found 436.1512.

(*S*)-2-(2-Phenylpropyl)-6-(trifluoromethyl)pyridin-4-ol (39)

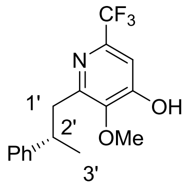

Following procedure 1, methoxyallene (0.19 mL, 2.3 mmol) was treated with *n*-BuLi (2.5 M in hexanes, 0.88 mL, 2.20 mmol) and reacted with (*S*)-3-phenylbutanenitrile (**38**) (86 mg, 0.59 mmol) and trifluoroacetic acid (352 µL, 4.56 mmol). The crude product was cyclized with TMSOTf (316 µL, 2.28 mmol) and NEt_3 (315 µL, 2.28 mmol) in 1,2-dichloroethane (11 mL) at 70 °C for 3 d. Compound **39** (52 mg, 28%) was obtained in pure form after flash column chromatography on silica gel (eluent: hexane: EtOAc 8:2). $[\alpha]_D^{22}$ = +49.1 (c = 0.45, $CHCl_3$).^{1}H NMR (500 MHz, $CDCl_3$): δ = 1.34 (d, *J* = 6.6 Hz, 3 H, 3’-H), 3.02-3.07 (m, 2 H, 1’-H), 3.40-3.43 (m, 1 H, 2’-H), 3.68 (s, 3 H, OMe), 7.06 (s, 1 H, 5-H), 7.14-7.24 (m, 5 H, Ph) ppm. ^{13}C NMR (101 MHz, $CDCl_3$): δ = 21.3 (q, C-3’), 38.8 (t, C-1’), 40.3 (d, C-2’), 61.2 (q, OMe), 107.5 (d, C-5), 144.5, 146.5, 155.2, 156.5 (4 s, C-2, C-3, C-4, C-5) ppm. *The CF_3 group could not be detected. ^{19}F NMR (470 MHz,

$CDCl_3$): δ = -67.3 (s, CF_3) ppm. IR (neat): $\tilde{\nu}$ = 2960- 2565 (=C-H, C-H), 1610-1495 (C=C) cm^{-1}. ESI-TOF: *m/z* calc. for $[C_{16}H_{17}F_3NO_2]^+$ 312.1206, found 312.1218.

(*S*)-2-*sec*-Butyl-3-methoxy-6-(trifluoromethyl)pyridin-4(1*H*)-one (40)

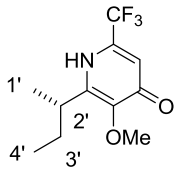

According to procedure 1, methoxyallene (0.75 mL, 9.3 mmol), *n*-BuLi (2.5 M in hexanes, 3.24 mL, 8.10 mmol), (*S*)-2-methylbutyronitrile (**31**) (0.32 mL, 3.00 mmol) and trifluoroacetic acid (1.34 mL, 18.0 mmol) were converted into the β-ketoenamide which was then treated with NEt_3 (1.25 mL, 9.00 mmol) and TMSOTf (1.74 mL, 9.00 mmol). The crude material was purified by flash column chromatography on silica gel (eluent: hexane/EtOAc 100:0 to 70:30, linear gradient) to afford **40** as a yellow oil (421 mg, 56%). $[\alpha]_D^{22}$ = +21.3 (c = 6.8, $CHCl_3$), ^{1}H NMR (500 MHz, $CDCl_3$): δ = 0.82 (t, *J* = 7.4 Hz, 3 H, 4'-H), 1.25 (d, *J* = 6.8 Hz, 3 H, 1'-H), 1.58-1.67, 1.75-1.84 (2 m, 1 H each, 3'-H), 3.18-3.23 (m, 1 H, 2'-H), 3.85 (s, 3 H, OMe), 6.62 (s, 1 H, 5-H), 7.09 (s, 1 H, OH) ppm. ^{1}H NMR (500 MHz, CD_3OD): δ = 0.79 (t, *J* = 7.4 Hz, 3 H, 4'-H), 1.20 (d, *J* = 6.9 Hz, 3 H, 1'-H), 1.53-1.62, 1.74-1.82 (2 m, 1-H each, 3'-H), 3.17-3.25 (m, 1 H, 2'-H), 3.83 (s, 3 H, OMe), 7.02 (s, 1 H, 5-H) ppm. ^{13}C NMR (101 MHz, $CDCl_3$): δ = 12.3 (q, C-4'), 19.8 (q, C-1'), 28.9 (t, C-3'), 36.2 (d, C-2'), 61.9 (q, OMe), 107.3 (d, C-5), 121.3 (q, J_{CF} = 273.8 Hz, CF_3), 143.9 (m_c, higher intensity, C-4, C-6), 156.7, 160.1 (2 s, C-2, C-3) ppm. ^{19}F NMR (376 MHz, $CDCl_3$): δ = -63.0 (s, CF_3) ppm. IR (neat): $\tilde{\nu}$ = 2965-2840 (C-H, =C-H), 1605-1420 (C=C), 1130 (C-N) cm^{-1}. ESI-TOF**:** *m/z* calc. for $[M+H]^+$ 250.1055, found

250.1050. Anal. calc. for $C_{11}H_{14}F_3NO_2$ (249.2): C 53.01, H 5.66, N 5.62, found: C 52.72, H 5.17, N 6.01.

(*S*)-(3-Methoxy-5,5-dimethyl-4-oxohex-1-en-2-yl) 1-tritylpyrrolidine-2-carboxylate (48)

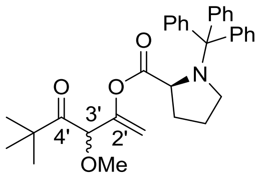

Following procedure 1, methoxyallene (0.14 mL, 1.7 mmol) was treated with *n*-BuLi (2.5 M in hexanes, 0.67 mL, 1.68 mmol) and reacted with pivalonitrile (80 mg, 0.96 mmol) and (*S*)-trityl-proline **45** (1.20 g, 3.36 mmol). The crude product was purified by flash column chromatography on silica gel (hexane:EtOAc 8:2) to afford **48** (140 mg, 49%) as a 1:1 mixture of two diastereomers and **47** (26 mg, 9%) as colorless oils. ^{1}H NMR (400 MHz, $CDCl_3$): δ = 0.84-1.07 (m 4 H, 3-H or 4-H), 1.19, 1.21 (2 s, 9 H each, *t*-Bu), 1.55-1.66 (m, 4 H, 3-H or 4-H), 2.80-2.92, 3.30-3.43 (2 m, 2 H each, 5-H), 3.40, 3.44 (2 s, 3 H each, OMe), 4.51, 4.57 (2 s, 1 H each, 3'-H), 5.17-5.21 (m, 4 H, $=CH_2$), 7.14-7.28 (m, 20 H, Ph), 7.55-7.60 (m, 10 H, Ph) ppm.^{13}C NMR (101 MHz, $CDCl_3$): δ = 24.1, 24.2 (2 s, *t*-Bu), 26.4, 26.5 (2 q, *t*-Bu), 31.1, 31.2 (2 t, C-3 or C-4), 44.3, 44.4 (2 t, C-3, C-4), 49.9 (t, C-5), 57.5, 57.6 (2 q, OMe), 62.95, 63.04 (2 d, C-2), 82.3, 82.5 (2 d, C-3'), 89.4, 89.5 (2 s, Ph), 106.8, 107.0 (2 t, $=CH_2$), 126.2 (d, Ph), 127.66, 127.68, 129.1, 129.2 (4 d, Ph), 144.64, 144.65 (2 s, Ph), 149.4, 149.5 (2 s, C-2'), 174.3, 174.5 (2 s, *C*O_2R), 208.9, 209.0 (2 s, *C*=O) ppm. IR (neat): $\tilde{\nu}$ = 3020-2980 (C-H, =C-H), 2300-2350 (C=C), 1710 (C=O) cm^{-1}. ESI-TOF: *m/z* calc. for $[C_{33}H_{38}NO_4]^+$ 534.2620, found 534.2542.

(*S*,*E*)-*N*-(3-Methoxy-5,5-dimethyl-4-oxohex-2-en-2-yl)-1-tritylpyrrolidine-2-carboxamide (47)

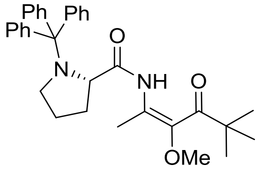

^{1}H NMR (250 MHz, $CDCl_3$): δ = 0.77-0.86 (m, 2 H, 3-H or 4-H), 1.38 (s 9 H, *t*-Bu) , 1.58-1.65 (m, 2 H, 3-H or 4-H), 2.30 (s, 3 H, 1'-H), 3.03-3.14, 3.28-3.40 (2 m ,1 H each, 5-H), 3.54 (s, 3 H, OMe), 3.92-3.95 (m, 1 H, 2-H), 7.12-7.29 (m, 10 H, Ph), 7.46-7.49 (m, 5 H, Ph) ppm.

(*S,S*)-2-*tert*-Butyl-6-[(*tert*-butyldimethylsiloxy)phenylmethyl]-3-methoxypyridin-4-yl 3,3,3-trifluoro-2-methoxy-2-phenylpropanoate (49)

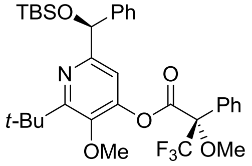

According to procedure 4, pyridone **22** (22 mg, 0.06 mmol) was treated with (*S*)-Mosher chloride (17 µL, 0.09 mmol) to give 25 mg (68%) of NMR-spectroscopically pure **49** as a colorless oil after an aqueous work-up. $[\alpha]_D^{22}$ = +14.0 (c = 1.0, $CHCl_3$) ^{1}H NMR (500 MHz, $CDCl_3$)**:** δ = 0.01, 0.02, 0.96 (s, 3 H, 3 H, 9 H, OTBS), 1.35 (s, 9 H, *t*-Bu), 3.49, 3.68 (2 s, 3 H each, OMe), 5.80 (s, 1 H, 1'-H), 7.17 (s, 1 H, 5-H), 7.19-7.22, 7.27-7.31, 7.48-7.53, 7.61-7.71 (4 m, 10 H, Ph) ppm. ^{19}F NMR (376 MHz, $CDCl_3$): δ = -71.1 (s, CF_3) ppm. IR (neat): $\tilde{\nu}$ = 2955-2930 (C-H), 1775 (C=O), 1570-1450 (C=C), 1170-1105

(=C-H), 780-700 (C-F) cm^{-1}. ESI-TOF: *m/z* calc.for $[C_{33}H_{43}F_3NO_5Si]^+$ 618.2857, found 618.2896.

(*S,S*)-6-*sec*-Butyl-2-*tert*-butyl-3-methoxypyridin-4-yl 3,3,3-trifluoro-2-methoxy-2-phenylpropanoate (50)

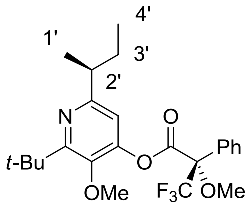

According to procedure 4, pyridone **18** (5 mg, 0.02 mmol) was treated with (*S*)-Mosher chloride (4 µL, 0.02 mmol) to give 5 mg (55%) of NMR-spectroscopically pure **50** as a colorless oil after an aqueous work-up. $[\alpha]_D^{22}$ = +22.4 (c = 2.5, $CHCl_3$). ^{1}H NMR (500 MHz, C_6D_6): δ = 0.78 (t, *J* = 7.4 Hz, 3 H, 4'-H), 1.20 (d, *J* = 6.9 Hz, 3 H, 1'-H), 1.46 (s, 9 H, *t*-Bu), 1.50, 1.75 (2 dqd, *J* = 6.9, 7.4, 15.6 Hz, 1 H each, 3'-H), 2.61 (sext., *J* = 6.9 Hz, 1 H, 2'-H), 3.18 (s, 3 H, OMe), 3.91 (br. s, 3 H, OMe), 6.87 (s, 1 H, 5-H), 6.95-7.05 (m, 10 H, Ph) ppm. ^{19}F NMR (376 MHz, C_6D_6): δ = -71.2 (s, CF_3) ppm. MS (EI, 80 eV, 80 °C): *m/z* (%) = 453 (16) $[M]^+$, 438 (27) $[M - CH_3]^+$, 264 (23), 236 (75), 189 (100).

(*S,S*)-2-*sec*-Butyl-3-methoxy-6-(trifluoromethyl)pyridin-4-yl 3,3,3-trifluoro-2-methoxy-2-phenylpropanoate (51)

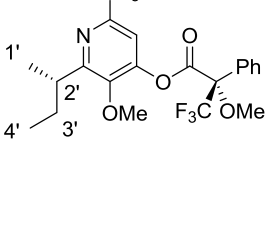

According to procedure 4, pyridine **40** (37 mg, 0.14 mmol) was treated with (*S*)-Mosher chloride (42 µL, 0.22 mmol) to give 39 mg (67%) of NMR-spectroscopically pure **51** as a colorless oil after an aqueous work-up. $[\alpha]_D^{22}$ = +22.7 (c = 0.75, $CHCl_3$). ^{1}H NMR (500 MHz, C_6D_6): δ = 0.68 (t, *J* = 7.4, 3 H, 4'-H), 1.13 (d, *J* = 6.8 Hz, 3 H, 1'-H), 1.48 (dqd, *J* = 6.6, 7.4, 13.8 Hz, 1 H, 3'-H), 1.79 (quint.d, *J* = 7.4, 13.8 Hz, 1 H, 3'-H), 3.03 (dqd, *J* = 6.6, 6.8, 7.4 Hz, 1 H, 2'-H), 3.09 (s, 3 H, OMe), 3.32 (br. s, 3 H, OMe), 6.98-7.04 (m, 3 H, Ph), 7.10 (s, 1 H, 5-H), 7.60-7.62 (m, 2 H, Ph) ppm. ^{19}F NMR (376 MHz, C_6D_6)**:** δ = -67.5, -71.2 (2 s, CF_3) ppm. IR (neat): $\tilde{\nu}$ = 2965-2880 (C-H), 1780 (C=O), 1585, 1470, 1460 (C=C), 1170, 1140, 990, 960 (C-H), 730-660 (C-F) cm^{-1}. ESI-TOF: *m/z* calc. for $[C_{20}H_{20}F_6NO_3]^+$ 466.1448, found 466.1462.

General Procedure for the *O*-Methylation of 4-Hydroxypyridines (Procedure 5)

NaH in mineral oil (60 wt-%, 3.0 equiv.) was washed with hexane (three times) and suspended in anhydrous THF (2.0 mL/mmol 4-hydroxypyridine). A solution of the 4-hydroxypyridine in THF (2.0 mL/mmol 4-hydroxypyridine) was added dropwise. After 5 min stirring at r.t., methyl iodide (3.0 equiv.) was added and the reaction mixture stirred at ambient temperature overnight. After complete consumption of the starting material (as indicated by TLC), the reaction was quenched by the slow addition of MeOH until hydrogen production had ceased. Water and ethyl acetate were added, the organic layer was separated and the aqueous phase extracted with ethyl acetate. The combined organic layers were dried with anhydrous Na_2SO_4, filtered and the solvent was removed under reduced pressure. The crude material was purified by flash column

chromatography on silica gel using an appropriate mixture of ethyl acetate and hexane as eluent.

(*S*)-2-*tert*-Butyl-6-[(*tert*-butyldimethylsiloxy)phenylmethyl]-3,4-dimethoxypyridine (53)

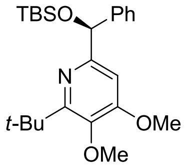

According to procedure 5, **22** (122 mg, 0.30 mmol) was treated with NaH (60 wt-%, 37 mg, 0.91 mmol) and MeI (56 µL, 0.91 mmol). Flash column chromatography of the crude material on silica gel (eluent: hexane/ethyl acetate 100:0 to 50:50, linear gradient) afforded **53** (93 mg, 75%) as a colorless oil. $[\alpha]_D^{22}$ = +12.9 (c = 1.0, $CHCl_3$). ^{1}H NMR (500 MHz, $CDCl_3$): δ = -0.0, 0.04, 0.96 (3 s, 3 H, 3 H, 9 H, OTBS), 1.37 (s, 9 H, *t*-Bu), 3.79, 3.82 (2 s, 3 H each, OMe), 5.78 (s, 1 H, 1'-H), 6.93 (s, 1 H, 5-H), 7.16-7.23, 7.26-7.31, 7.50-7.56 (3 m, 5 H, Ph) ppm. ^{13}C NMR (126 MHz, $CDCl_3$)**:** δ = -4.8, 18.4, 25.9 (2 q, s, OTBS), 29.7, 37.9 (q, s, *t*-Bu), 55.4, 60.4 (2 s, OMe), 101.9 (d, C-5), 126.0, 126.8, 127.9, 142.9, 144.4, 158.1 (3 d, 3 s, Ph, C-2, C-6), 159.2, 159.4 (2 s, C-3, C-4) ppm. IR (neat): $\tilde{\nu}$ = 3050-2800 (C-H, C=C), 1550-1200 (C=C, C-H) cm^{-1}. ESI-TOF: *m/z* calc. for $[M+H]^+$: 416.2621, found 416.2618. Anal. calc. for $C_{24}H_{37}NO_3Si$ (415.6) C 69.35, H 8.97, N 3.37, found: C 72.86, H 9.06, N 2.78.

(*S*)-(6-*tert*-Butyl-4,5-dimethoxypyridin-2-yl)phenylmethanol (54)

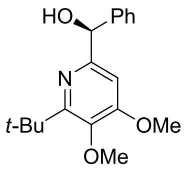

To a solution of **53** (63 mg, 0.15 mmol) in anhydrous THF (1.15 mL), was added HF·pyridine (0.30 mL) and the mixture stirred at r.t. for 15 min. After complete consumption of the starting material (as indicated by TLC), the reaction mixture was diluted with ethyl acetate and H_2O was added. The organic layer was separated and the aqueous layer extracted with ethyl acetate. The combined organic layers were dried with Na_2SO_4, filtered and the solvent was removed under reduced pressure to afford pure **54** (35 mg, 77%) as a colorless oil. $[\alpha]_D^{22}$= -109.0 (c = 1.2, $CHCl_3$). ^{1}H NMR (500 MHz, $CDCl_3$): δ = 1.50 (s, 9 H, *t*-Bu), 3.90, 3.97 (2 s, 3 H each, OMe), 6.05 (s, 1 H, 1'-H), 7.00 (s, 1 H, 5-H), 7.30-7.39 (m, 5 H, Ph) ppm. ^{13}C NMR (101 MHz, $CDCl_3$): δ = 27.7, 37.0 (q, s, *t*-Bu), 57.8, 61.3 (2 q, OMe), 70.8 (d, C-1'), 106.6 (d, C-5), 127.0, 129.2, 129.2 (3 d, Ph), 139.6, 144.9, 152.6, 153.1 (4 s, Ph, C-2, C-3, C-6), 166.2 (s, C-4) ppm. IR (neat) $\tilde{\nu}$ = 3020-2800 (C-H, C=C), 1550-1215 (C=C) cm^{-1}. ESI-TOF: *m/z* calc. for $[C_{18}H_{24}NO_3]^+$ 302.1756, found 302.1757.

(*R*)-6-[(*tert*-Butyldimethylsiloxy)phenylmethyl]-3,4-dimethoxy-2-phenylpyridine (55)

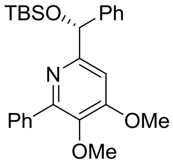

According to procedure 5, **30** (101 mg, 0.24 mmol) was treated with NaH (60 wt-%, 30 mg, 0.72 mmol) and MeI (45 µL, 0.72 mmol). Flash column chromatography of the crude material on silica gel (eluent: hexane/ethyl acetate 80:20) afforded **55** (31 mg, 30%) as a colorless oil. $[\alpha]_D^{22}$= -66.0 (c = 0.5, $CHCl_3$). ^{1}H NMR (500 MHz, $CDCl_3$): δ = 0.02, 0.04, 0.97 (3 s, 3 H, 3 H, 9 H OTBS), 3.59, 3.91 (2 s, 3 H each, OMe), 5.91 (s, 1 H, 1'-H), 7.11 (s, 1 H, 5-H), 7.34-7.29, 7.36-7.39, 7.40-7.46, 7.53-7.64, 7.87-7.91 (5 m, 10 H, Ph) ppm. ^{13}C NMR (101 MHz, $CDCl_3$): δ = -4.7, 18.4, 25.9 (2 q, s, OTBS), 55.7, 60.5 (2 q, OMe), 102.6 (d, C-5), 126.0, 127.1, 128.3, 128.1, 129.3, 137.8 (6 d, Ph), 138.8, 142.4, 144.1, 150.2 (4 s, Ph, C-2, C-6), 159.8, 160.7 (2 s, C-3, C-4) ppm. IR (neat) $\tilde{\nu}$ = 3020-2800 (C-H, C=C), 1550-1215 (C=C). ESI-TOF: *m/z* calc. for $[M+H]^+$ 436.2308, found 436.2308. Anal. calc. for $C_{26}H_{33}NO_3Si$ (435.6): C 71.68, H 7.64, N 3.22, found: C 72.12, H 7.36, N 3.19.

(*R*)-(4,5-Dimethoxy-6-phenylpyridin-2-yl)phenylmethanol (56)

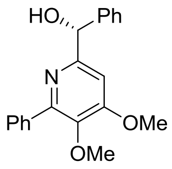

To a solution of **55** (31 mg, 0.07 mmol) in anhydrous THF (1.0 mL), was added HF·pyridine (0.15 mL) and the mixture stirred at r.t. for 40 min. After complete consumption of the starting material (as indicated by TLC), the reaction mixture was diluted with ethyl acetate and H_2O was added. The organic layer was separated and the aqueous layer extracted with ethyl acetate. The combined organic layers were dried with Na_2SO_4, filtered and the solvent was removed under reduced pressure to afford NMR spectroscopically pure **56 (**17 mg, 76%) as a colorless oil. $[\alpha]_D^{22}$ = +116.0 (c = 1.0, $CHCl_3$). ^{1}H NMR (500 MHz, $CDCl_3$): δ = 3.63, 3.86 (2 s, 3 H each, OMe), 5.89 (s, 1 H, 1'-H), 6.68 (s, 1 H, 5-H), 7.29-7.31, 7.43-7.44, 7.95-7.97 (3 m, 10 H, Ph) ppm. ^{13}C NMR (101 MHz, $CDCl_3$): δ = 56.4, 60.9 (2 q, OMe), 73.8 (d, C-1'), 104.4 (d, C-5), 127.2, 128.1, 128.5, 128.7, 129.4, 129.5 (6 d, Ph), 140.8, 142.5, 143.2, 148.4, 156.9, 161.7 (6 s, Ph, C-2, C-3, C-4, C-6) ppm. IR (neat): $\tilde{\nu}$ = 3020 (C-H), 2400-1550 (C=C) cm^{-1}. ESI-TOF: calc. for $[C_{20}H_{20}NO_3]^+$ 321.1365, found 321.1349.

(*S*)-2-(*tert*-Butyldimethylsiloxy)-*N*-(1-*tert*-butyl-2-methoxy-3-oxo-but-1-enyl)-propionamide (61)

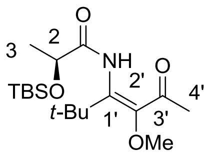

According to procedure 2, a mixture of methoxyallene (0.62 mL, 7.6 mmol), *n*-BuLi (2.5 M in hexanes, 2.85 mL, 7.13 mmol), pivalonitrile (189 mg, 2.38 mmol) and TBS-lactic acid (2.48 g, 11.9 mmol) in dry Et_2O (30 mL) provided 500 mg (58%) of enamide **61** as a pale yellow oil. ^{1}H NMR (400 MHz, $CDCl_3$): δ = 0.1, 0.91 (2 s, 6 H, 9 H, OTBS), 1.19 (s, 9 H, *t*-Bu), 1.32 (d, *J* = 6.7 Hz, 3 H, 3-H), 2.26 (s, 3 H, 4'-H), 3.50 (s, 3 H, OMe), 4.17 (q, *J* = 6.7 Hz, 1 H, 3-H), 7.78 (br s, 1 H, NH) ppm. ^{13}C NMR (101 MHz, $CDCl_3$): δ = -4.9, - 4.7 (2 q, OTBS), 17.9 (q, C-4'), 21.3 (q, C-3), 25.7, 27.2 (s, q, OTBS), 28.4, 36.3 (q, s, *t*-Bu), 59.0 (q, OMe), 70.3 (d, C-2), 129.8, 150.1 (2 s, C-1', C-2'), 172.7 (s, C-1), 200.1 (s, C-3') ppm. IR (ATR): $\tilde{\nu}$ = 3405 (NH), 2955-2860 (=CH, C-H), 1740-1700 (C=O), 1635-1585 (C=C) cm^{-1}. ESI-TOF: *m/z* calc. for $[C_{18}H_{36}NNaO_4Si]^+$ 380.2233, found 380.2245

(*S*)-2-(*tert*-Butyldimethylsiloxy)-*N*-(2-methoxy-3-oxo-1-phenylbut-1-enyl)-propionamide (62)

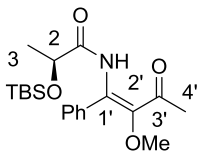

According to procedure 2, a mixture of methoxyallene (0.38 mL, 4.7 mmol), *n*-BuLi (2.5 M in hexanes, 1.74 mL, 4.35 mmol,), benzonitrile (150 µL, 1.40 mmol) and TBS-lactic

acid **60** (1.80 g, 8.61 mmol) in dry Et_2O (35 mL) gave 315 mg (58%) of enamide **62** as a pale yellow oil. ^{1}H NMR (400 MHz, $CDCl_3$): δ = 0.16, 0.19, 0.98 (3 s, 3 H, 3 H, 9 H, OTBS), 1.35 (d, J = 6.8 Hz, 3 H, 3-H), 2.33 (s, 3 H, 4'-H), 3.18 (s, 3 H, OMe), 4.19 (q, J = 6.8 Hz, 1 H, 2-H), 7.35-7.39 (m, 5 H, Ph), 11.52 (br s, 1 H, NH) ppm. ^{13}C NMR (101 MHz, $CDCl_3$): δ = - 4.8, - 4.7 (2 q, OTBS), 22.0 (q, C-3), 18.3, 25.7 (s, q, OTBS), 27.6 (q, C-4'), 60.5 (q, OMe), 70.6 (d, C-2), 127.9, 128.3, 128.7, 132.7, 139.5, 139.7 (3 s, 3 d, C-1', C-2', Ph), 174.0 (s, C-1), 200.7 (s, C-3') ppm. IR (ATR): $\tilde{\nu}$ = 3335 (NH), 3020-2855 (=CH, C-H), 1754-1570 (C=O, C=C) cm^{-1}. ESI-TOF: *m/z* calc. for $[C_{20}H_{31}NNaO_4Si]^+$ 400.1915, found 400.1921

(*S,E*)-Thiophene-2-carboxylic acid {1-[1-(*tert*-butyldimethylsiloxy)ethyl]-2-methoxy-3-oxo-but-1-enyl}amide (65)

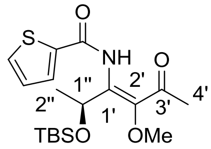

According to procedure 2, a mixture of methoxyallene (0.30 mL, 3.7 mmol), *n*-BuLi (2.5 M in hexanes, 1.30 mL, 3.25 mmol), TBS-lactic nitrile **63** (200 mg, 1.08 mmol) and thiophene-2 carboxylic acid (870 mg, 6.70 mmol) in dry Et_2O (75 mL) gave 302 mg (73%) of enamide **65** as a pale yellow oil. ^{1}H NMR (400 MHz, $CDCl_3$): δ = 0.07, 0.11, 0.89 (3 s, 3 H, 3 H, 9 H, OTBS), 1.46 (d, J = 6.5 Hz, 3 H, 2''-H), 2.31 (s, 3 H, 4'-H), 3.64 (s, 3 H, OMe), 5.34 (q, J = 6.5 Hz, 1 H, 1''-H), 7.08-7.10, 7.51-7.64 (2 m, 1 H, 2 H, Ar), 10.35 (br s, 1 H, NH) ppm. ^{13}C NMR (101 MHz, $CDCl_3$): δ = -4.8, -4.7 (2 q, OTBS), 21.9 (q, C-2''), 18.2, 25.9 (s, q, OTBS), 27.2 (q, C-4'), 61.1 (q, OMe), 65.4 (d, C-1''), 128.0, 129.5, 131.5, 139.3, 140.6, 141.5 (3 s, 3 d, C-1', C-2', Ar), 160.1 (s, C-1), 200.48 (s, C-

3') ppm. IR (ATR): $\tilde{\nu}$ = 3270 (NH), 3105-2860 (=CH, C-H), 1710-1470 (C=O, C=C) cm^{-1}. ESI-TOF: *m/z* calc. for $[C_{18}H_{29}NNaO_4SSi]^+$ 406.1484, found 406.1489

(*S*)-Pyridine-2-carboxylic acid {1-[1-(*tert*-butyldimethylsiloxy)ethyl]-2-methoxy-3 oxobut-1-enyl}amide (66)

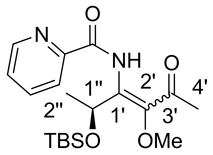

According to procedure 2, a mixture of methoxyallene (0.27 mL, 3.3 mmol), *n*-BuLi (2.5 M in hexanes, 1.16 mL, 2.90 mmol), TBS-lactic nitrile **63** (200 mg, 1.08 mmol) and picolinic acid (669 mg, 5.40 mmol) in dry Et_2O (75 mL) gave 210 mg (51%) of enamide **66** as a pale yellow oil. Enamide **66** was obtained as a 1:1 mixture of *E*- and *Z*-isomers. ^{1}H NMR (400 MHz, $CDCl_3$): δ = 0.04, 0.07, 0.10, 0.12, 0.94, 0.95 (6 s, 3 H, 3 H, 3 H, 3 H, 9 H, 9 H, OTBS), 1.28 (d, *J* = 6.2 Hz, 3 H, 2''-H), 1.29 (d, *J* = 6.5 Hz, 3 H, 2''-H), 2.30, 2.32 (2 s, 3 H each, 4'-H), 3.61, 3.62 (2 s, 3 H each, OMe), 5.07 (q, *J* = 6.5 Hz, 1 H, 1''-H), 5.17 (q, *J* = 6.2 Hz, 1 H each, 1''-H), 7.41-7.44, 7.80-7.86, 8.87-8.20, 8.53-8.57 (4 m, 1 H each, Ar), 8.22 (br s, 1H, NH) ppm. ^{13}C NMR (101 MHz, $CDCl_3$): δ = -5.1, -4.9, -4.72, -4.66 (4 q, OTBS), 18.07, 18.13 (2 s, OTBS), 22.2, 22.6 (2 q, C-2''), 25.73, 25.77 (2 q, OTBS), 27.6, 28.2 (2 q, C-4'), 58.8, 60.1 (2 q, OMe), 65.4, 66.1 (2 d, C-1''), 122.6, 122.7, 126.48, 126.54, 132.0, 134.4, 137.47, 134.50, 143.2, 143.8, 148.24, 148.27, 149.4, 149.6 (6 s, 8 d, C-1', C-2', Ar), 161.1, 162.4 (2 s, C-1), 198.1, 199.0 (2 s, C-3') ppm. IR (ATR): $\tilde{\nu}$ = 3325 (NH), 3060-2860 (=CH, C-H), 1755-1685 (C=O), 1590-1570 (C=C) cm^{-1}. ESI-TOF: *m/z* calc. for $[C_{19}H_{30}N_2NaO_4Si]^+$ 401.1873, found: 401.1889.

(*S*,*S*)-2-(*tert*-Butyldimethylsiloxy)-*N*-{1-[1-(*tert*-butyldimethylsiloxy)ethyl]-2-methoxy-3-oxo-but-1-enyl}propionamide (67)

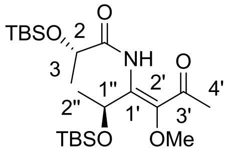

18

According to procedure 2, a mixture of methoxyallene (0.27 mL, 3.3 mmol), *n*-BuLi (2.5 M in hexanes, 1.25 mL, 3.13 mmol), TBS-lactic nitrile **63** (200 mg, 1.08 mmol) and TBS-lactic acid **60** (1.35 g, 6.40 mmol) in dry Et_2O (30 mL) gave 125 mg (25%) of enamide **67** as a pale yellow oil. ^{1}H NMR (500 MHz, $CDCl_3$): δ = 0.08, 0.09, 0.10, 0.12 (4 s, 6 H each, OTBS), 0.88, 0.91 (2 s, 9 H each, OTBS), 1.22 (d, *J* = 6.3 Hz, 3 H, 3-H or 2''-H), 1.35 (d, *J* = 6.7 Hz, 3 H, 3-H or 2''-H), 2.23 (s, 3 H, 4'-H), 3.57 (s, 3 H, OMe), 4.25 (q, *J* = 6.7 Hz, 1 H, 2-H or 1''-H), 5.00 (q, *J* = 6.3 Hz, 1 H, 2-H or 1''-H), 8.61 (br s, 1 H, NH) ppm. ^{13}C NMR (125 MHz, $CDCl_3$): δ = -5.6, -5.5, -5.4, -5.3 (4 q, OTBS), 17.5, 17.6 (2 s, OTBS), 21.0, 21.2 (2 q, C-3/C-2''), 24.7, 25.2 (2 q, OTBS), 26.7 (q, C-4'), 59.3 (q, OMe), 64.3, 69.9 (2 d, C-2/C-1''), 128.8, 142.9 (2 s, C-1', C-2'), 171.9 (s, C-1), 197.1 (s, C-3') ppm. IR (ATR): $\tilde{\nu}$ = 3415 (NH), 2950-2860 (=CH, C-H), 1725 (C=O), 1570-1460 (C=C) cm^{-1}. ESI-TOF: *m/z* calc. for $[C_{22}H_{45}NNaO_5Si_2]^+$ 482.2734, found 482.2740.

(*S*)-2-*tert*-Butyl-6-[1-(*tert*-butyldimethylsiloxy)ethyl]-3-methoxypyridin-4-yl nonaflate (68)

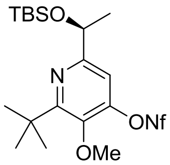

According to procedure 3, a mixture of enamide **61** (100 mg, 0.280 mmol), triethylamine (0.12 mL, 0.84 mmol), TMSOTf (0.15 mL, 0.84 mmol) in 1,2-dichloroethane (4 mL) gave 74 mg (78%) of the corresponding pyridine derivative as a brown oil. A mixture of the pyridine derivative (355 mg, 1.05 mmol), NaH (120 mg, 3.14 mmol), and NfF (0.58 mL, 3.14 mmol) in THF (10 mL) gave 468 mg (72%) of **68** as a colorless oil. $[\alpha]_D^{22}$ = +13.3 (c = 0.60, $CHCl_3$). ^{1}H NMR (400 MHz, $CDCl_3$): δ = 0.04, 0.10, 0.93 (3 s, 3 H, 3 H, 9 H, OTBS), 1.39 (s, 9 H, *t*-Bu), 1.45 (d, *J* = 6.5 Hz, 3 H, 2'-H), 3.90 (s, 3 H, OMe), 4.86 (q, *J* = 6.5 Hz, 1 H, 1'-H), 7.33 (s, 1 H, 5-H) ppm. ^{13}C NMR (125 MHz, $CDCl_3$): δ = - 5.1, - 4.6 (2 q, OTBS), 18.0, 24.6 (s, q, OTBS), 29.3 (q, C-2'), 25.7, 38.6 (q, s, *t*-Bu), 61.6 (q, OMe), 71.3 (d, C-1'), 145.3, 150.3, 151.8, 160.6, 163.2 (1 d, 4 s, C-2, C-3, C-4, C-5, C-6) ppm. IR (ATR): $\tilde{\nu}$ = 2975-2870 (C-H), 1690-1515 (C=C) cm^{-1}. ESI-TOF: *m/z* calcd. for $[C_{22}H_{32}F_9NO_5SSiNa]^+$: 644.1524; found: 644.1516.

(*S*)-6-[1-(*tert*-Butyldimethylsiloxy)ethyl]-3-methoxy-2-phenylpyridin-4-yl nonaflate (69)

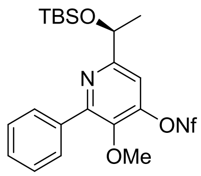

According to procedure 3, a mixture of enamide **62** (112 mg, 0.297 mmol), triethylamine (0.12 mL, 0.89 mmol), TMSOTf (0.16 mL, 0.88 mmol) in 1,2-dichloroethane (3 mL) gave 93 mg (87%) of the corresponding pyridine derivative as a brown oil. A mixture of the pyridine derivative (129 mg, 0.36 mmol), NaH (40 mg, 1.00 mmol), and NfF (184 µL, 1.03 mmol) in THF (3 mL) gave 161 mg (70%) of **69** as a colorless oil. $[\alpha]_D^{22}$= +6.19 (c = 0.35, $CHCl_3$). ^1H NMR (500 MHz, $CDCl_3$): δ = 0.05, 0.10, 0.94 (3 s, 3 H, 3 H, 9 H, OTBS), 1.49 (d, *J* = 6.5 Hz, 3 H, 2'-H), 3.62 (s, 3 H, OMe), 4.95 (q, *J* = 6.5 Hz, 1 H, 1'-H), 7.14 (s, 1 H, 5-H), 7.34-7.43 (m, 3 H, Ph), 7.85-7.87 (m, 2 H, Ph) ppm. ^{13}C NMR (125 MHz, $CDCl_3$): δ = - 4.8, - 4.5 (2 q, OTBS), 18.3 (q, C-2'), 25.5, 25.9 (q, s, OTBS), 60.6 (q, OMe), 72.1 (d, C-1'), 102.0, 128.1, 128.2, 129.2, 137.9, 142.0, 150.4, 159.7, 162.3 (4 d, 5 s, C-2, C-3, C-4, C-5, C-6, Ph) ppm. IR (ATR): $\tilde{\nu}$ = 2960-2870 (C-H), 1660-1400 (C=C) cm^{-1}.

(*S*)-2-[1-(*tert*-Butyldimethylsiloxy)-ethyl]-3-methoxy-6-(thiophen-2-yl)pyridin-4-yl nonaflate (71)

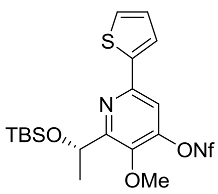

According to procedure 3, a mixture of enamide **65** (125 mg, 0.30 mmol), triethylamine (0.14 mL, 1.0 mmol), TMSOTf (0.18 mL, 0.97 mmol) in 1,2-dichloroethane (3 mL) gave 78 mg (65%) of the corresponding pyridine derivative as a brown oil. A mixture of the pyridine derivative (58 mg, 0.16 mmol), NaH (16 mg, 0.39 mmol), and NfF (0.08 mL, 0.390 mmol) in THF (3 mL) gave 82 mg (80%) of **71** as a colorless oil. $[\alpha]_D^{22}$= -11.9 (c = 3.7, $CHCl_3$). ^{1}H NMR (500 MHz, $CDCl_3$): δ = 0.01, 0.04, 0.87 (3 s, 3 H, 3 H, 9 H, OTBS), 1.58 (d, *J* = 6.5 Hz, 3 H, 2'-H), 3.93 (s, 3 H, OMe), 5.23 (q, *J* = 6.5 Hz, 1 H, 1'-H), 7.08-7.10 (m, 1 H, 4-H_{thio}), 7.40 (m, 2 H, 3-H_{thio}, 5-H), 7.51-7.52 (dd, *J* = 3.7, 1.0 Hz, 1 H, 5-H_{thio}) ppm. ^{13}C NMR (125 MHz, $CDCl_3$): δ = -4.6, -4.3 (2 q, OTBS), 18.3, 22.6 (s, q, OTBS), 25.9 (q, C-2'), 62.7 (q, OMe), 68.7 (d, C-1'), 110.7, 125.1, 128.1, 128.6, 143.4, 144.1, 149.0, 150.0, 160.1 (4 d, 5 s, C-2, C-3, C-4, C-5, C-6, Thiophenyl) ppm. IR (ATR): $\tilde{\nu}$ = 2960-2850 (=CH, C-H), 1590-1430 (C=C) cm^{-1}. ESI-TOF: *m/z* calc. for $[C_{22}H_{26}F_9NO_5S_2SiNa]^+$: 670.0776; found: 670.0794.

(*S*)-6-[1-(*tert*-Butyldimethylsiloxy)-ethyl]-5-methoxy-[2,2']bipyridin-4-yl nonaflate (72)

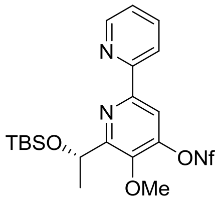

According to procedure 3, a mixture of enamide **66** (100 mg, 0.264 mmol), triethylamine (0.12 mL, 0.85 mmol), TMSOTf (0.14 mL, 0.80 mmol) in 1,2-dichloroethane (5 mL) gave 80 mg (84%) of the corresponding pyridine derivative as a brown oil. A mixture of the pyridine derivative (38 mg, 0.11 mmol), NaH (13 mg, 0.33 mmol), and NfF (0.06 mL, 0.31 mmol) in THF (6 mL) gave 57 mg (86%) of **72** as a colorless oil. $[\alpha]_D^{22}$ = -2.0 (c = 0.6, $CHCl_3$). ^{1}H NMR (500 MHz, $CDCl_3$): δ = - 0.01, 0.04, 0.87 (3 s, 3 H, 3 H, 9 H, OTBS), 1.59 (d, *J* = 6.6 Hz, 3 H, 2'-H), 3.96 (s, 3 H, OMe), 5.32 (q, *J* = 6.6 Hz, 1 H, 1'-H), 7.29-7.31, 7.78-7.82 (2 m, 1 H each, Pyr), 8.29 (s, 1 H, 5-H), 8.47 (d, *J* = 8.0 Hz, 1 H, Pyr), 8.64-8.65 (m, 1 H, Pyr) ppm. ^{13}C NMR (125 MHz, $CDCl_3$): δ = - 4.5, - 4.7 (2 q, OTBS), 18.3, 22.7 (s, q, OTBS), 25.9 (q, C-2'), 62.6 (q, OMe), 68.2 (d, C-1'), 113.4, 121.2, 124.1, 137.0, 145.6, 149.2, 150.3, 152.8, 154.4, 159.5 (5 d, 5 s, C-2, C-3, C-4, C-5, C-6, Pyr) ppm. IR (ATR): $\tilde{\nu}$ = 2965-2870 (=CH, C-H), 1600-1435 (C=C) cm^{-1}. ESI-TOF: calc. for $[C_{23}H_{28}F_9N_2O_5SSi]^+$ 643.1339, found 643.1346.

(*S*,*S*)-2,6-Bis-[1-(*tert*-butyl-dimethylsilyloxy) ethyl]-3-methoxypyridin-4-yl nonaflate (73)

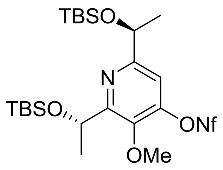

According to procedure 3, a mixture of enamide **67** (50 mg, 0.11 mmol), triethylamine (0.05 mL, 0.35 mmol), TMSOTf (0.06 mL, 0.32 mmol) in 1,2-dichloroethane (3 mL) gave 37 mg (77%) of the corresponding pyridine derivative as a brown oil. A mixture of the pyridine derivative (43 mg, 0.10 mmol), NaH (16 mg, 0.39 mmol), and NfF (0.07 mL, 0.39 mmol) in THF (3 mL) gave 35 mg (50%) of **73** as a colorless oil. ^{1}H NMR (500 MHz, $CDCl_3$): δ = 0.02, 0.01, 0.00 (3 s, 3 H, 3 H, 6 H, OTBS), 0.83, 0.90 (2 s, 9 H each, OTBS), 1.40 (d, *J* = 6.3 Hz, 3 H, 2'-H or 2''-H), 1.48 (d, *J* = 6.5 Hz, 3 H, 2'-H or 2''-H), 3.82 (s, 3 H, OMe), 4.90 (q, *J* = 6.5 Hz, 1 H, 1'-H or 1''-H), 5.21 (q, *J* = 6.3 Hz, 1 H, 1'-H or 1''-H), 7.03 (s, 1 H, 5-H) ppm. ^{13}C NMR (125 MHz, $CDCl_3$): δ = - 4.8, - 4.72, - 4.71, - 4.5 (4 q, OTBS), 18.3, 18.5, 25.9, 26.0 (2 s, 2 q, OTBS), 23.3, 26.1 (2 q, C-2'/C-2'') 61.1 (q, OMe), 68.1, 72.2 (2 d, C-1'/C-1''), 140.8, 155.5, 158.4, 159.0, 162.0 (d, 4 s, C-2, C-3, C-4, C-5, C-6) ppm. (ATR): $\tilde{\nu}$ = 2965-2870 (=CH, C-H), 1600-1435 (C=C) cm^{-1}. ESI-TOF: calc. for $[C_{26}H_{41}F_9NNaO_6SSi_2]^+$ 746.2020, found 746.2040.
