# Supplementary material for: A practical two-step procedure for the preparation of enantiopure pyridines: Multicomponent reactions of alkoxyallenes, nitriles and carboxylic acids followed by a cyclocondensation reaction
Source: Beilstein J Org Chem. 2011 Jul 13;7:962–75. doi: 10.3762/bjoc.7.108 (PMC3170199; doi:10.3762/bjoc.7.108)

Supporting Information

for

A practical two-step procedure for the preparation of enantiopure pyridines: Multicomponent reactions of alkoxyallenes, nitriles and carboxylic acids followed by a cyclocondensation reaction

Christian Eidamshaus, Roopender Kumar, Mrinal K. Bera and Hans-Ulrich Reissig*

Address: Freie Universität Berlin, Institut für Chemie und Biochemie, Takustr. 3, D-14195 Berlin, Germany

Email: Hans-Ulrich Reissig* - hreissig@chemie.fu-berlin.de

* Corresponding author

^{1}H and ^{13}C NMR spectra of synthesized compounds

^{13}C NMR spectra recorded at 101 MHz show signals at 27.5, 103.5 and 179.1 ppm, which are caused by external electromagnetic interference. Due to the pyridinol–pyridone tautomerization the signals in some spectra are broadened.

(*S*)-6-*sec*-Butyl-2-*tert*-butyl-3-methoxypyridin-4-one (**18)**

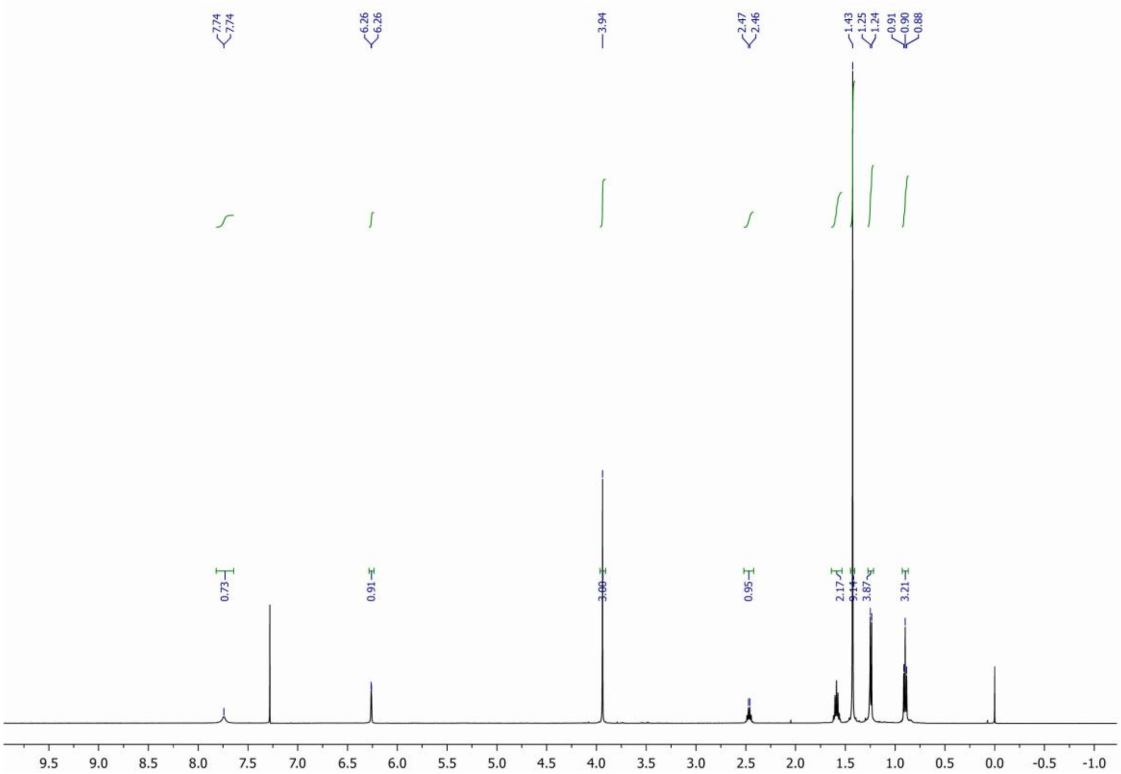

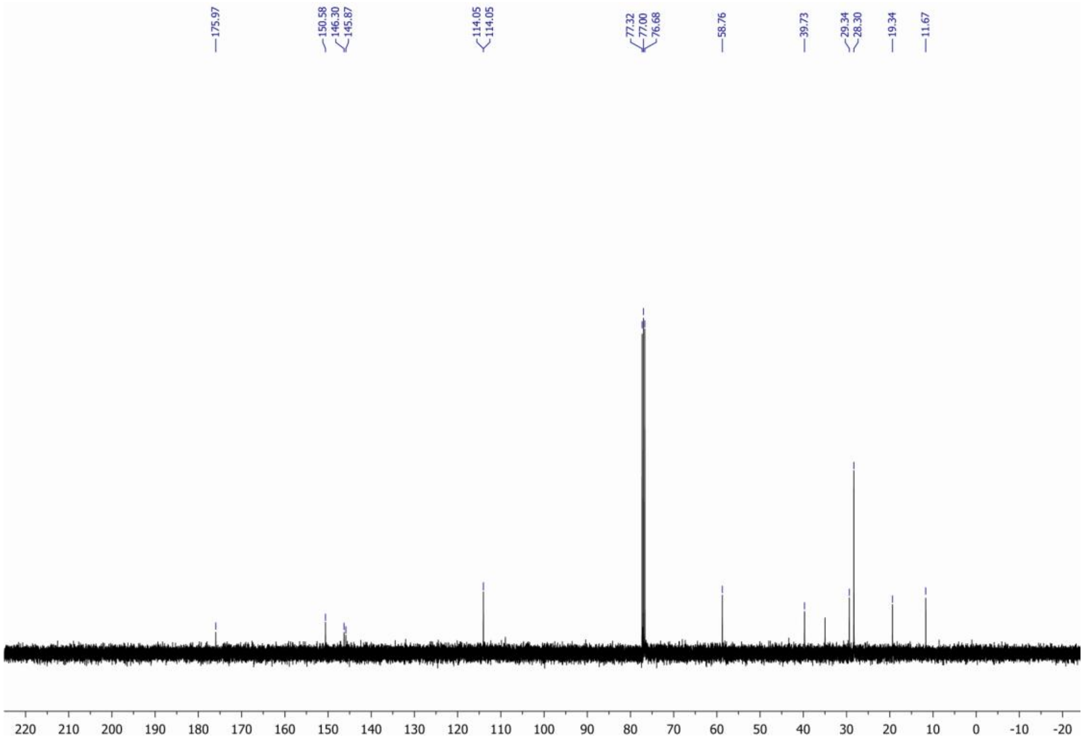

(S)-2-*tert*-Butyl-3-methoxy-6-(2,2,2-trifluoro-1-methoxy-1-phenyl ethyl)pyridin-4-yl nonaflate (**52**)

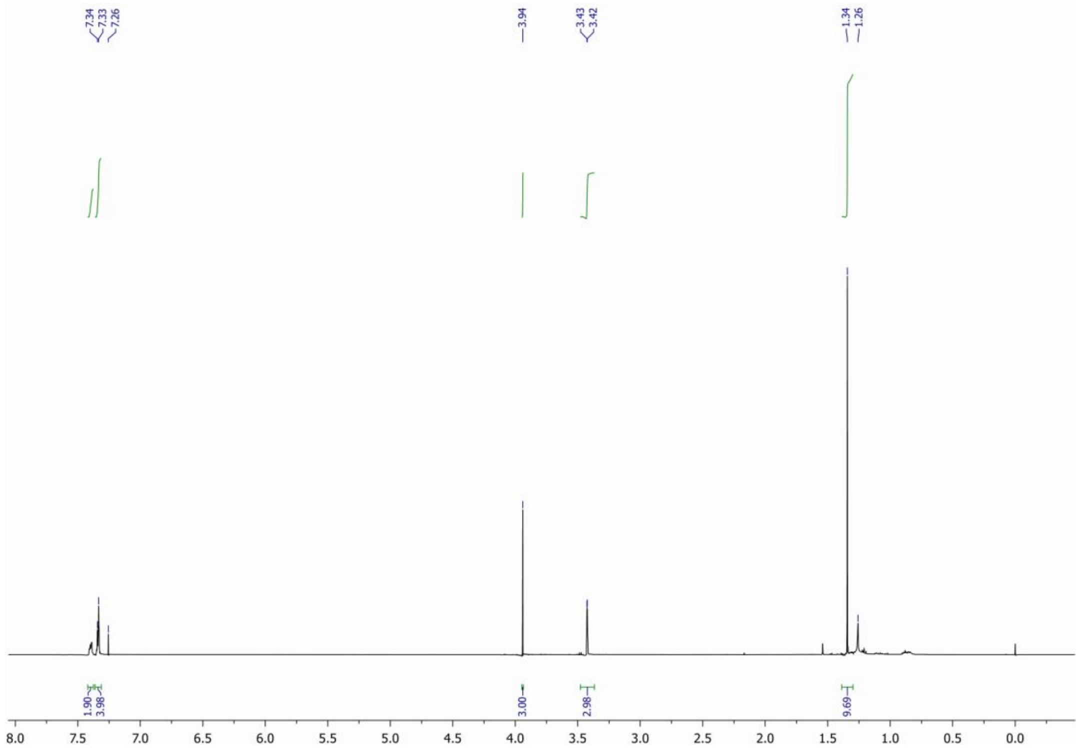

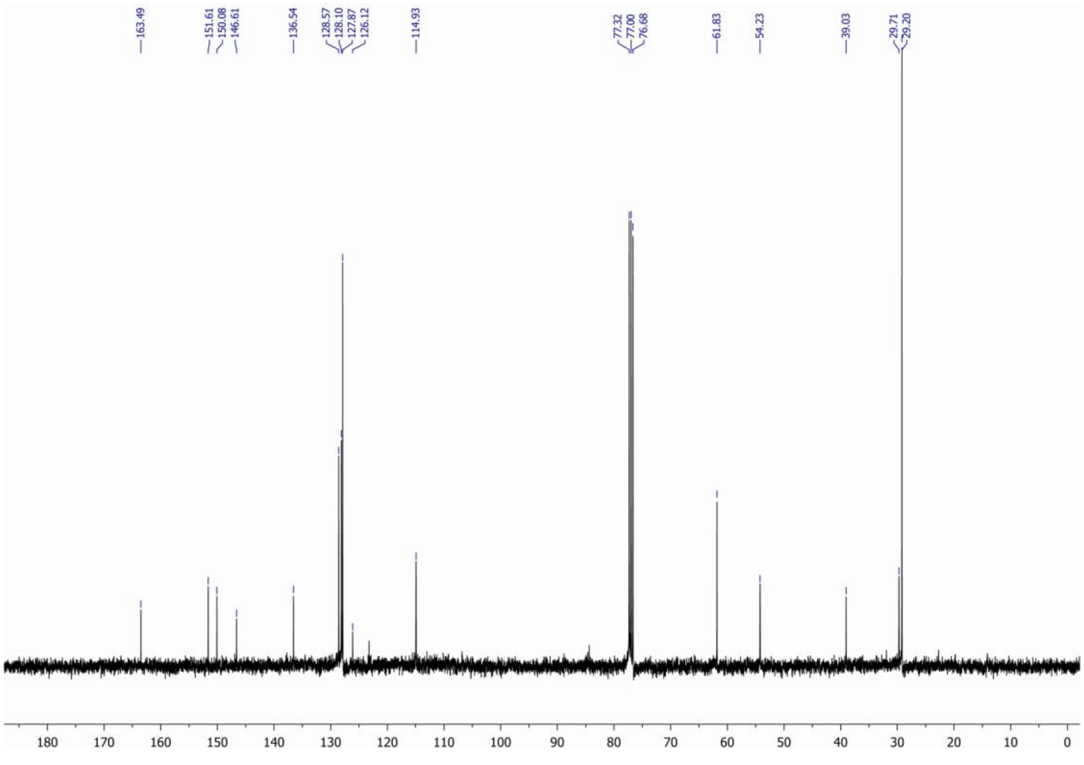

(*S*)-2-*tert*-Butyl-6-[(*tert*-butyldimethylsiloxy)phenylmethyl]-3-methoxypyridin-4-one (**22**)

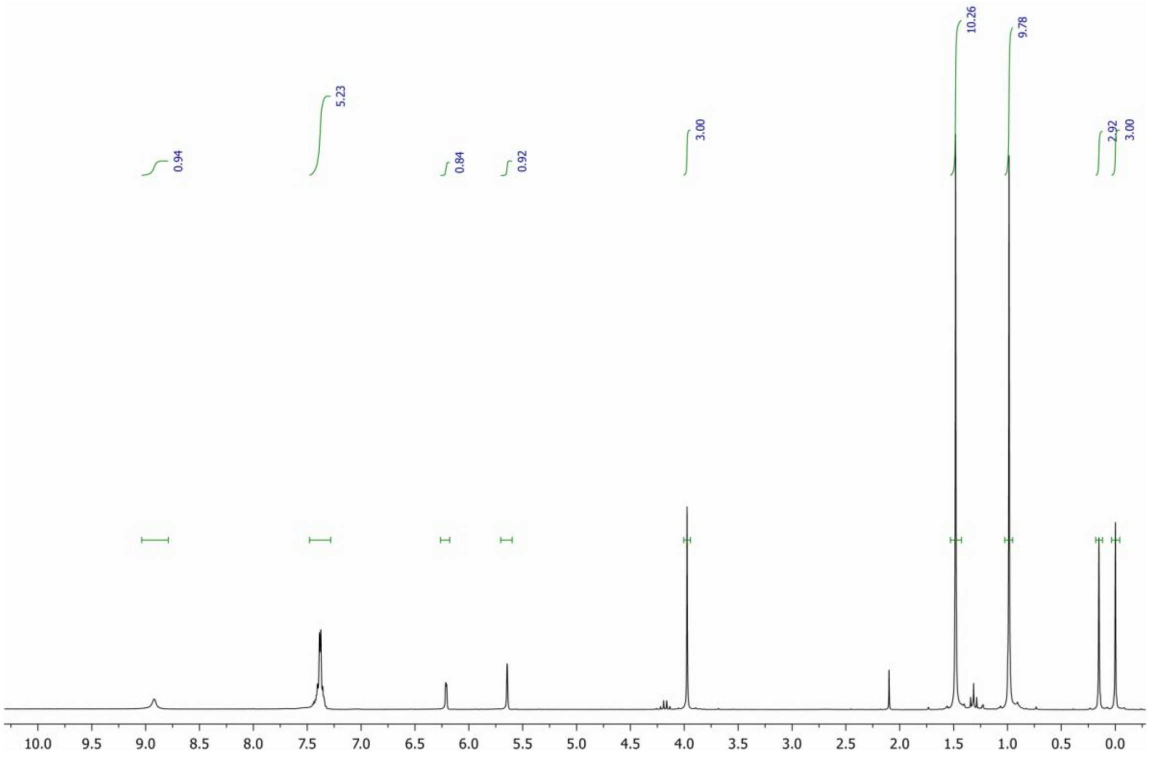

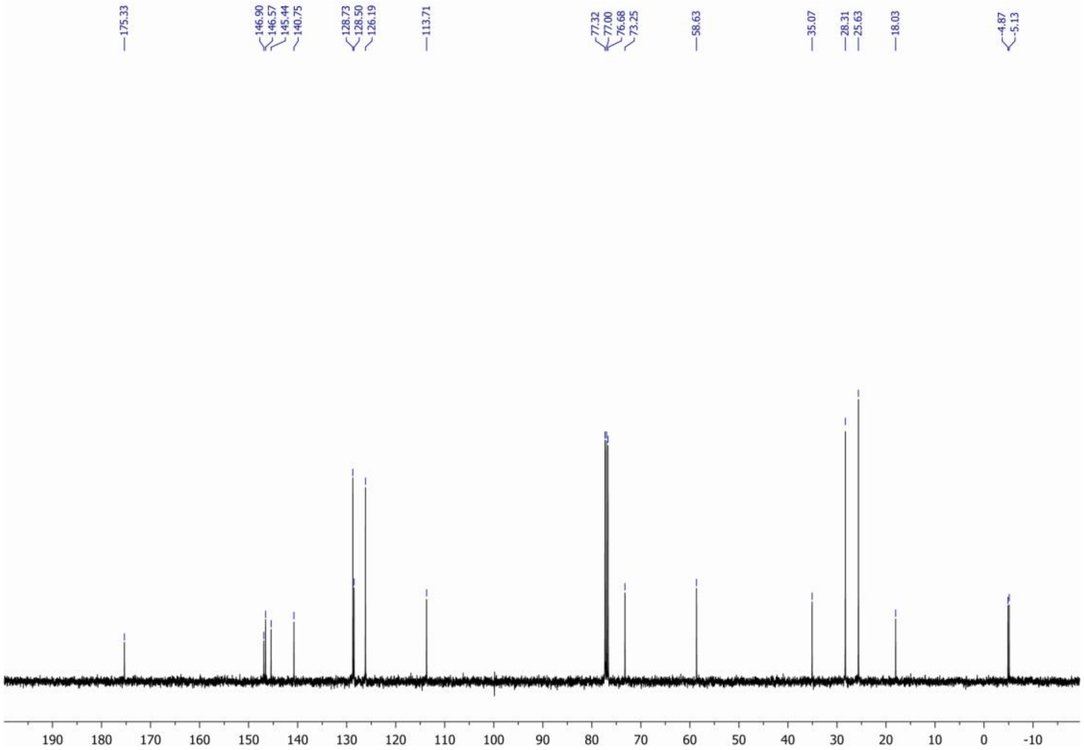

(*S*)-2-*tert*-Butyl-6-(1,1-dibenzylamino-2-phenylethyl)-3-methoxypyridin-4-one (**24**)

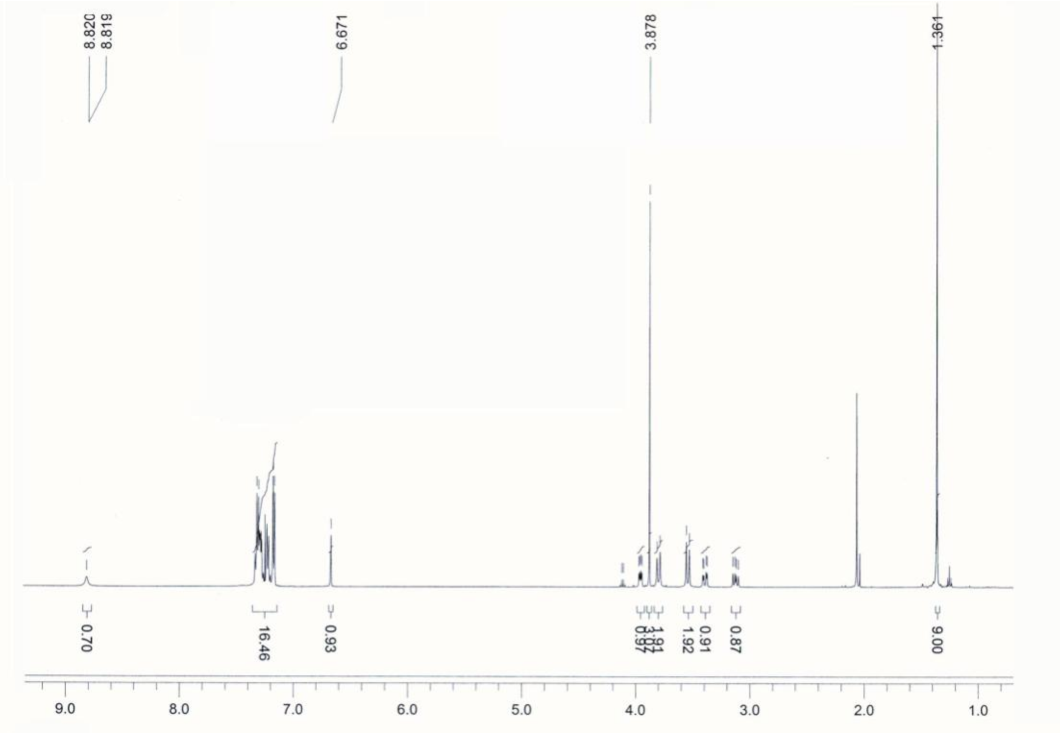

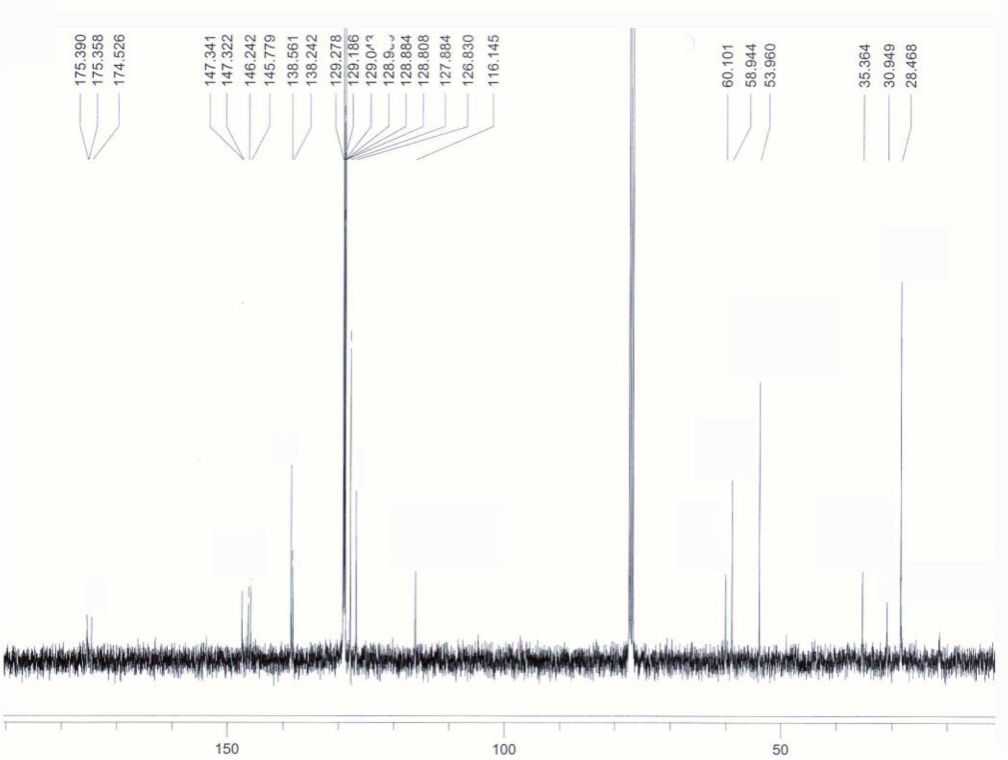

rac-2-*tert*-Butyl-6-(1-dibenzylamino-2-methylpropyl)-3-methoxy-pyridin-4-one (**26**)

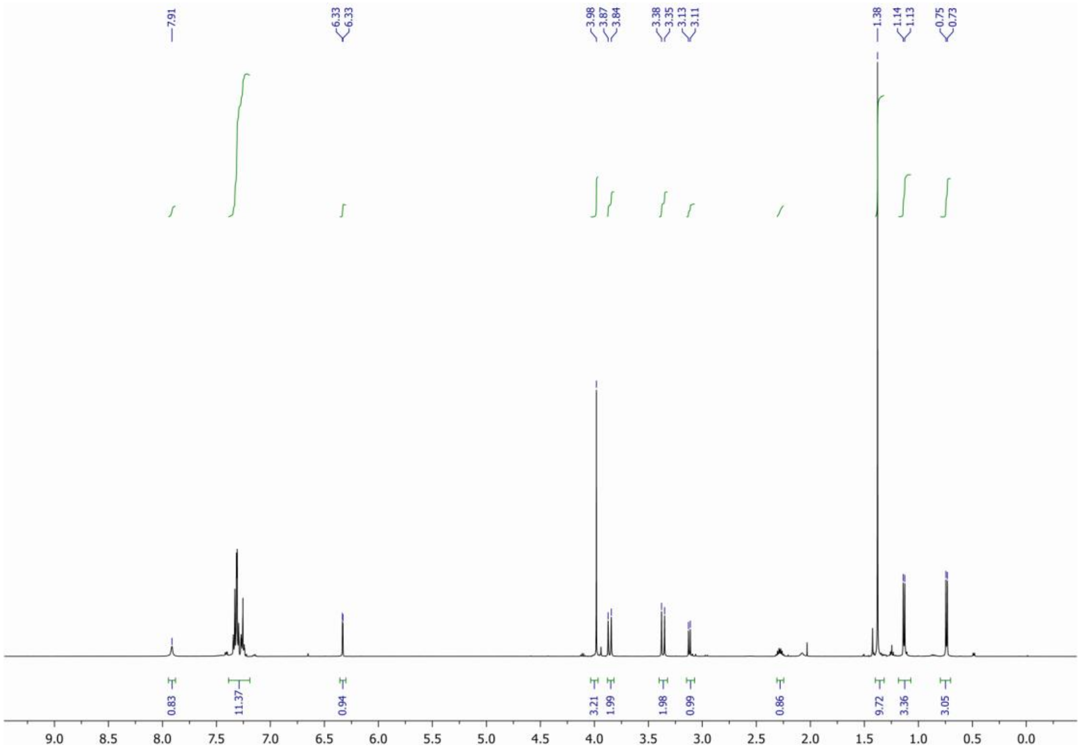

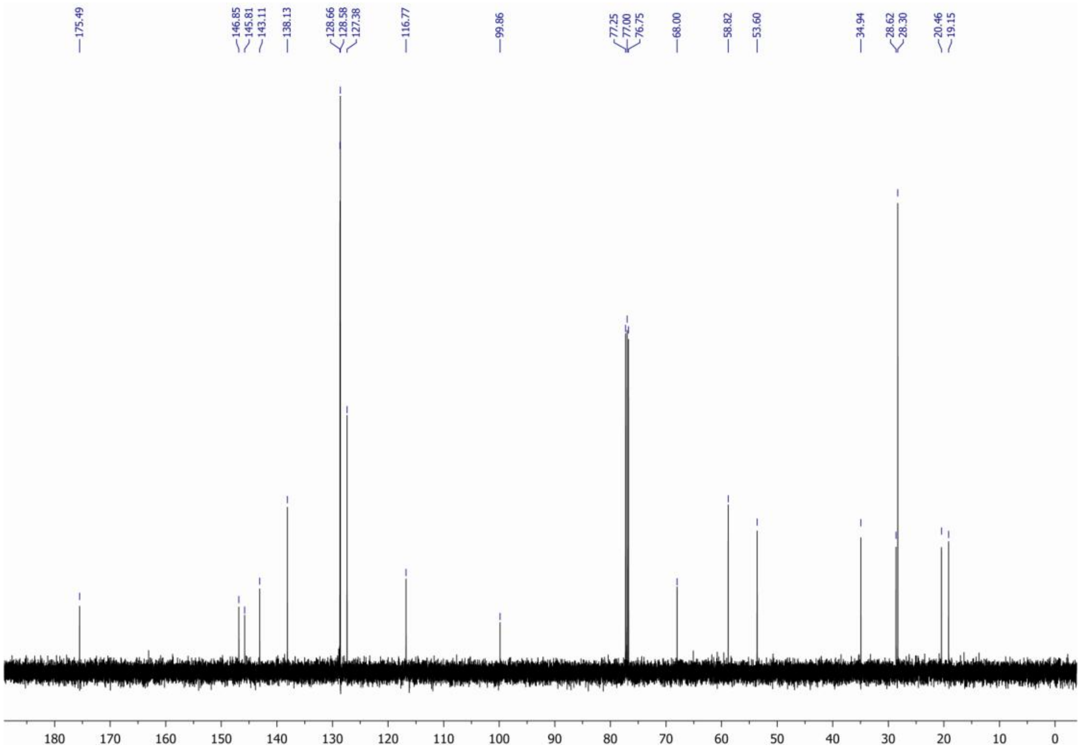

(*R*)-2-*tert*-Butyl-3-methoxy-6-(1-phenylpropyl)pyridin-4-ol (**28**)

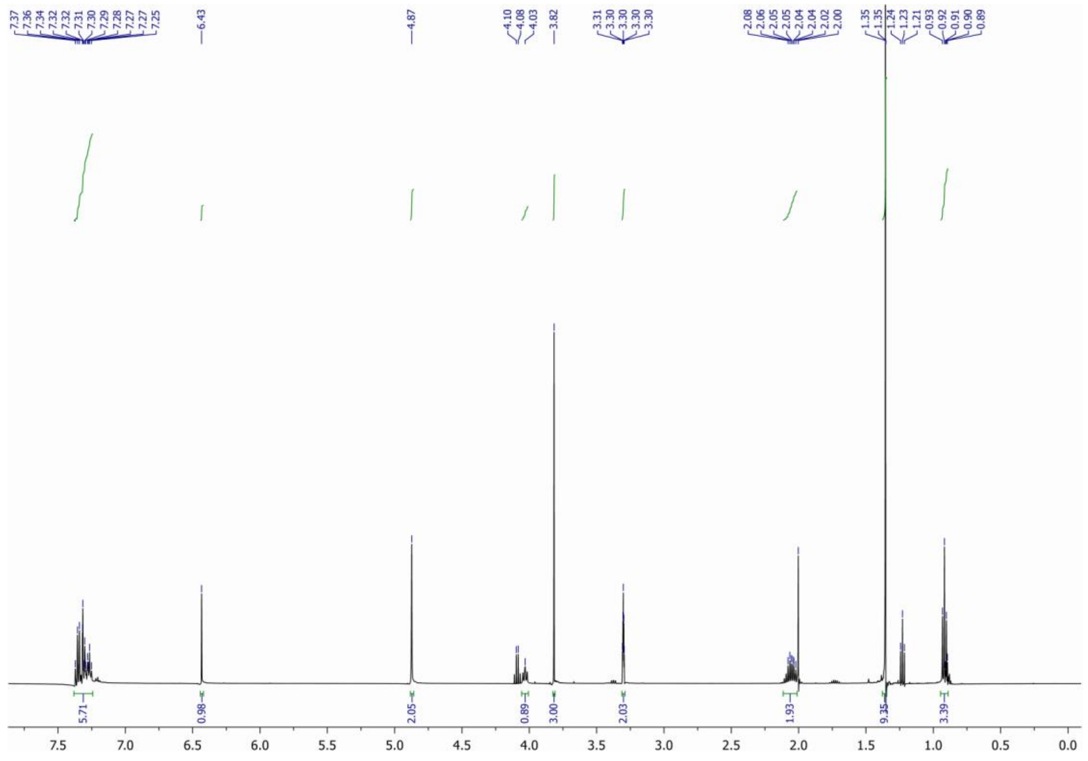

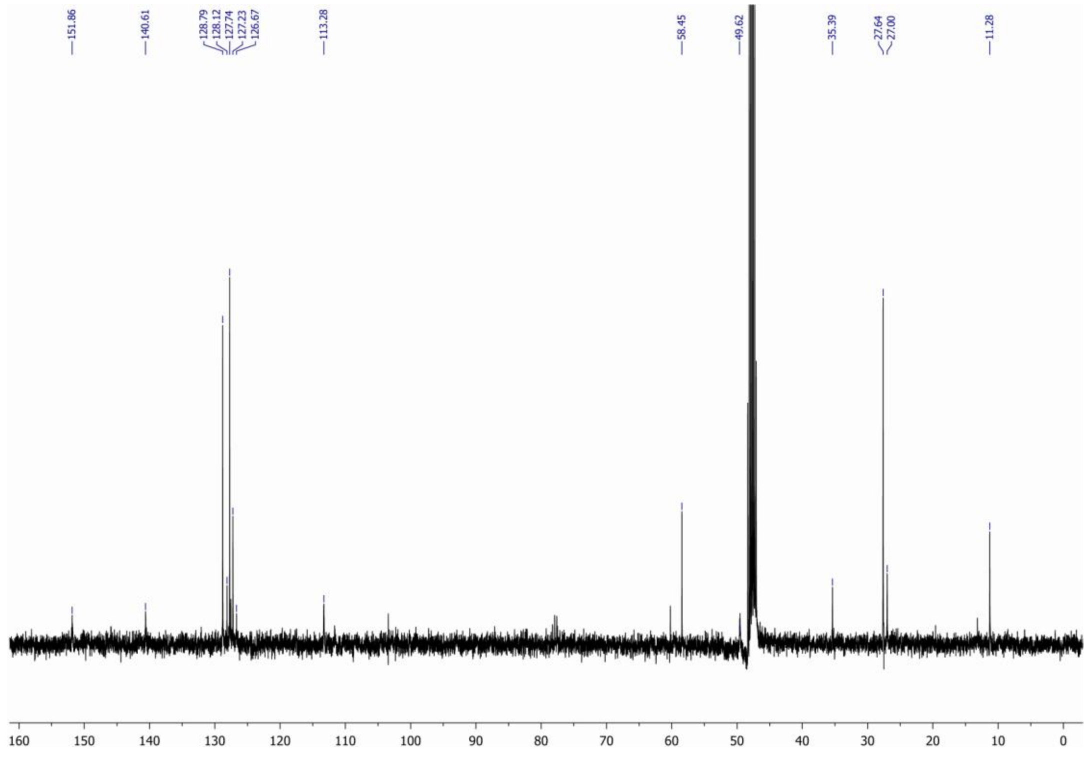

(*S*,*S*)-2,6-Di-*sec*-butyl-3-methoxypyridin-4-one (**32**)

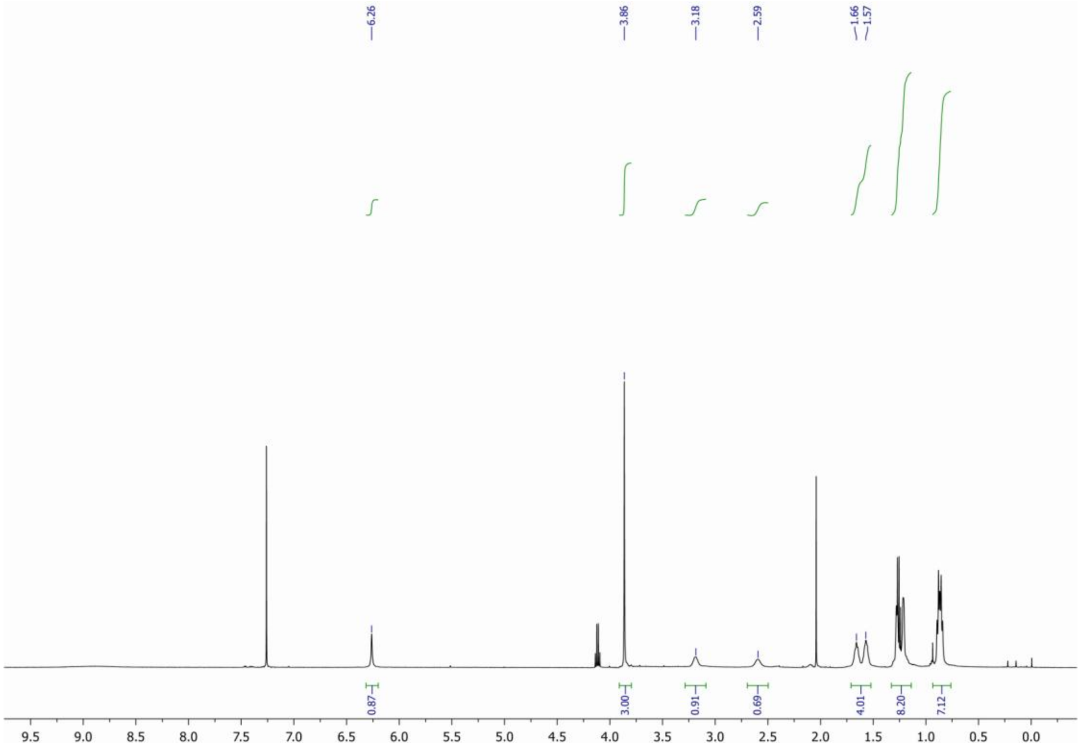

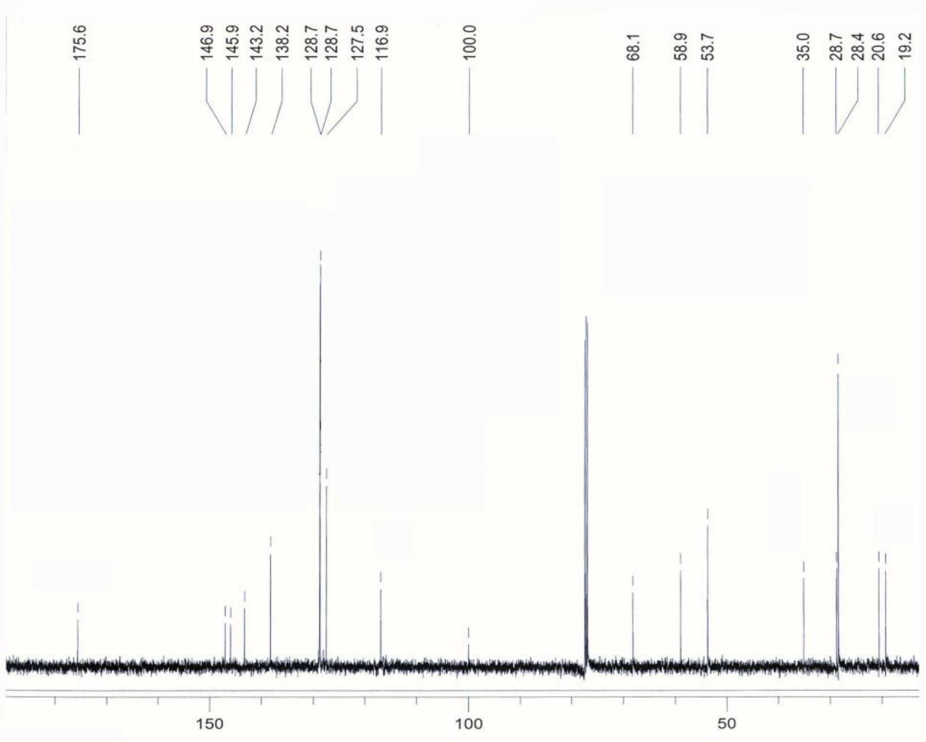

2,6-bis{(*R*,*R*)-[*tert*-Butyldimethylsiloxy)(phenyl)methyl]-3-methoxypyridin-4(1*H*)-one (**35**)

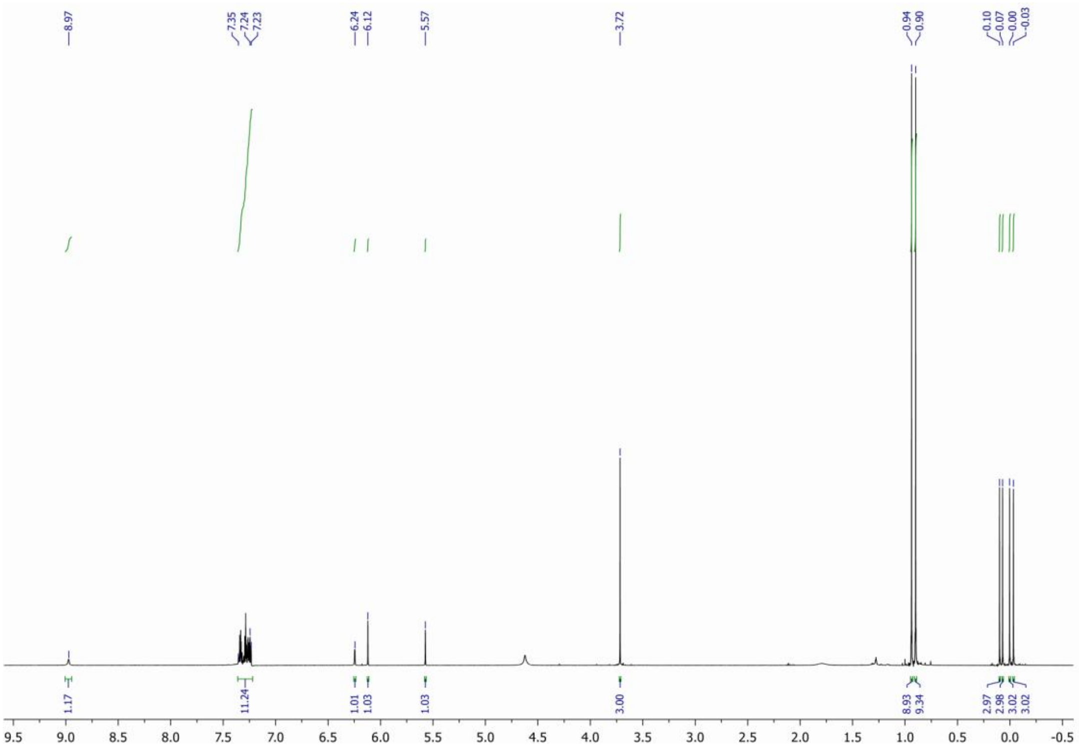

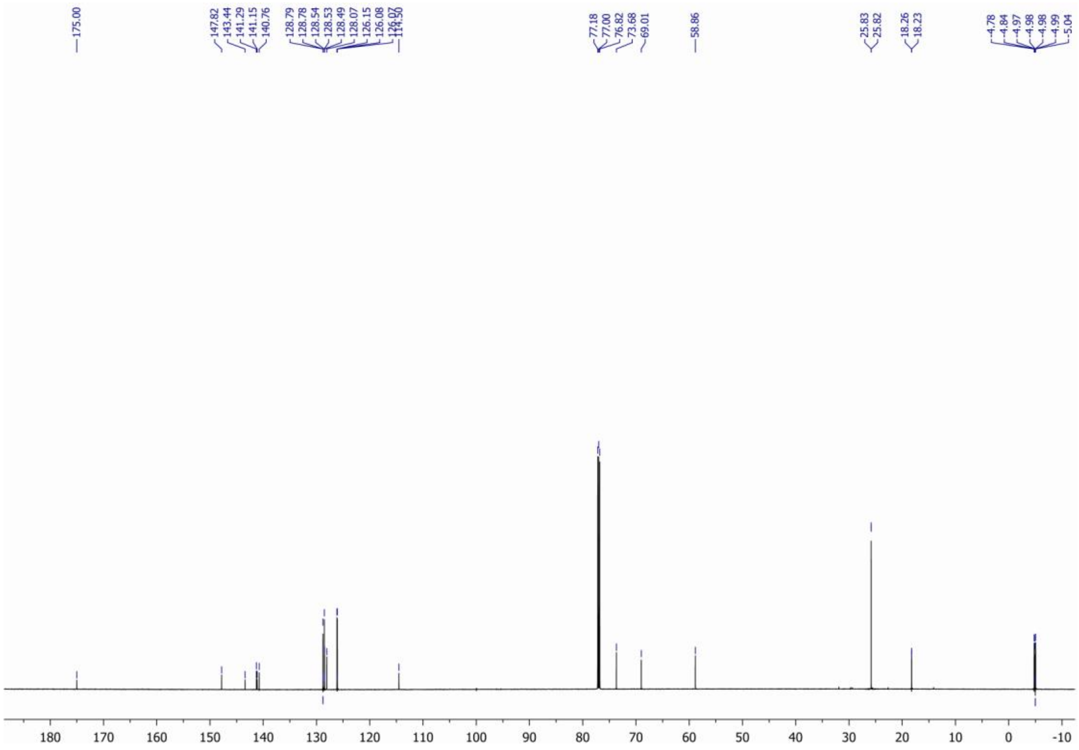

6-{(*R*)-[*tert*-Butyldimethylsiloxy)(phenyl]methyl}-2-{(*S*)-[*tert*-butyldimethylsilyloxy](phenyl)methyl}-3-methoxypyridin-4(1*H*)-one (**34**)

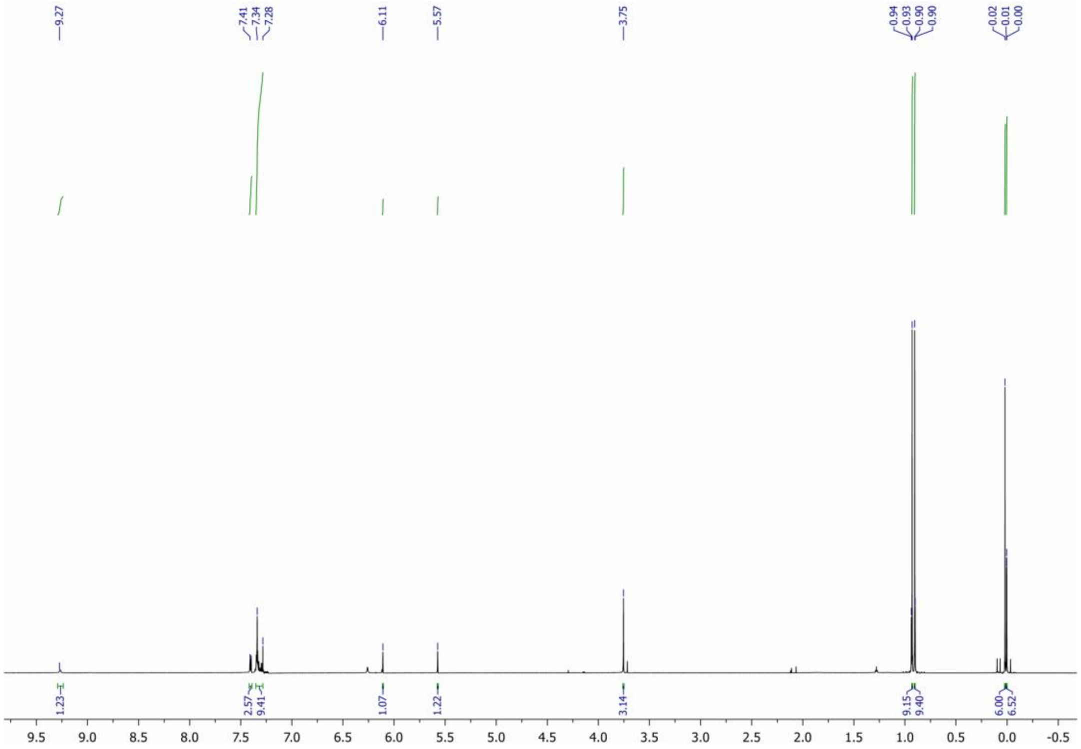

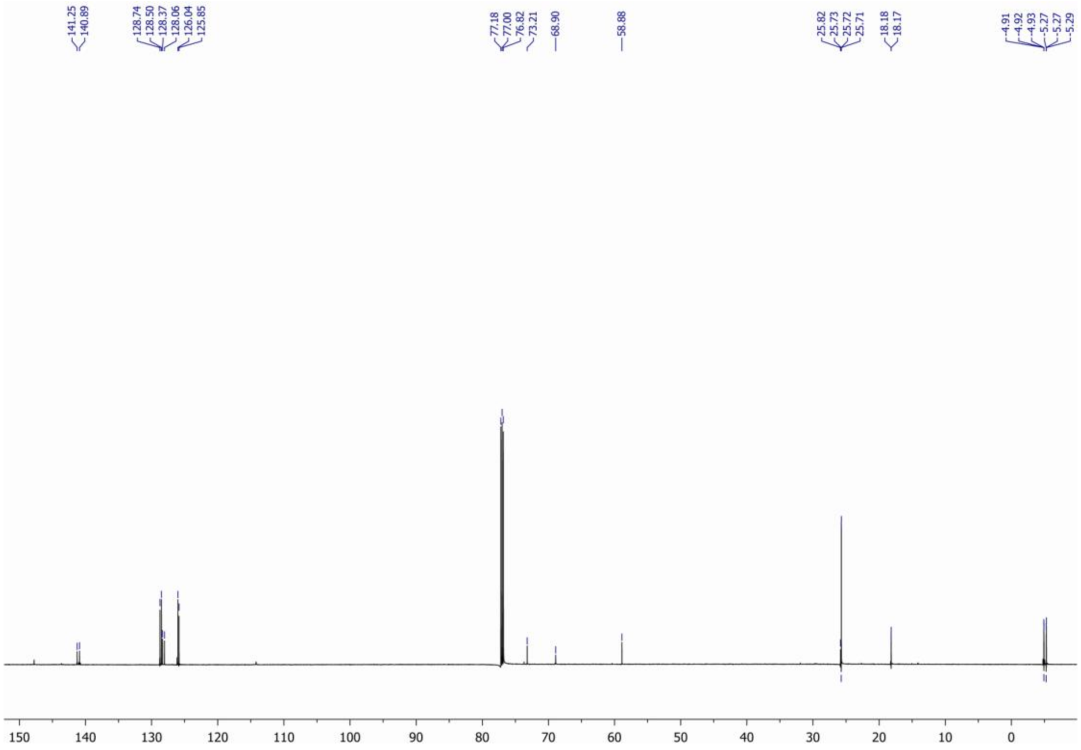

(*R*)-2-[(*tert*-Butyldimethylsiloxy)phenylmethyl]-3-methoxy-6-(trifluoromethyl)pyridin-4-one (**37**)

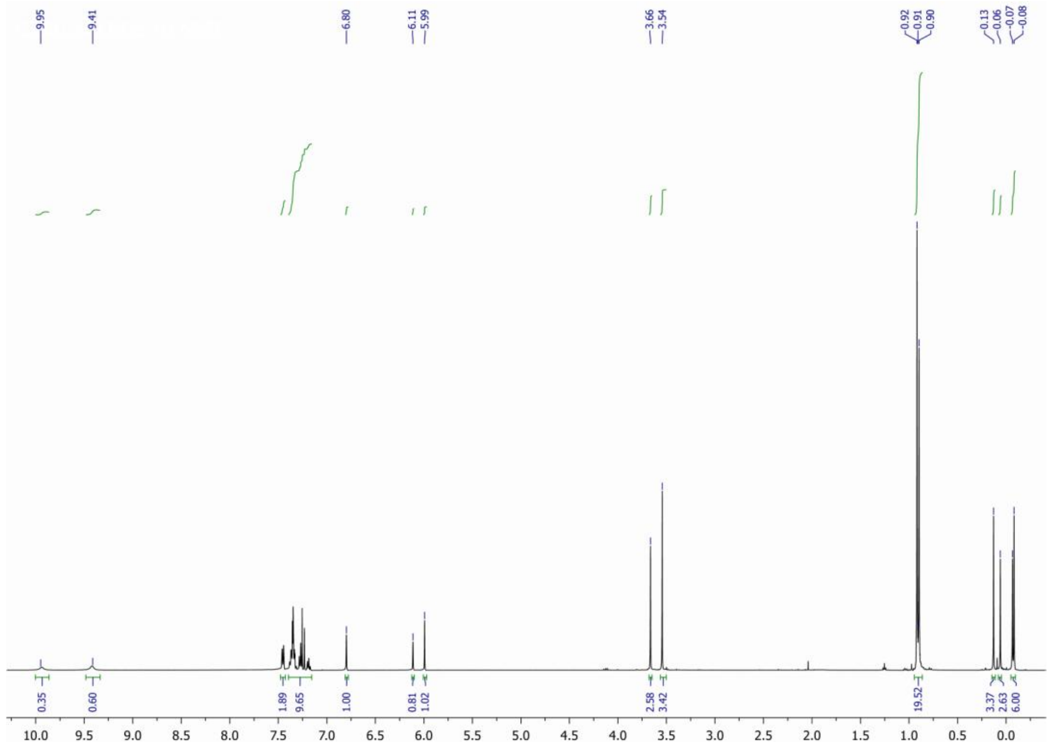

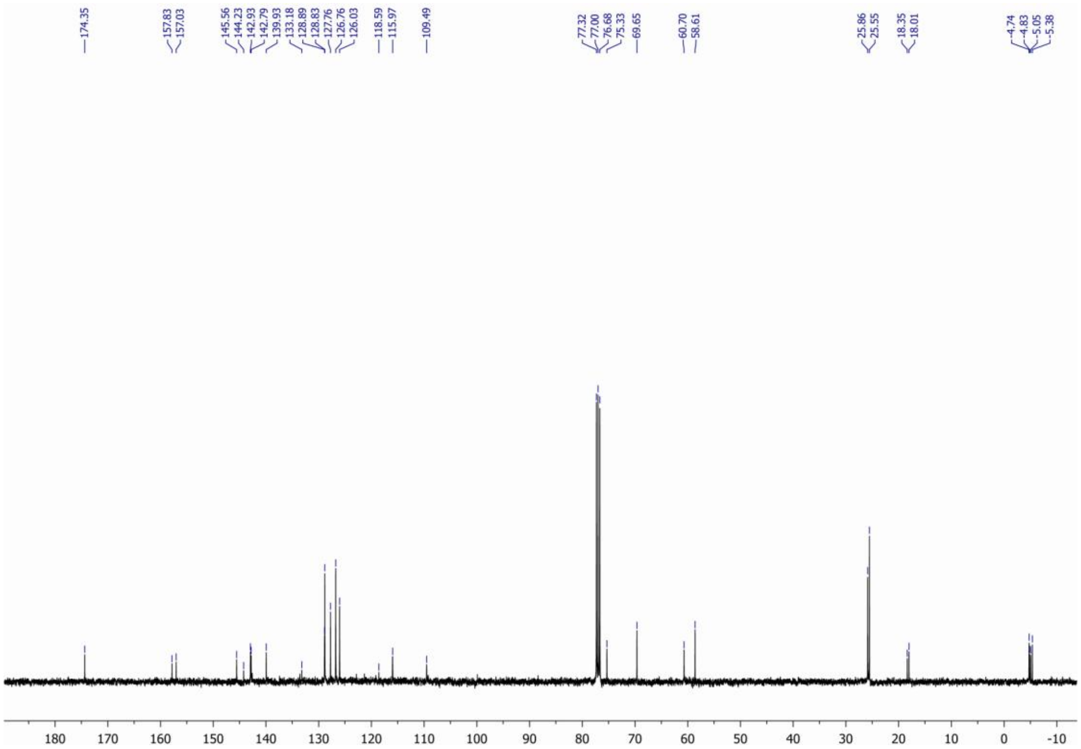

(*S*)-2-(2-Phenylpropyl)-6-(trifluoromethyl)pyridin-4-ol (**39**)

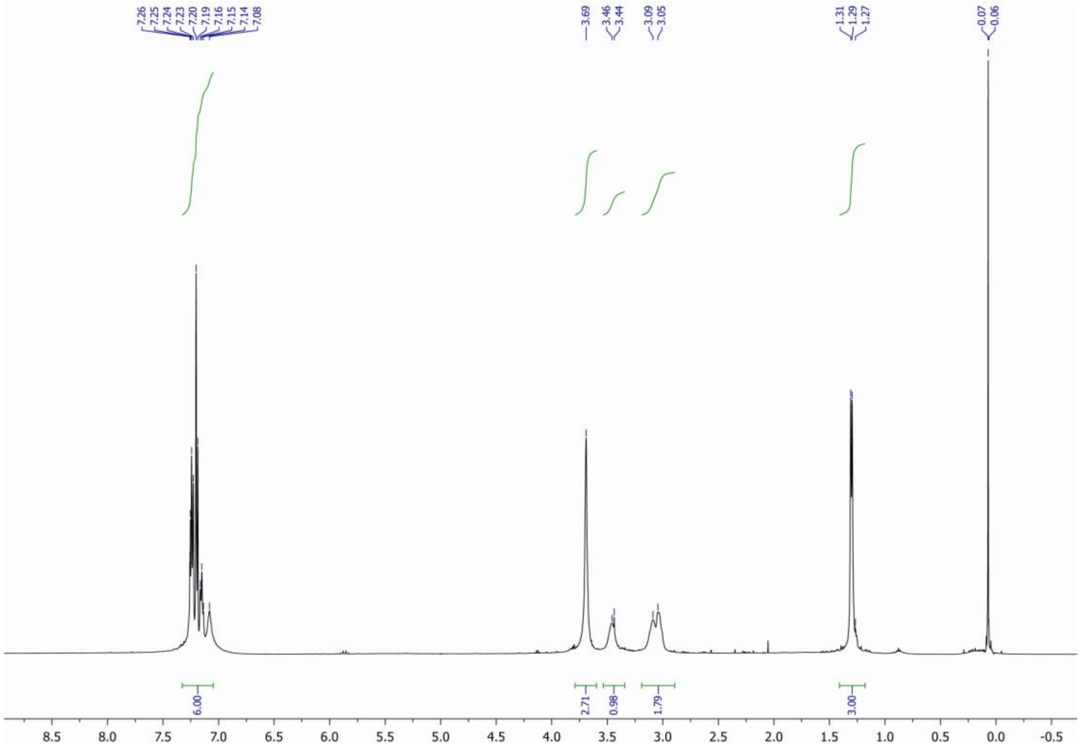

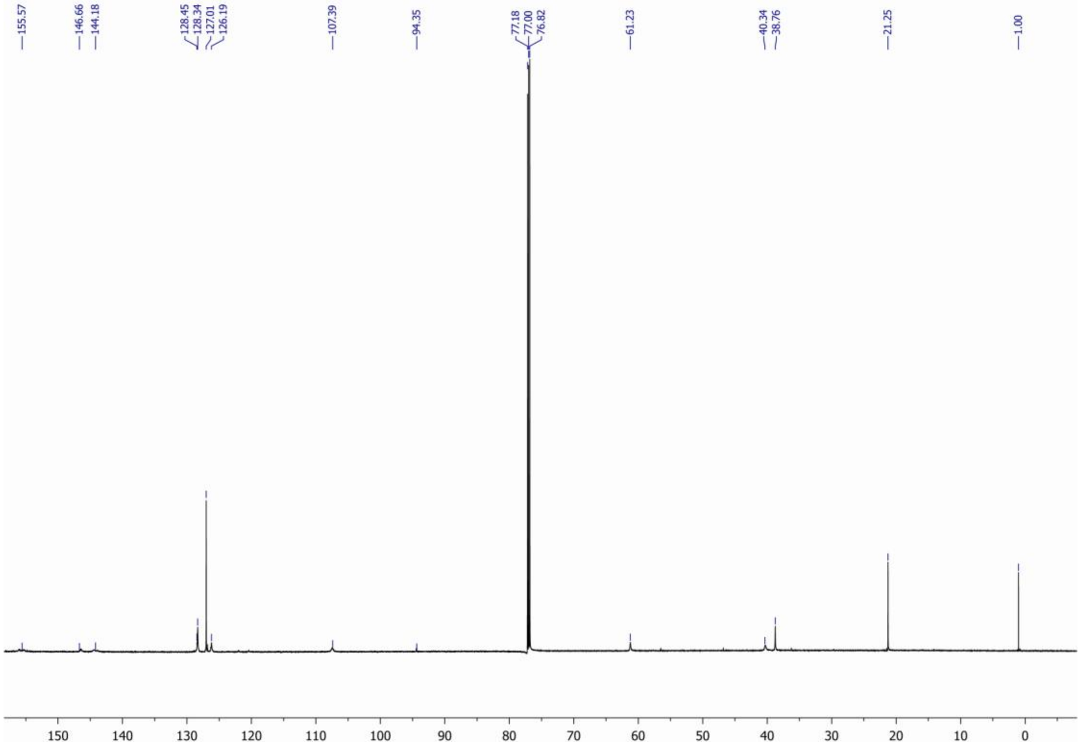

(*S*)-2-*sec*-Butyl-3-methoxy-6-(trifluoromethyl)pyridin-4-ol (**40**)

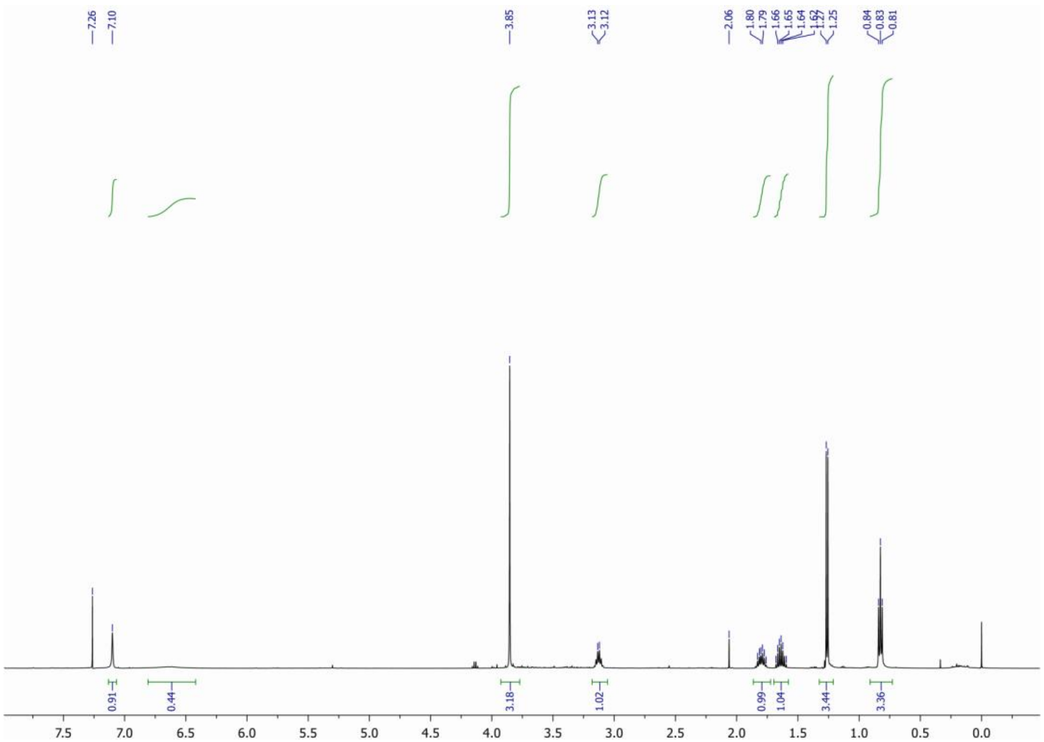

(*S*)-(3-Methoxy-5,5-dimethyl-4-oxohex-1-en-2-yl) 1-tritylpyrrolidine-2-carboxylate (**46**)

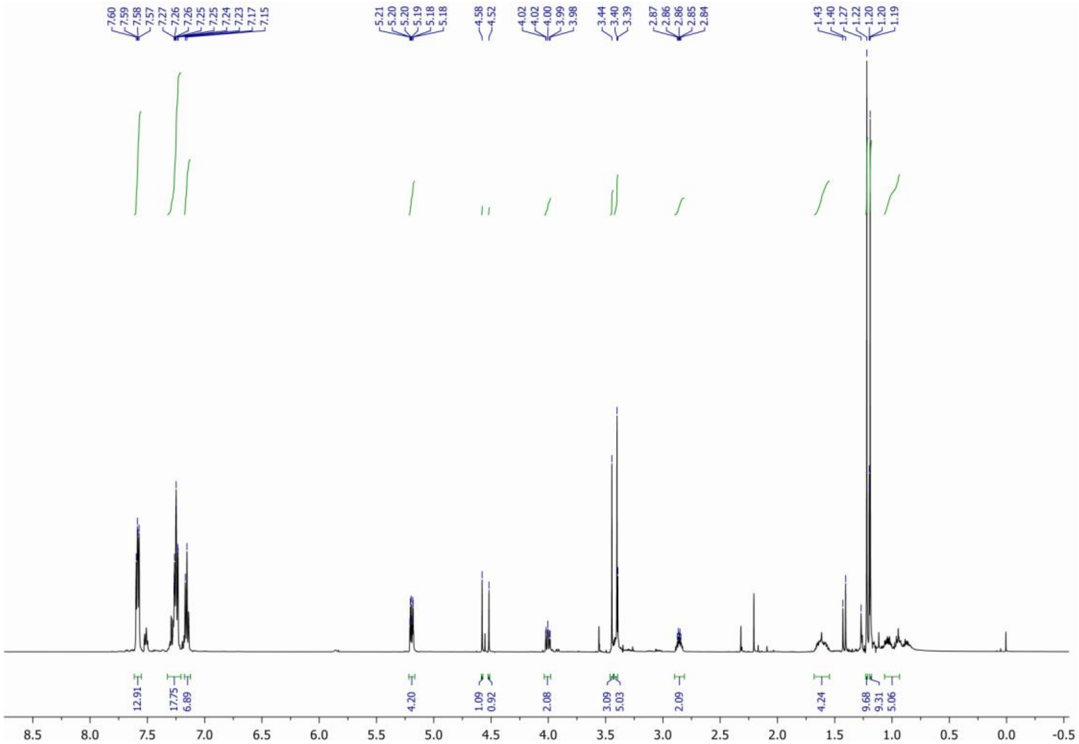

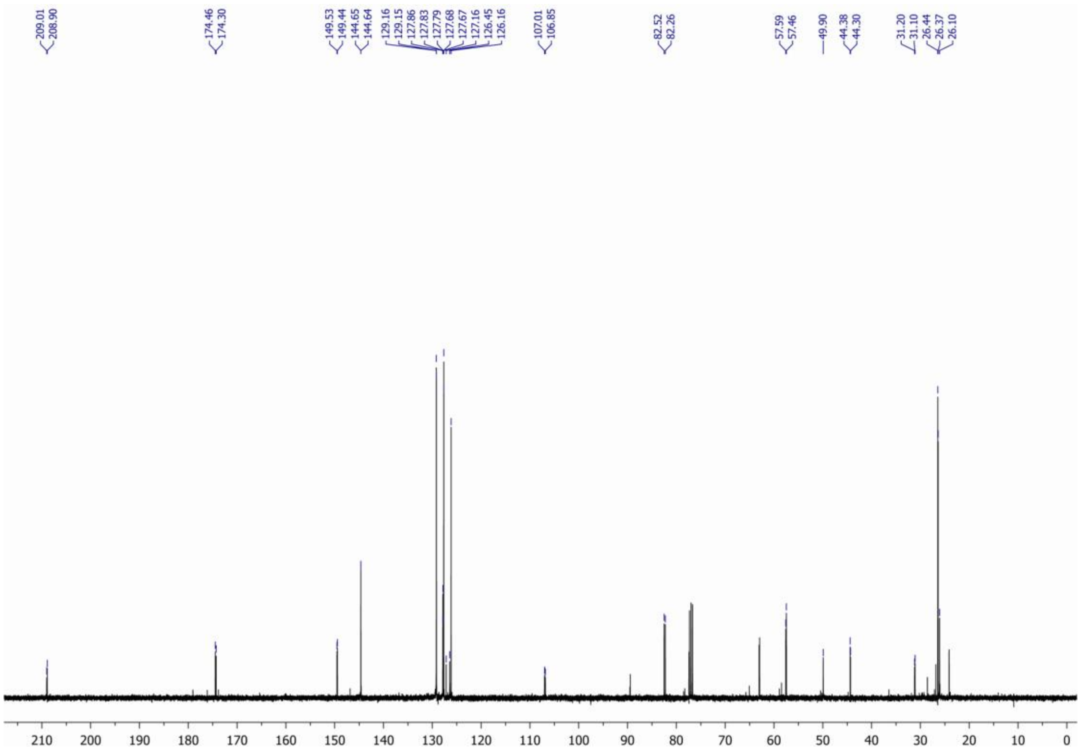

(*S*,*E*)-*N*-(3-Methoxy-5,5-dimethyl-4-oxohex-2-en-2-yl)-1-tritylpyrrolidine-2-carboxamide (**47**)

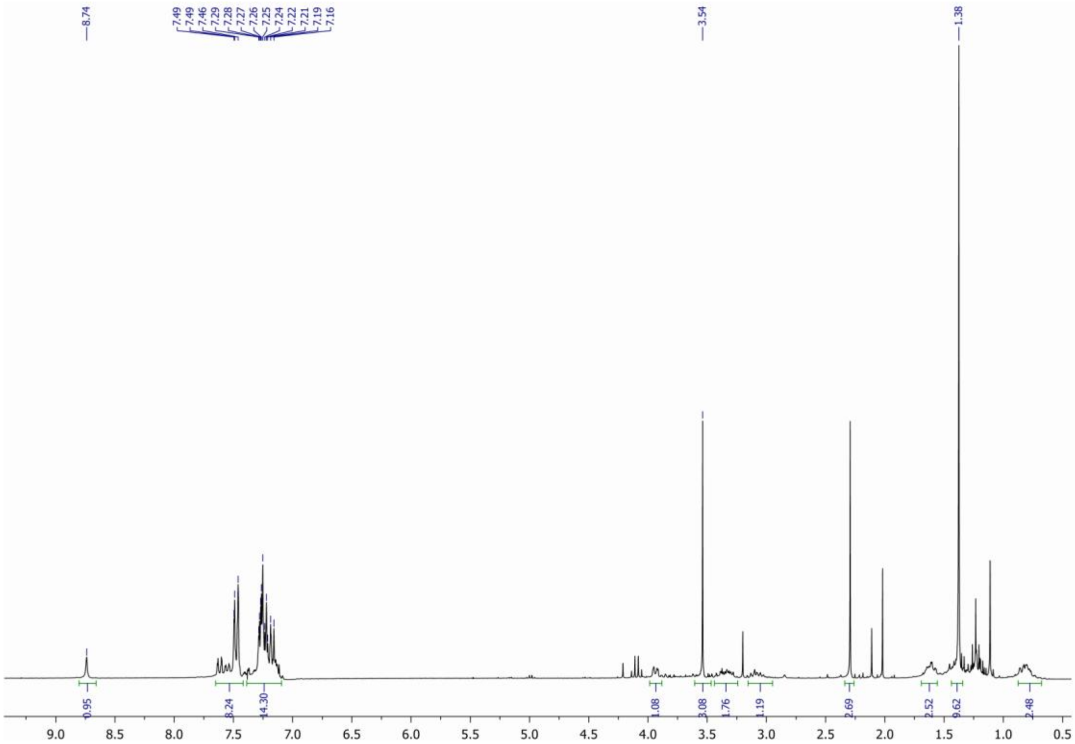

(*S*,*S*)-2-*sec*-Butyl-6-(trifluoromethyl)pyridin-4-yl 3,3,3-trifluoro-2-methoxy-2-phenylpropanoate (**51**)

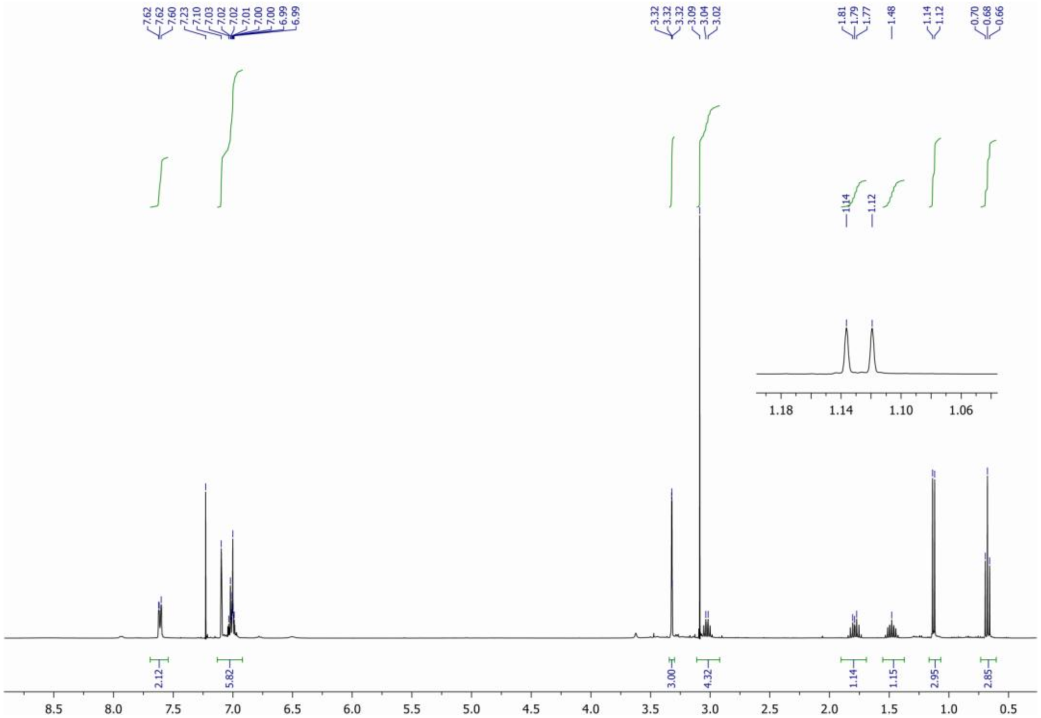

(*S*,*S*)-**51** and (*R*,*S*)-**51**

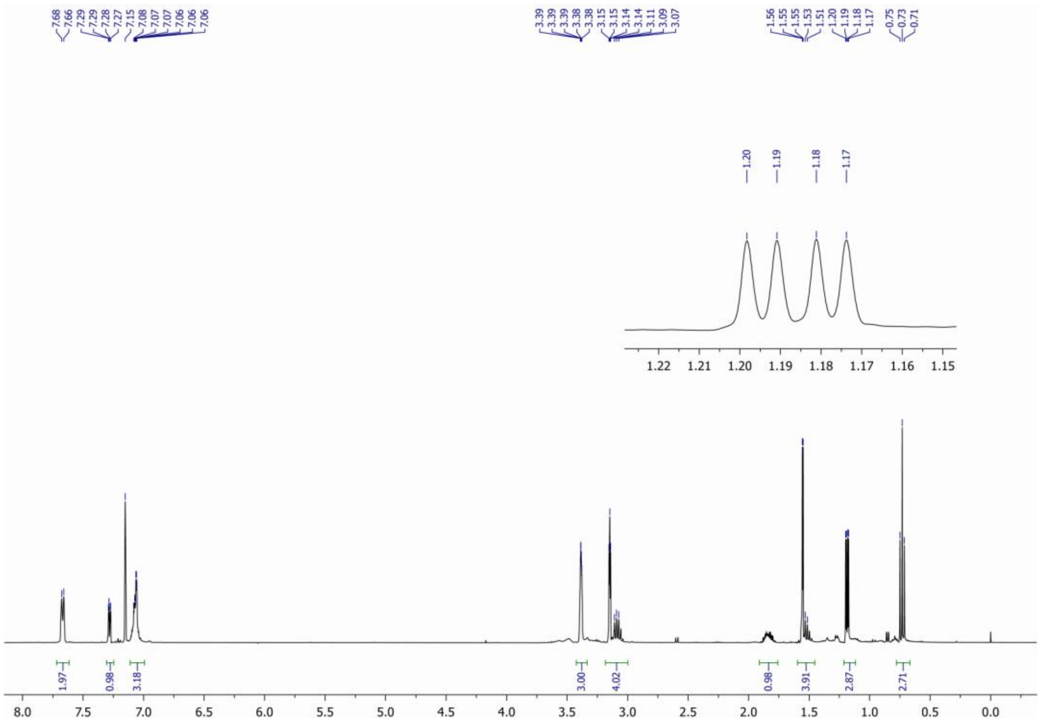

(*S*,*S*)-6-*sec*-Butyl-2-*tert*-butyl-3-methoxypyridin-4-yl 3,3,3-trifluoro-2-methoxy-2-phenylpropanoate (**50**)

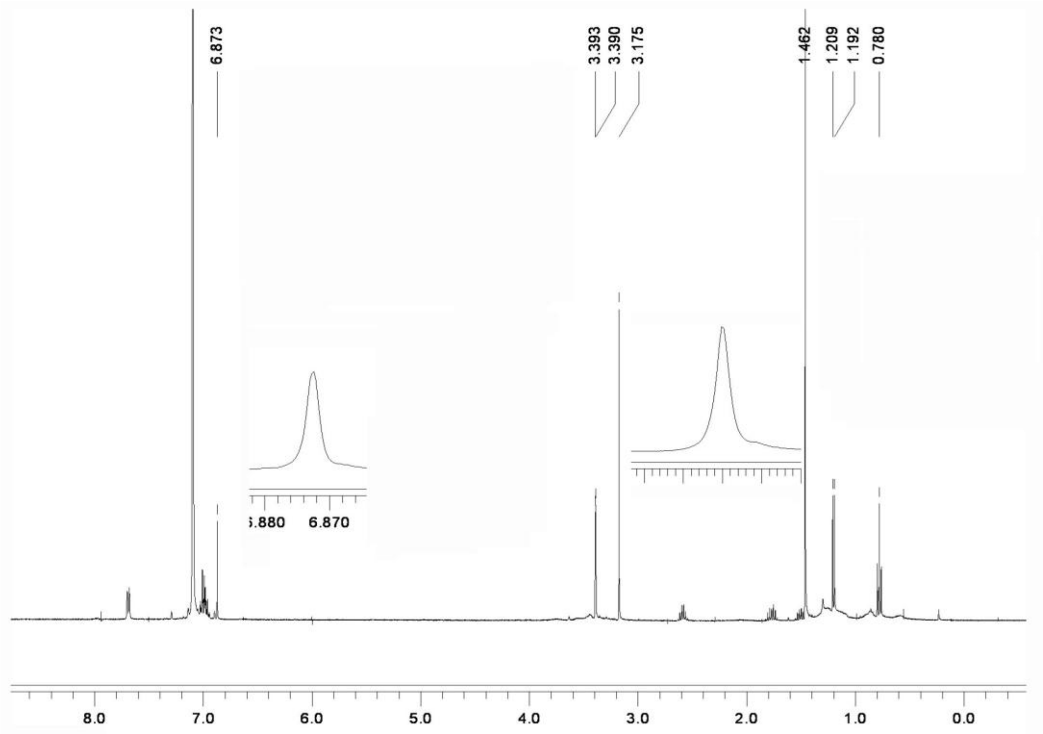

(*R*,*S*) and (*S*,*S*)-**50**

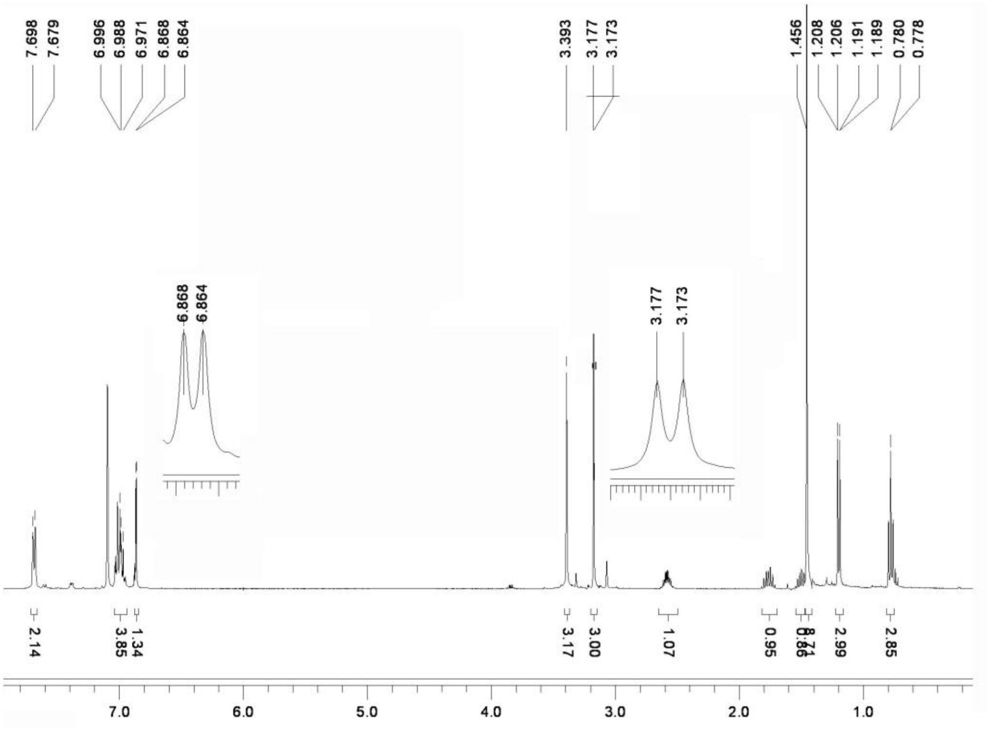

<u>(*S*,*S*)-2-tert-Butyl-6-[(*tert*-butyldimethylsiloxy)phenylmethyl]-3-methoxypyridin-4-yl 3,3,3-trifluoro-2-methoxy-2-phenylpropanoate (**49**)</u>

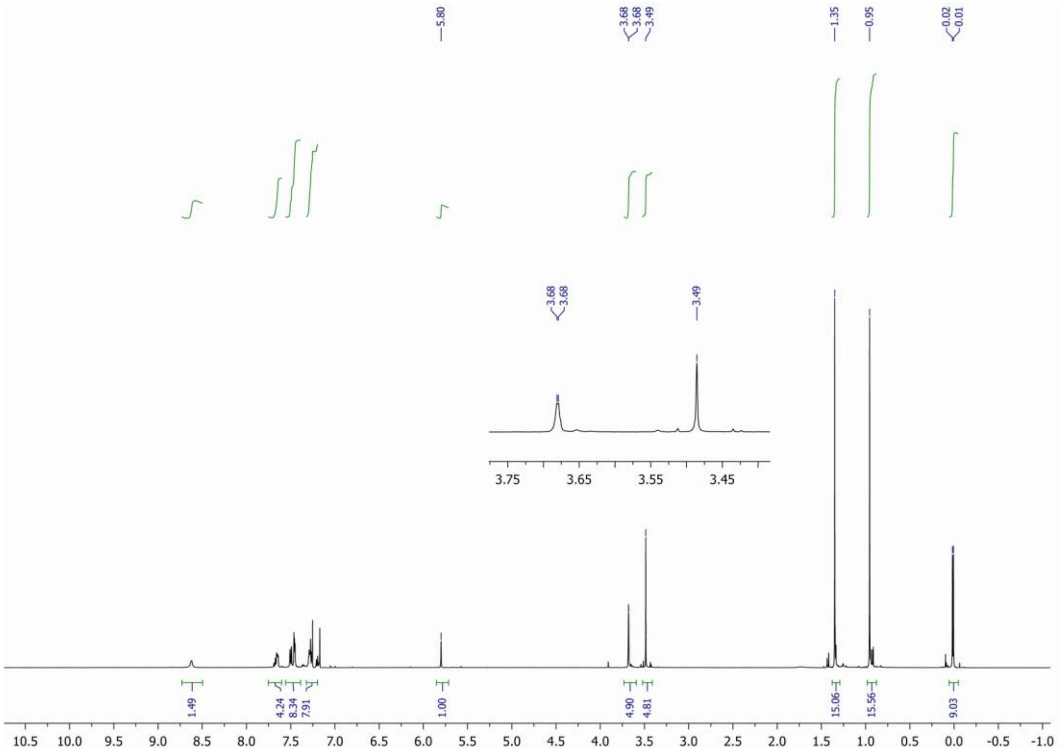

<u>(*R*,*S*)- and (*S*,*S*)-**49**</u>

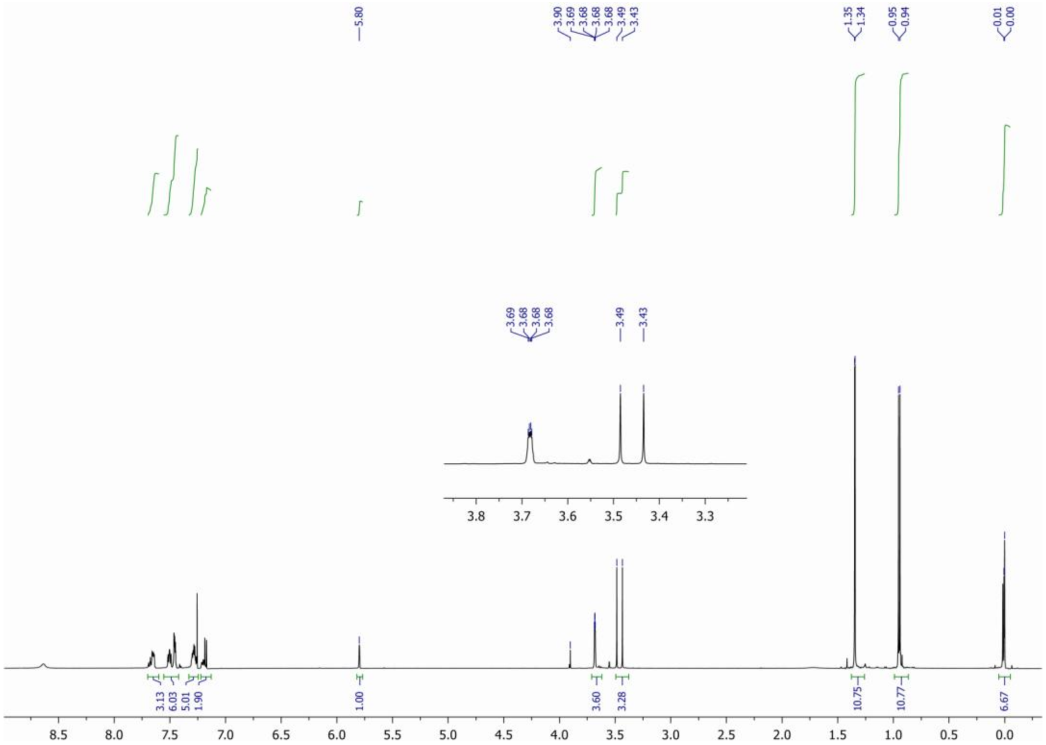

(*S*)-2-tert-Butyl-6-[(*tert*-butyldimethylsiloxy)phenylmethyl]-3,4-dimethoxypyridine (**53**)

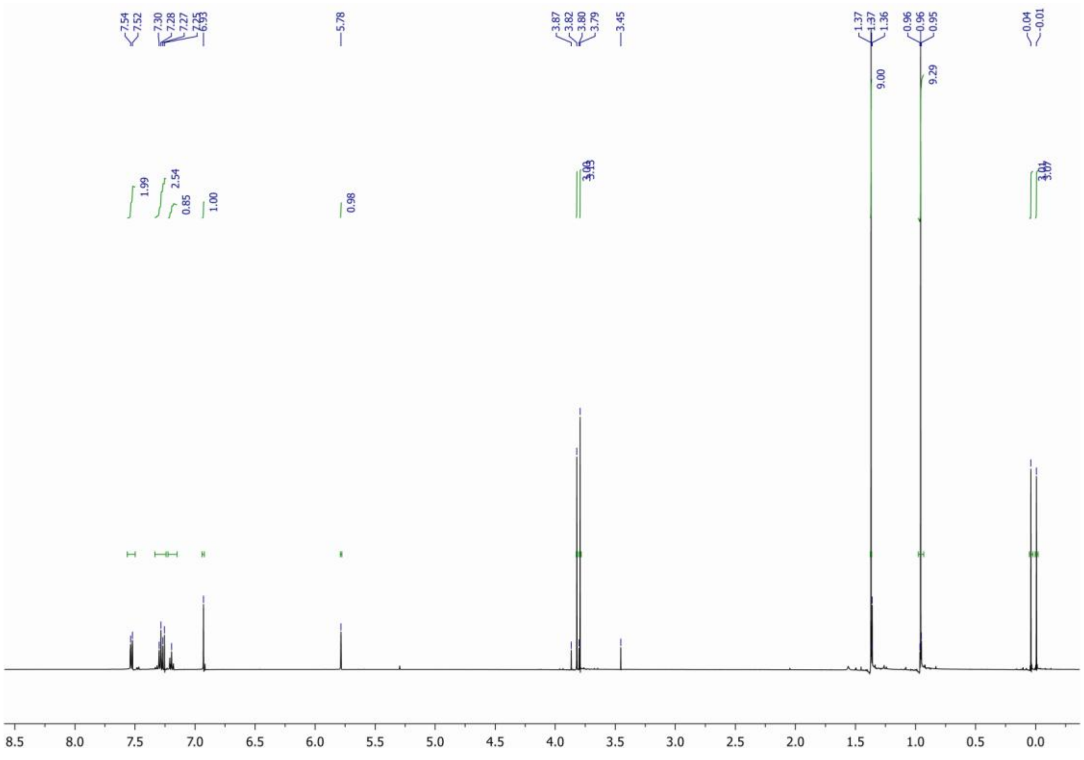

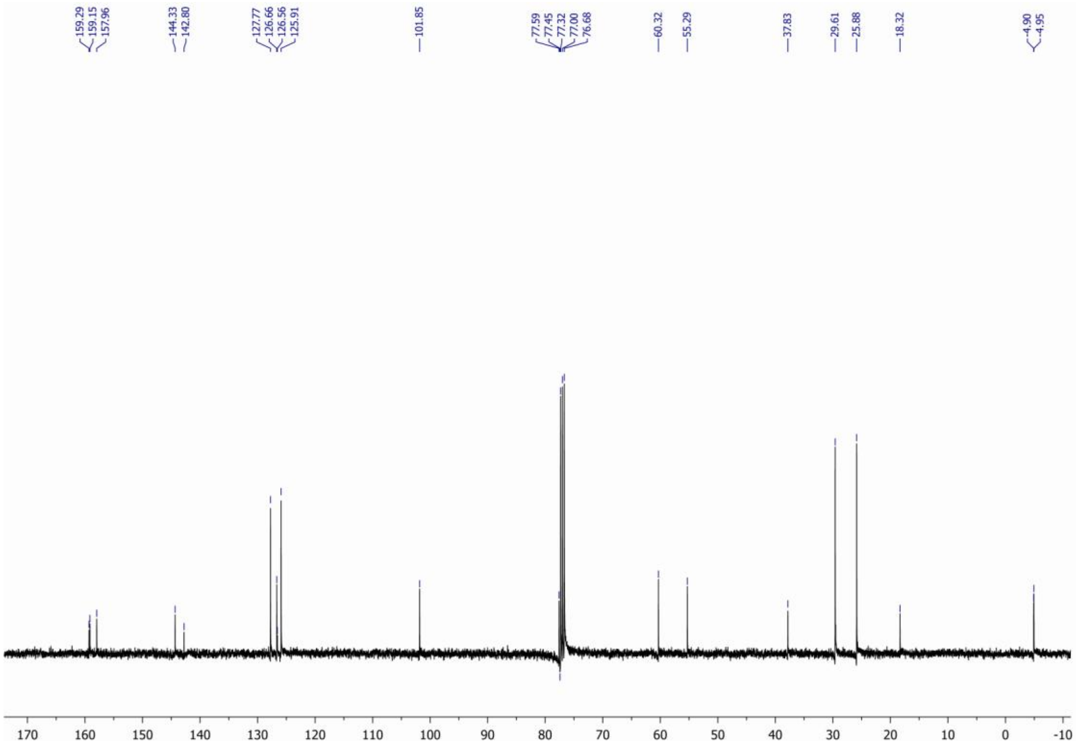

(*S*)-(6-*tert*-Butyl-4,5-dimethoxypyridin-2-yl)phenylmethanol (**54**)

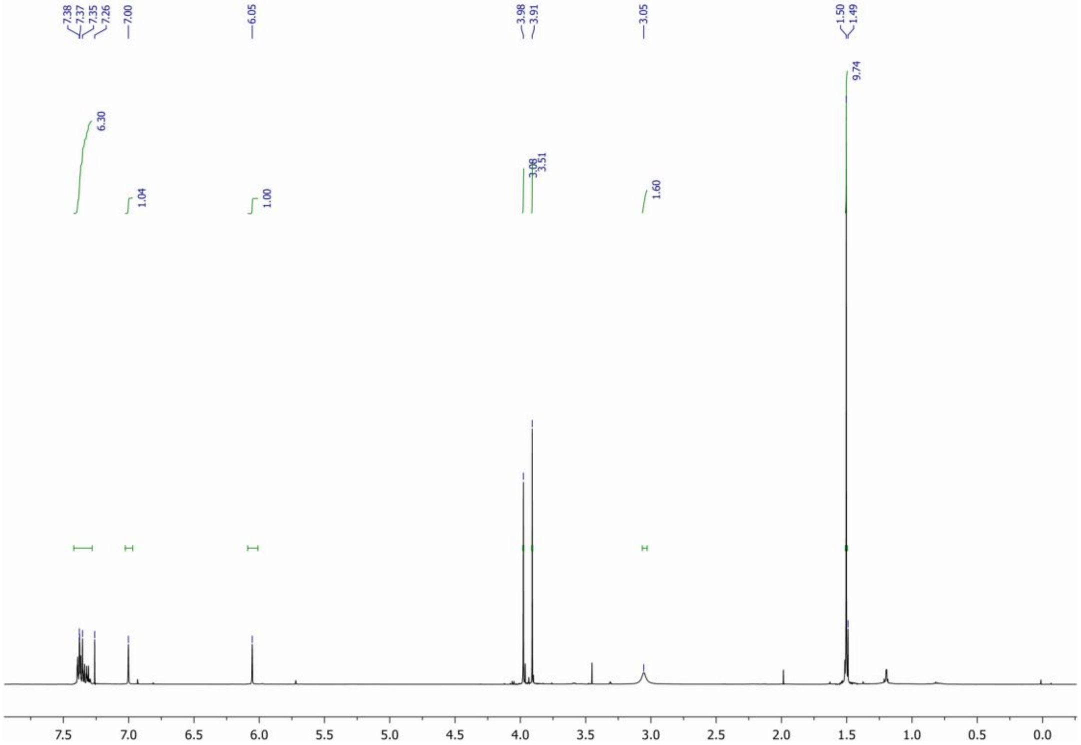

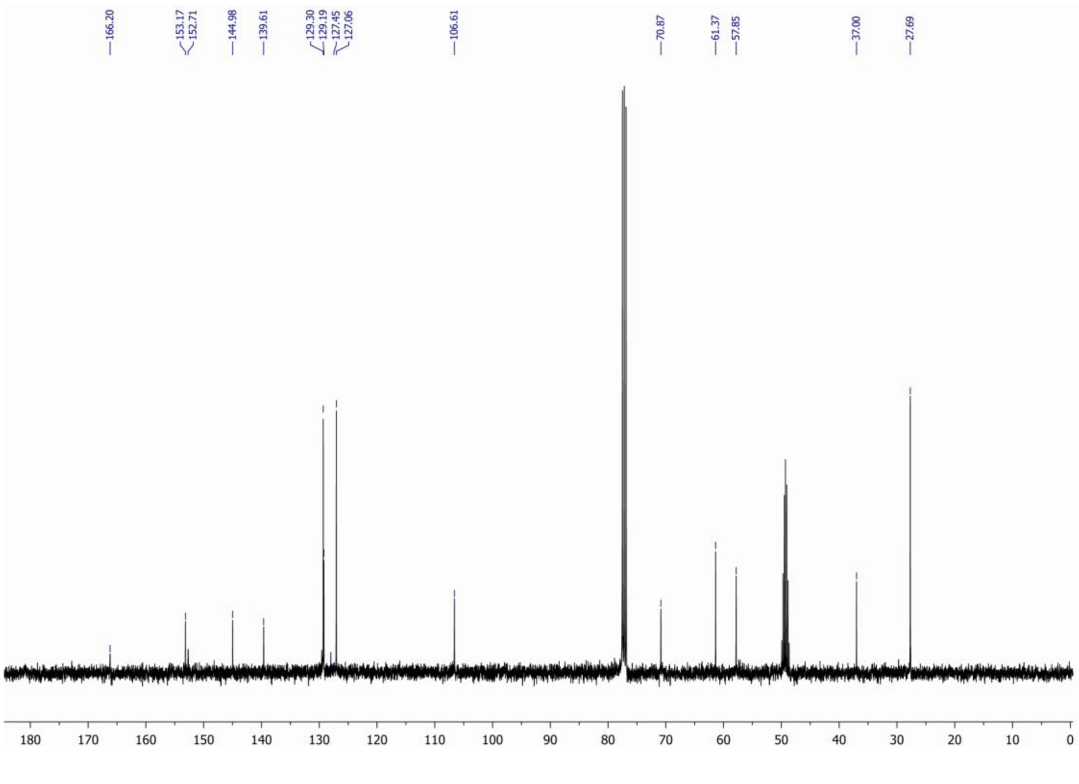

(*R*)-6-[(*tert*-Butyldimethylsiloxy)phenylmethyl]-3,4-dimethoxy-2-phenylpyridine (**55**)

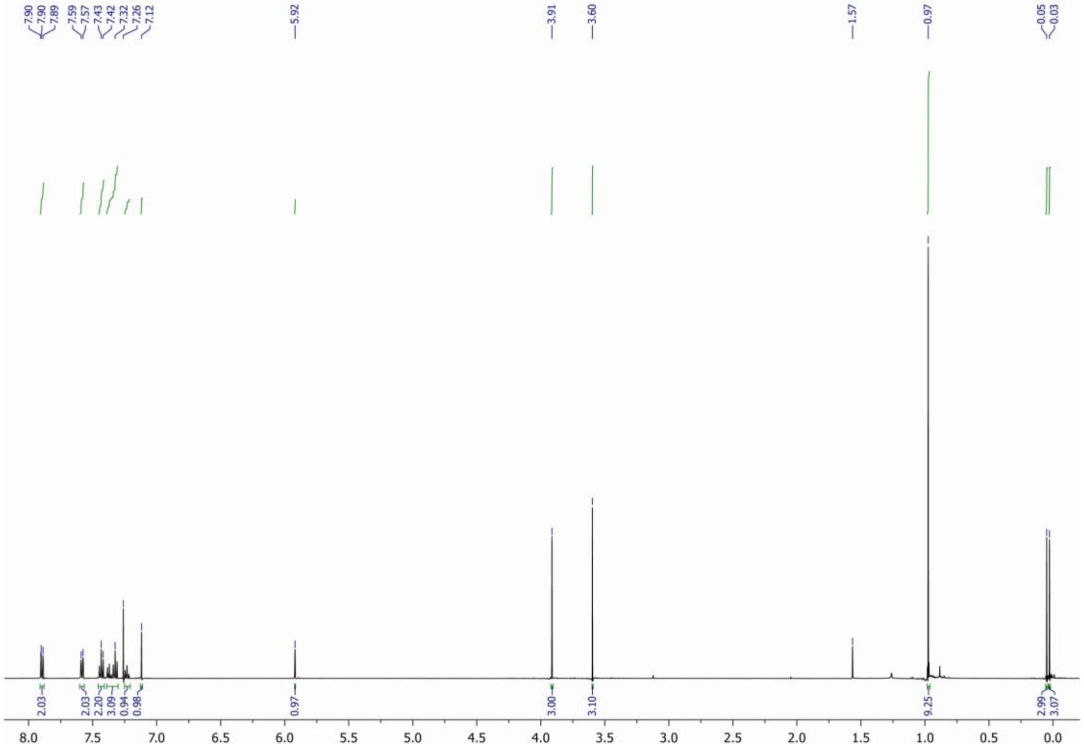

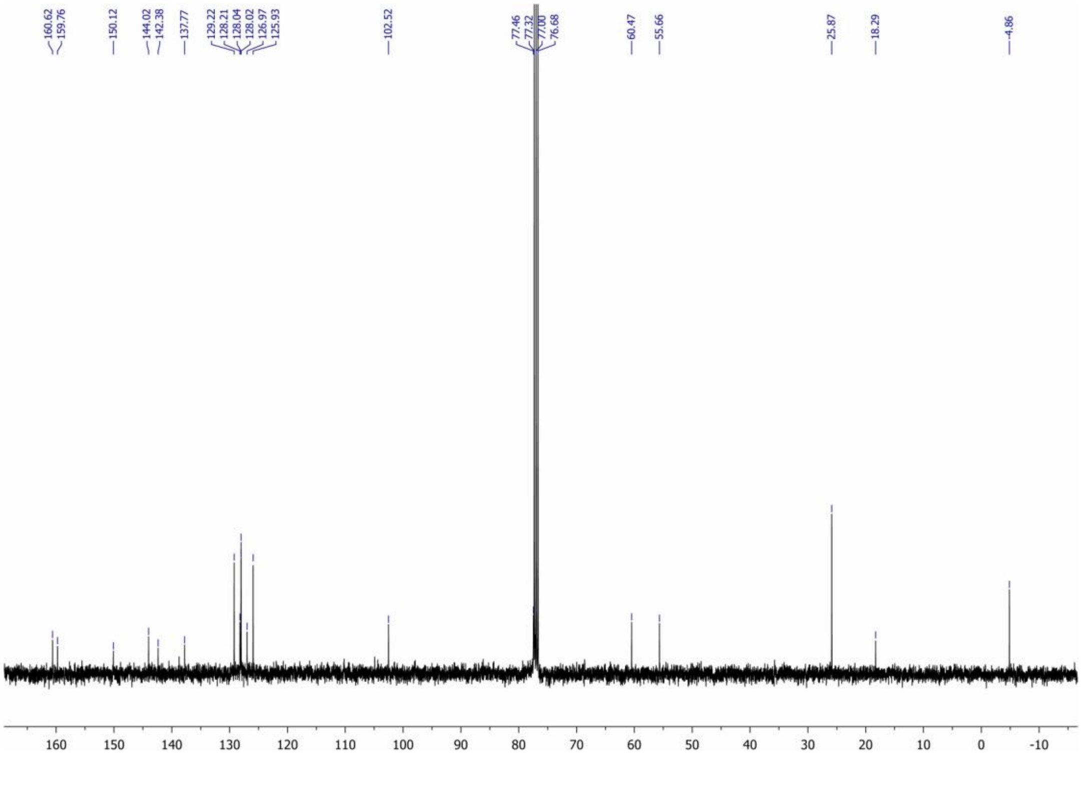

(*S*)-2-(*tert*-Butyldimethylsiloxy)-*N*-(1-*tert*-butyl-2-methoxy-3-oxo-but-1-enyl)-propionamide (**61**)

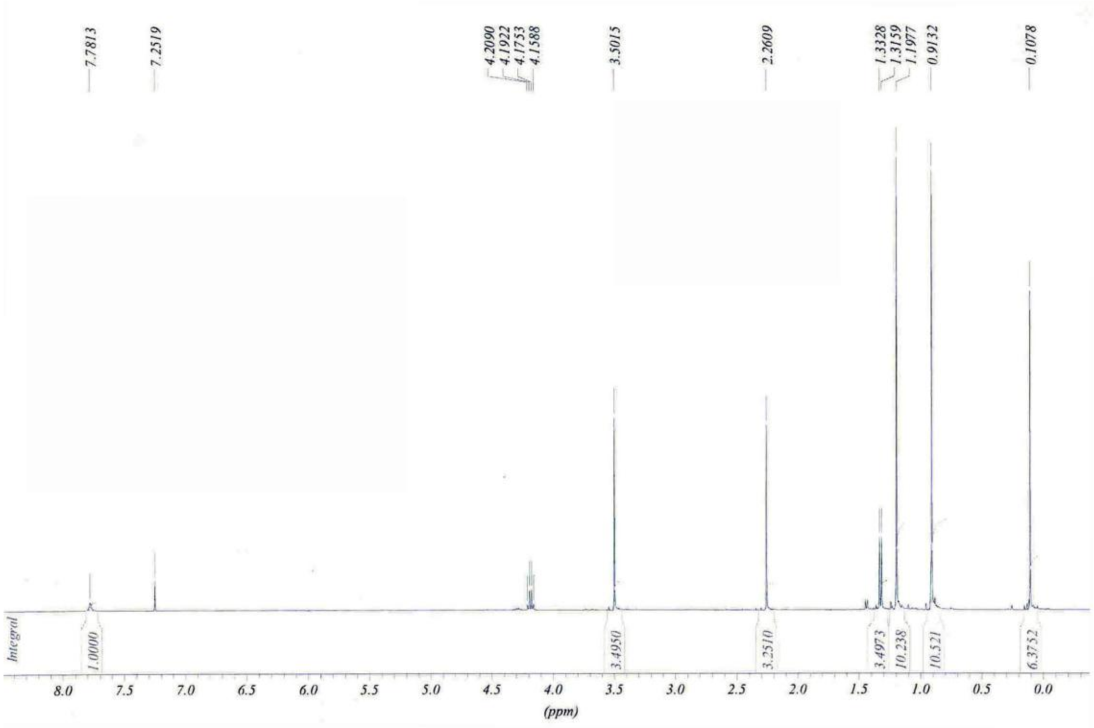

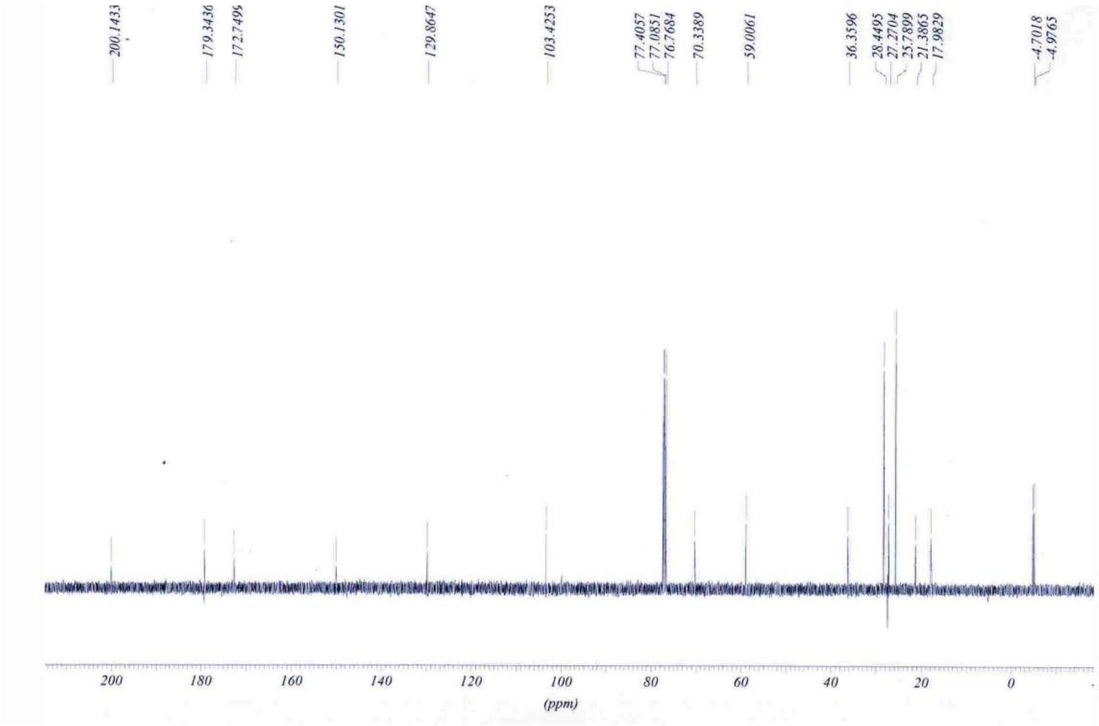

(*S*)-2-(*tert*-Butyldimethylsiloxy)-*N*-(2-methoxy-3-oxo-1-phenyl-but-1-enyl)-propionamide (**62**)

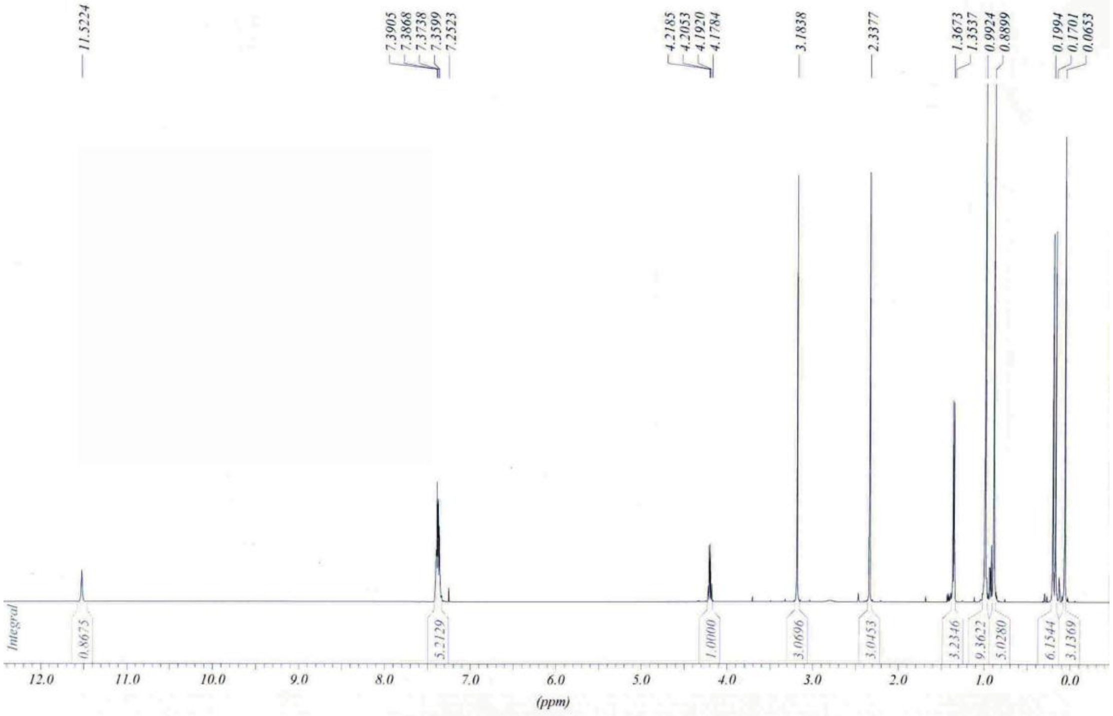

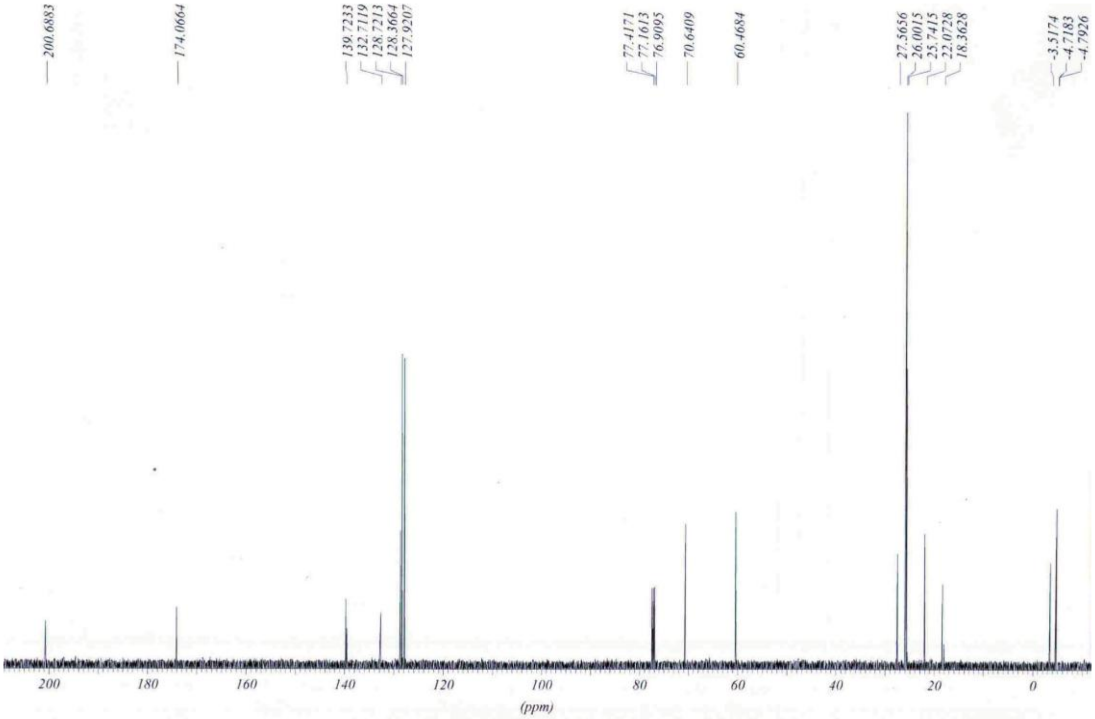

(*S*)-N-{1-[1-(*tert*-Butyldimethylsiloxy)-ethyl]-2-methoxy-3-oxo-but-1-enyl}c-benzamide (**64**)

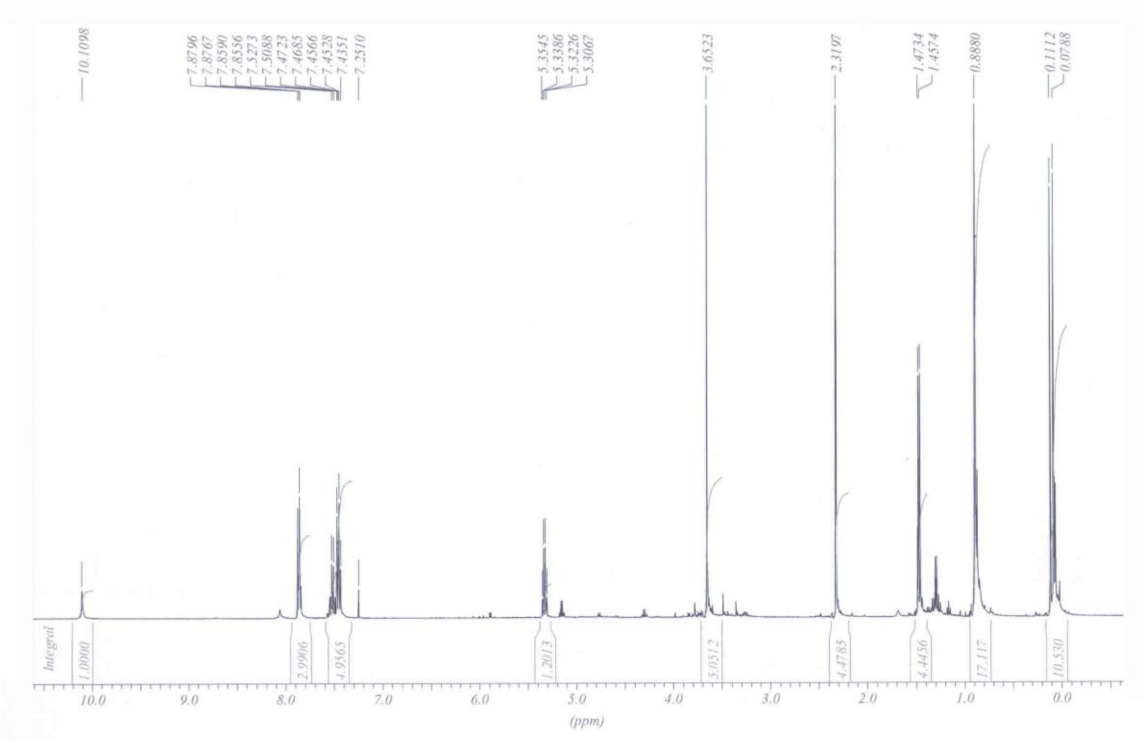

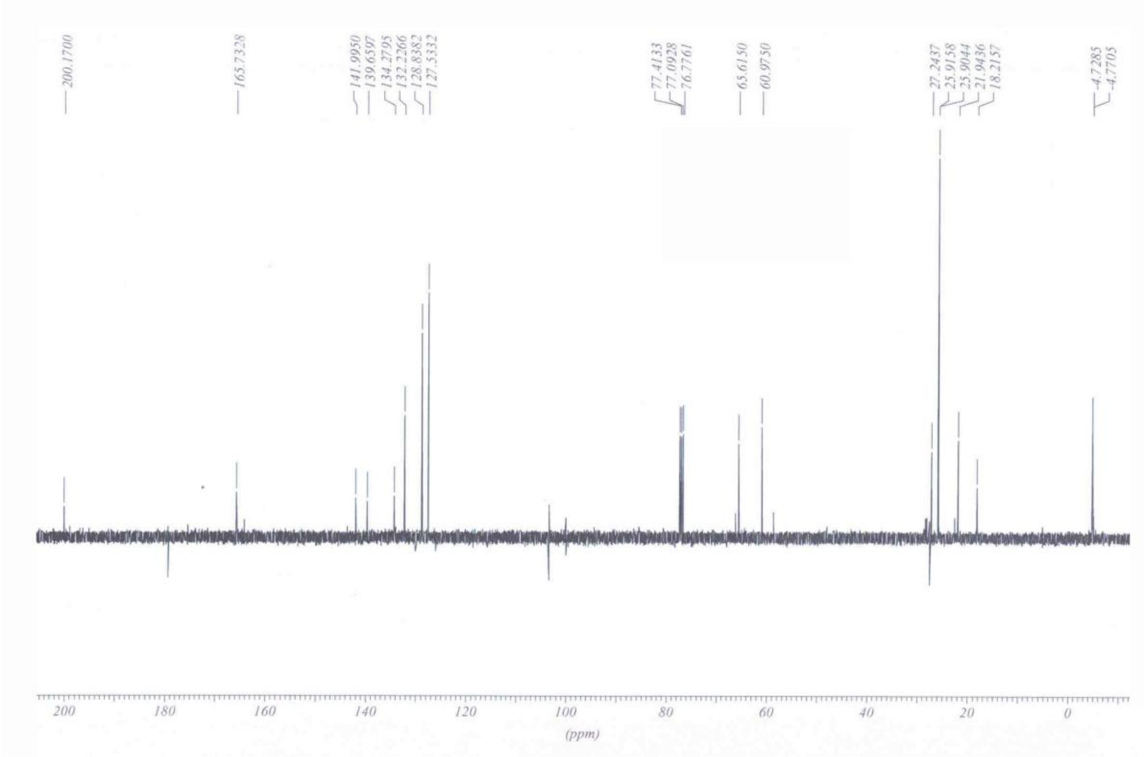

(*S, E*)-Thiophene-2-carboxylic acid {1-[1-(*tert*-butyldimethylsiloxy)-ethyl]-2-methoxy-3-oxo-but-1-enyl}-amide (**65**)

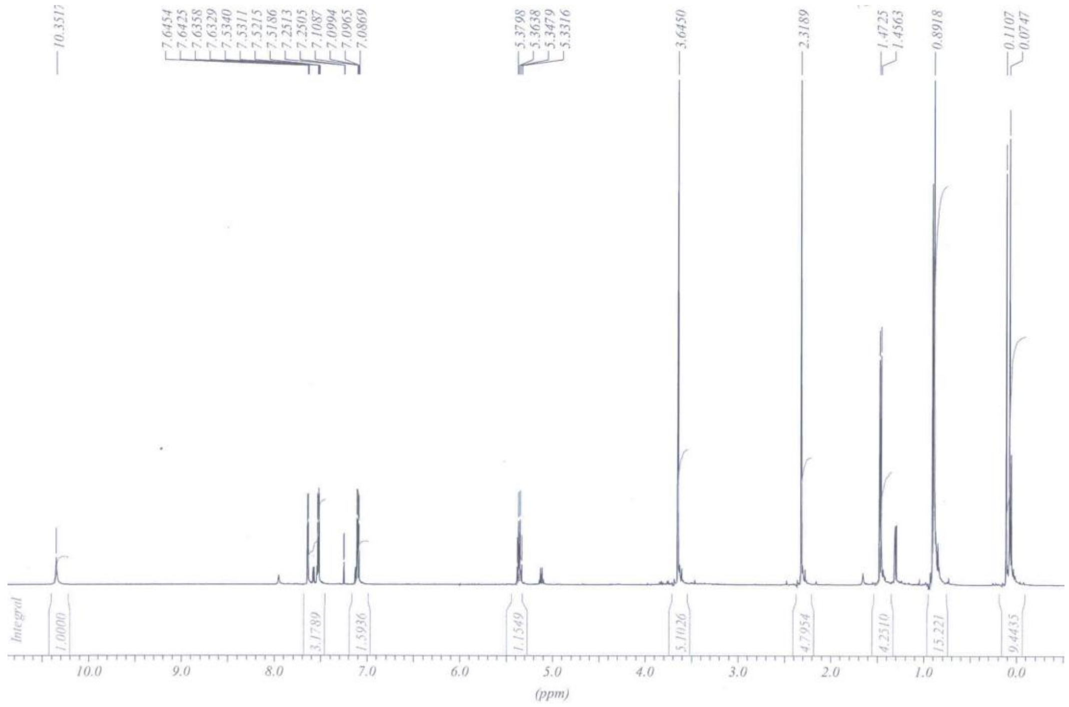

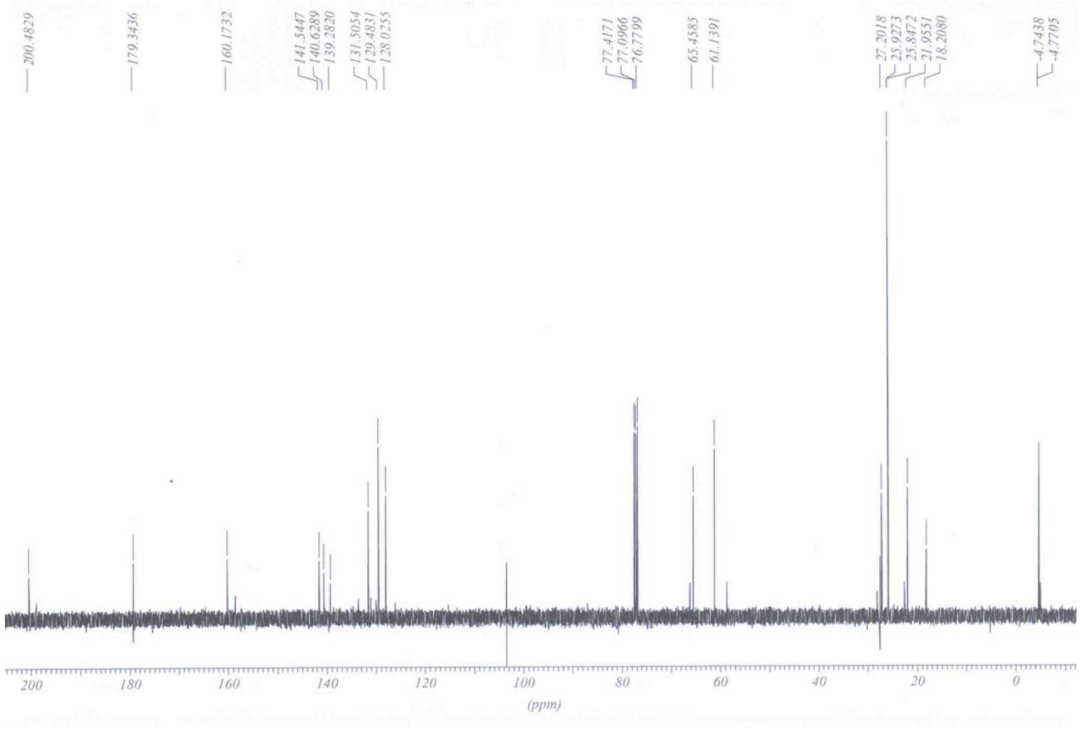

(*S*)-Pyridine-2-carboxylic acid {1-[1-(*tert*-butyldimethylsiloxy)-ethyl]-2-methoxy-3 oxobut-1-enyl}amide (**66**)

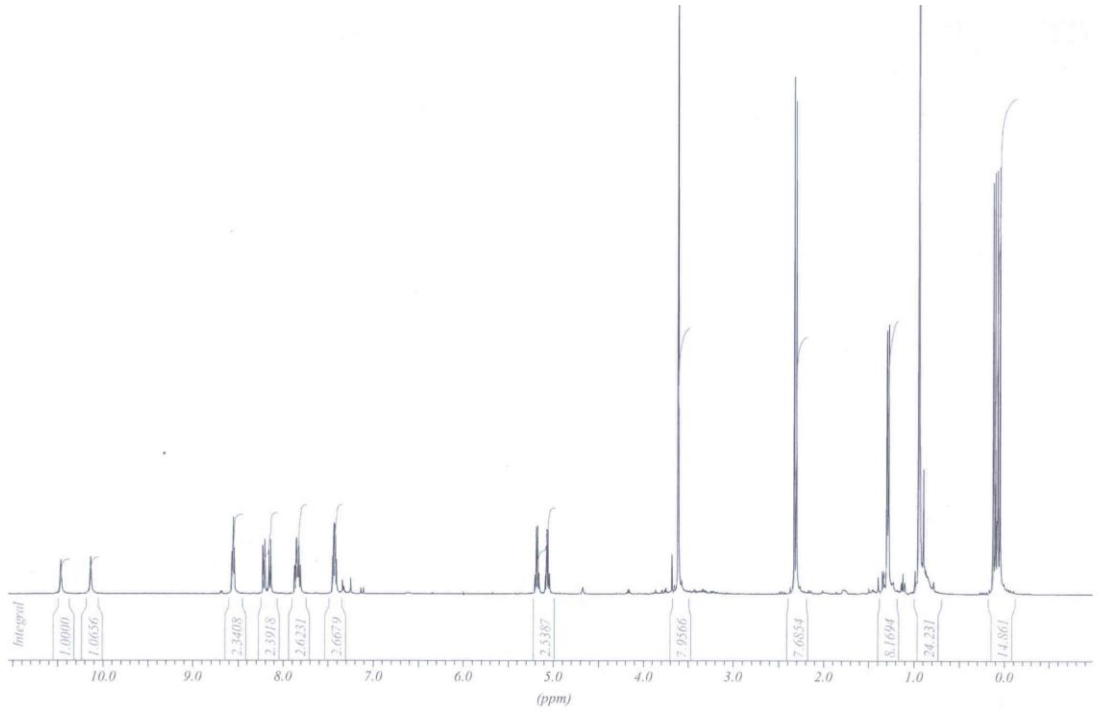

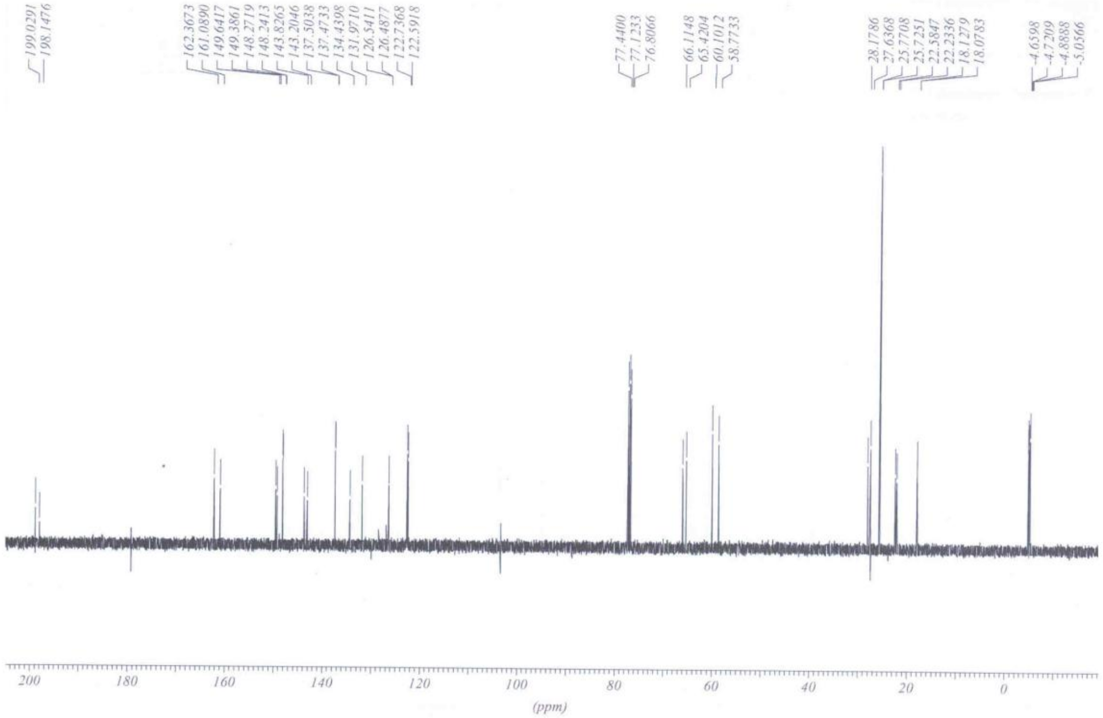

(*S*,*S*)-2-(*tert*-Butyldimethylsiloxy)-*N*-{1-[1-(*tert*-butyldimethylsiloxy)-ethyl]-2-methoxy-3-oxo-but-1-enyl}-propionamide (**67**)

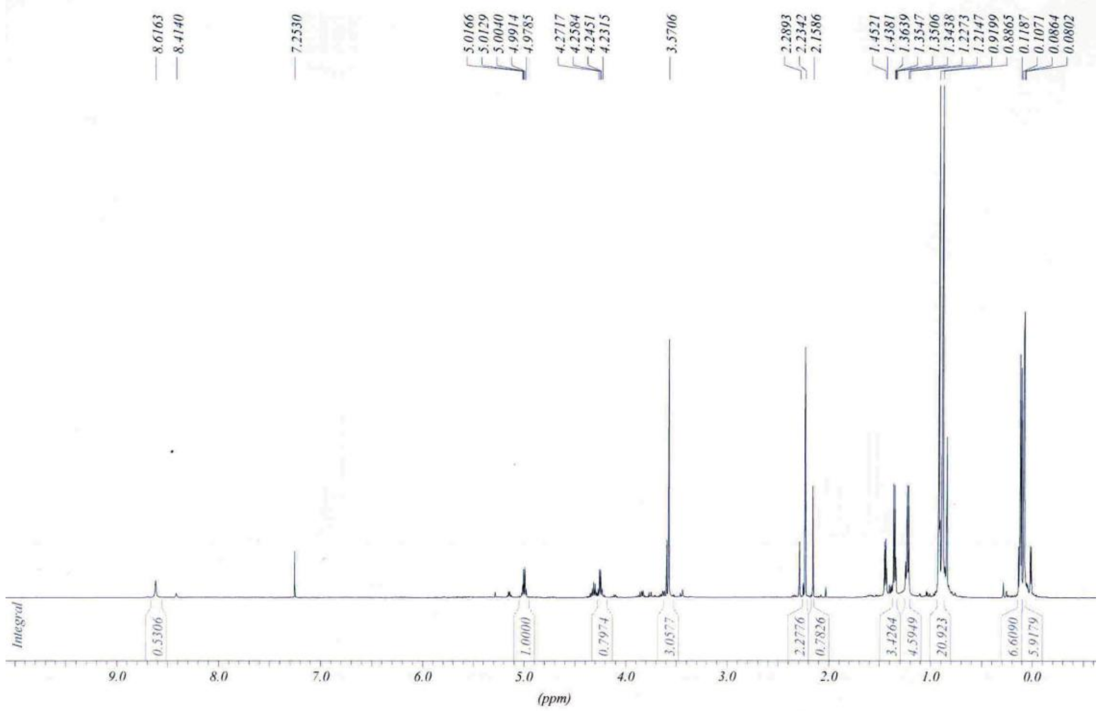

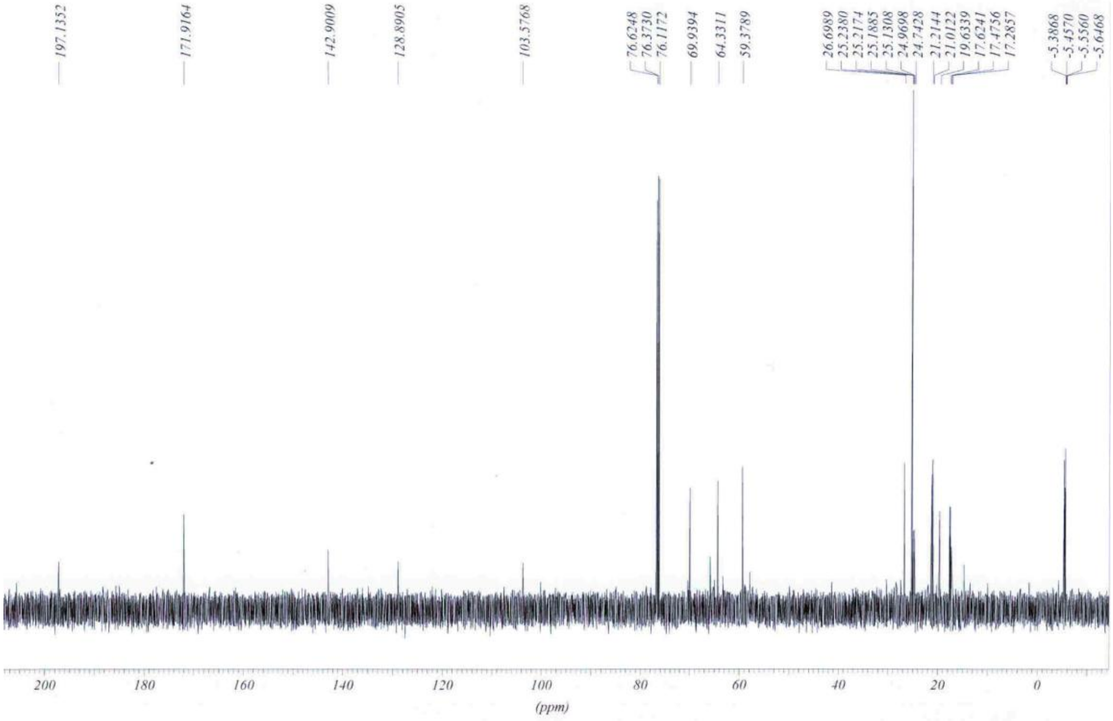

(*S*)-2-*tert*-Butyl-6-[1-(*tert*-butyldimethylsiloxy)-ethyl]-3-methoxy-pyridin-4-yl nonaflate (**68**)

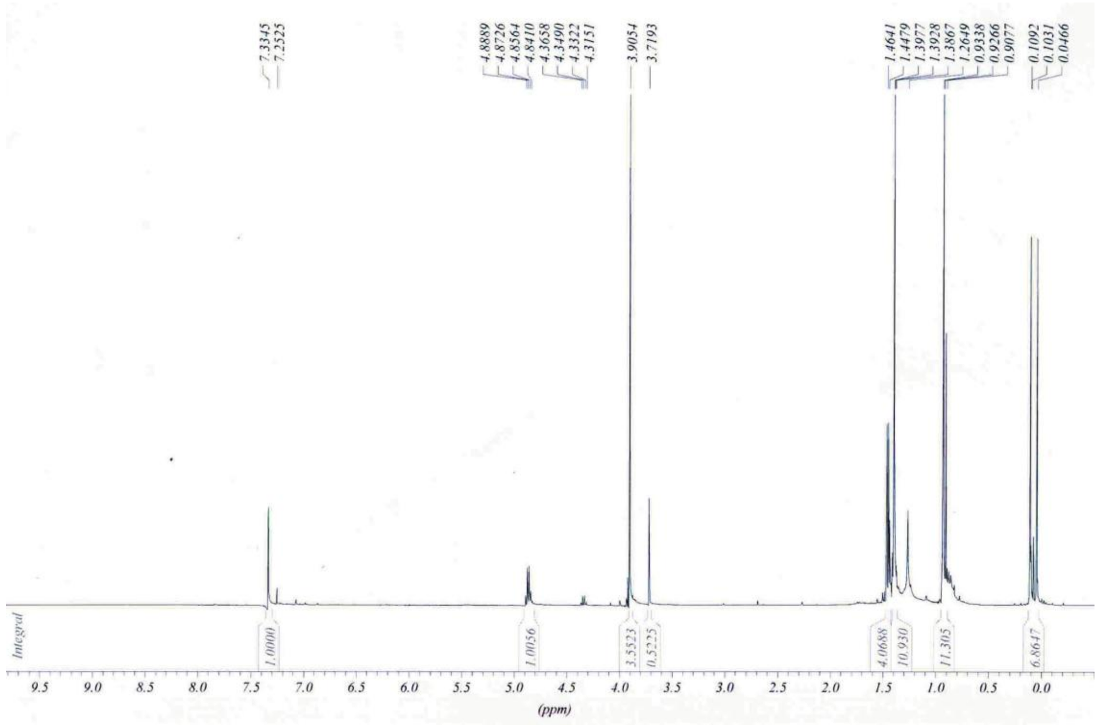

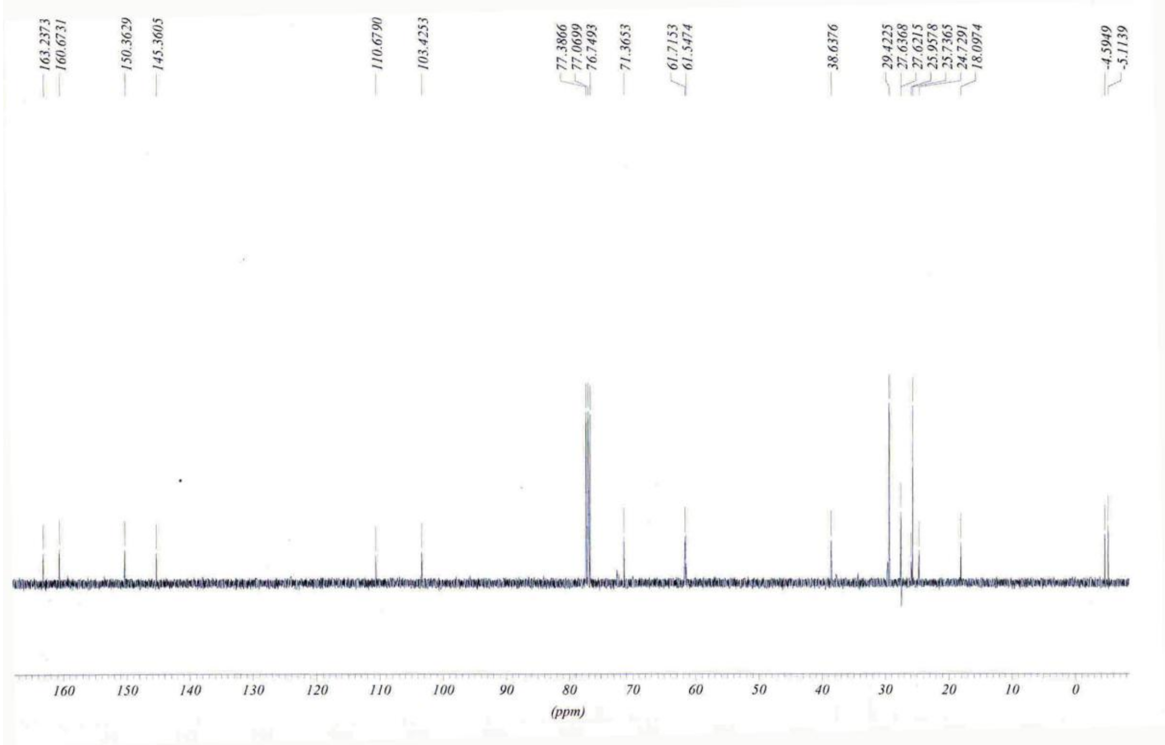

(*S*)-6-[1-(*tert*-Butyldimethylsiloxy)-ethyl]-3-methoxy-2-phenyl-pyridin-4-yl nonaflate (**69**)

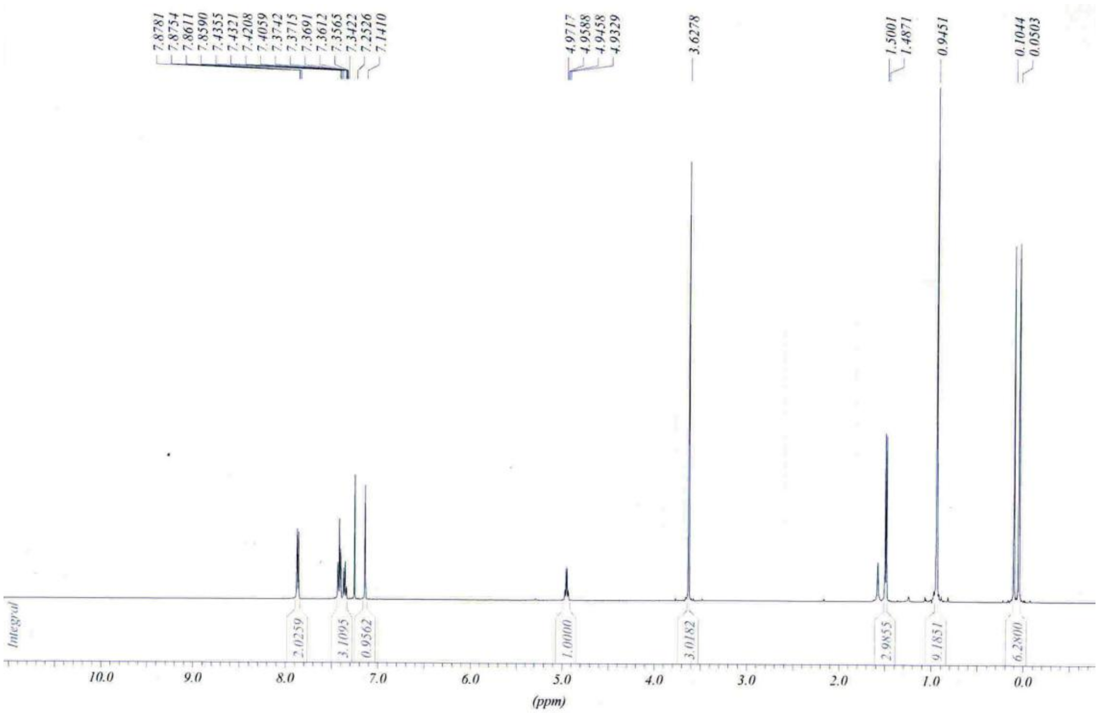

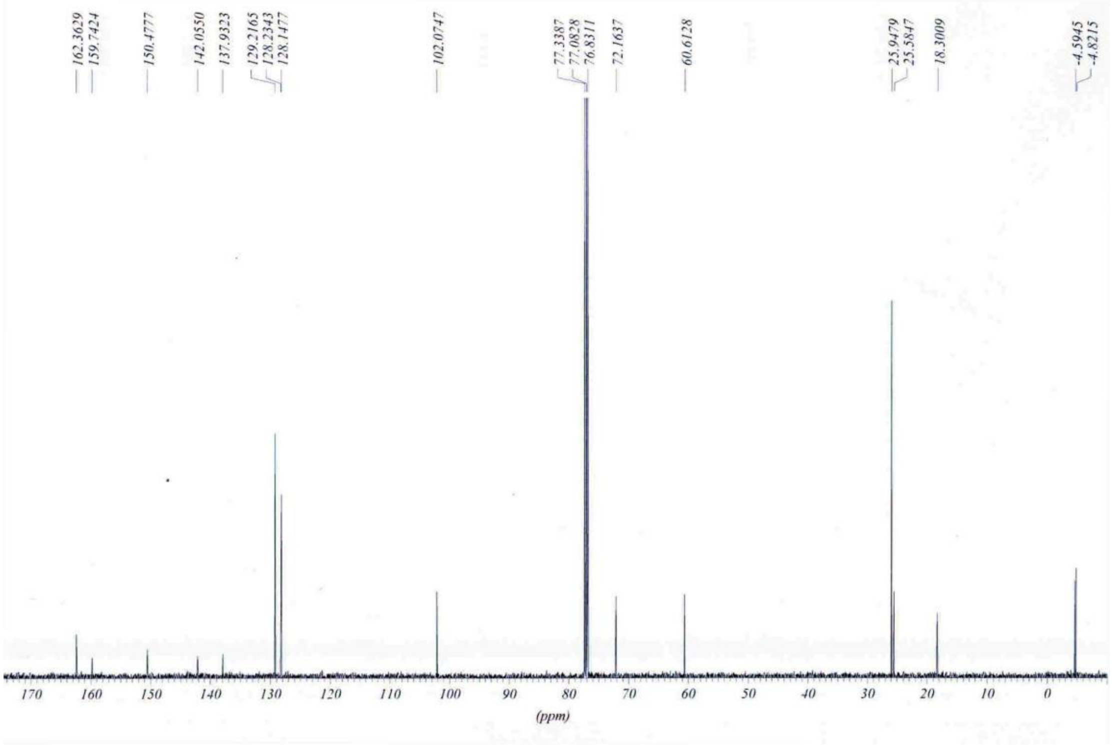

(*S*)-2-[1-(*tert*-Butyldimethylsiloxy)-ethyl]-3-methoxy-6-phenyl-pyridin-4-yl nonaflate (**70**)

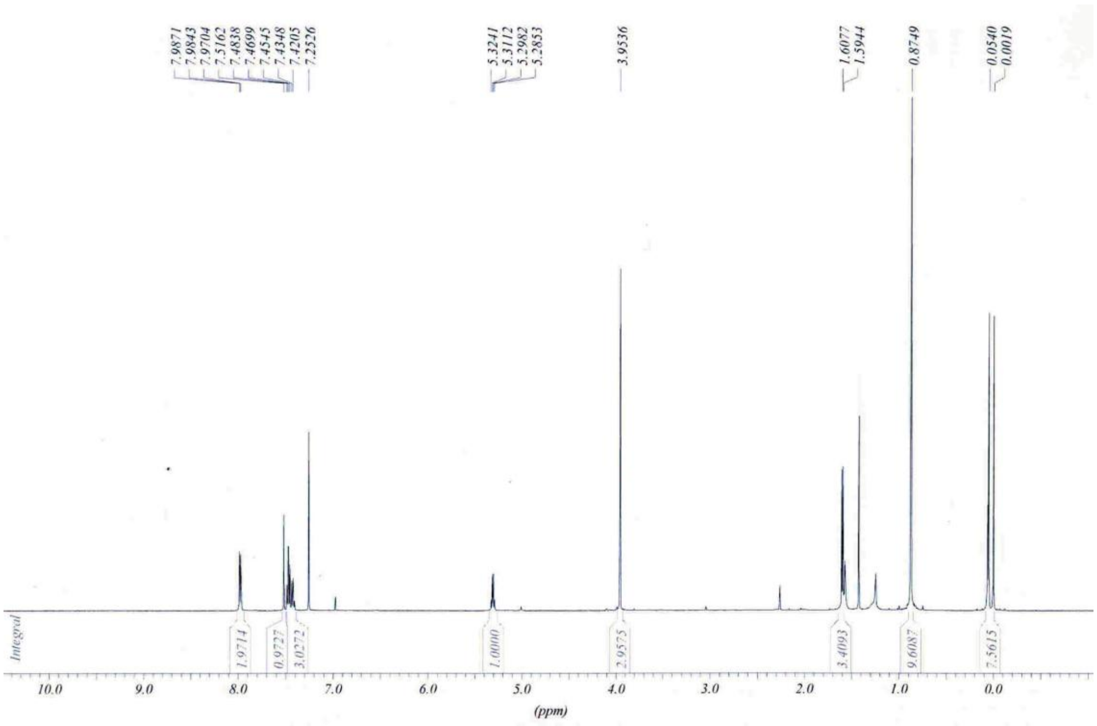

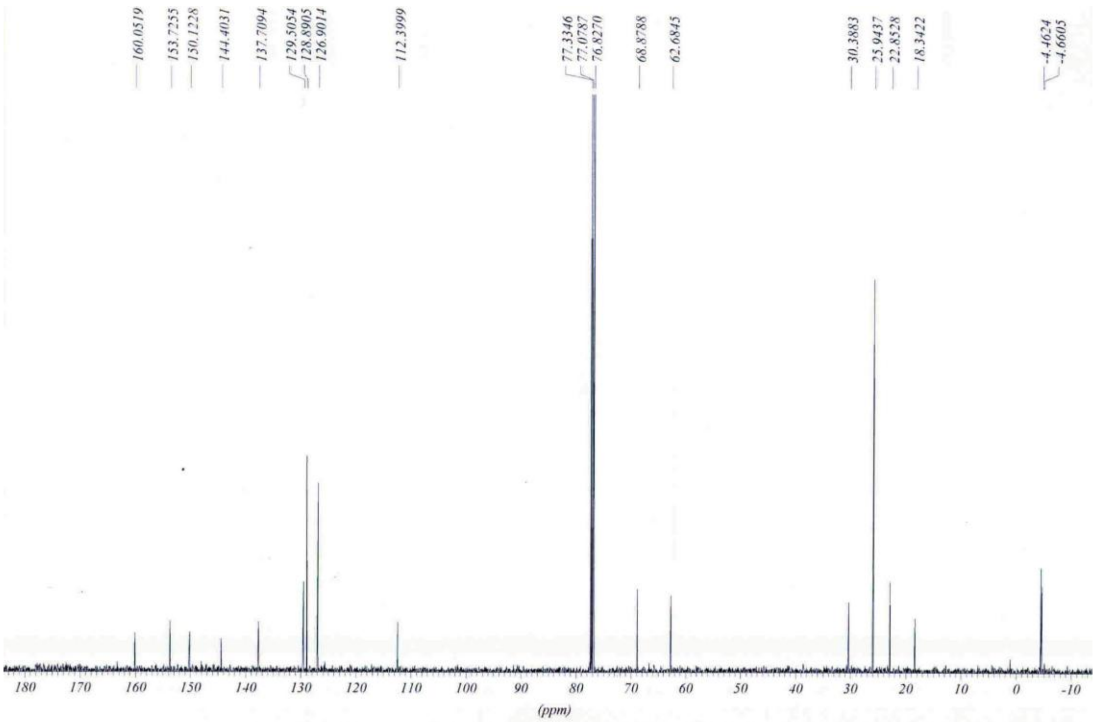

(*S*)-2-[1-(*tert*-Butyldimethylsiloxy)-ethyl]-3-methoxy-6-thiophen-2-yl-pyridin-4-yl nonaflate (**71**)

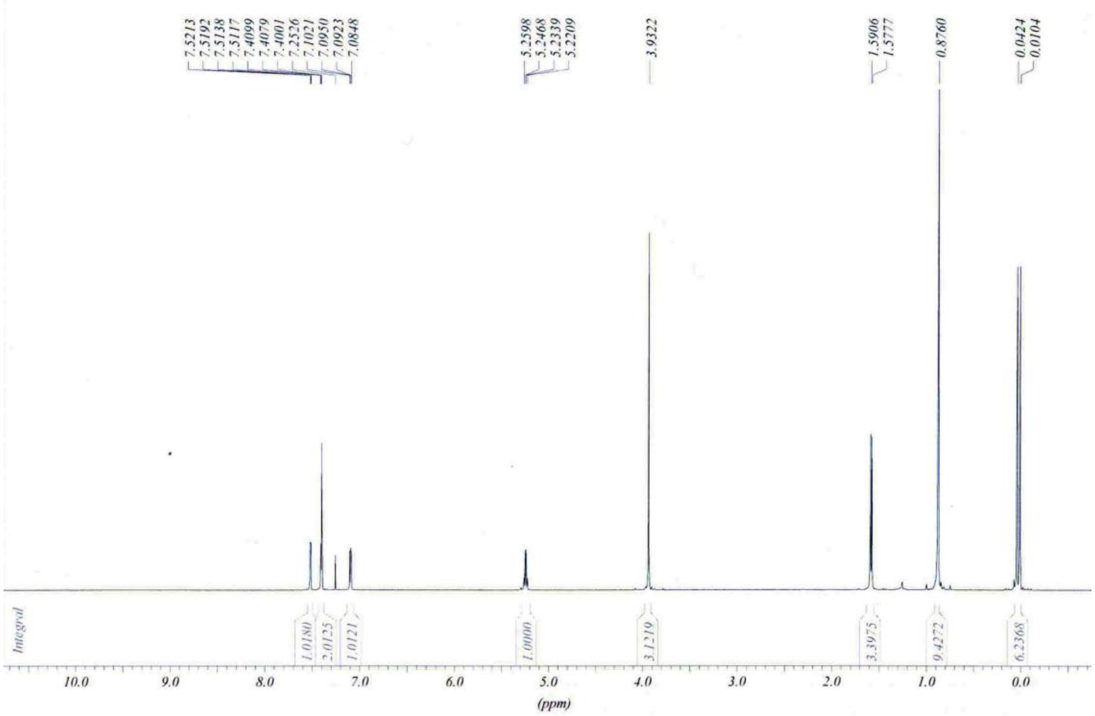

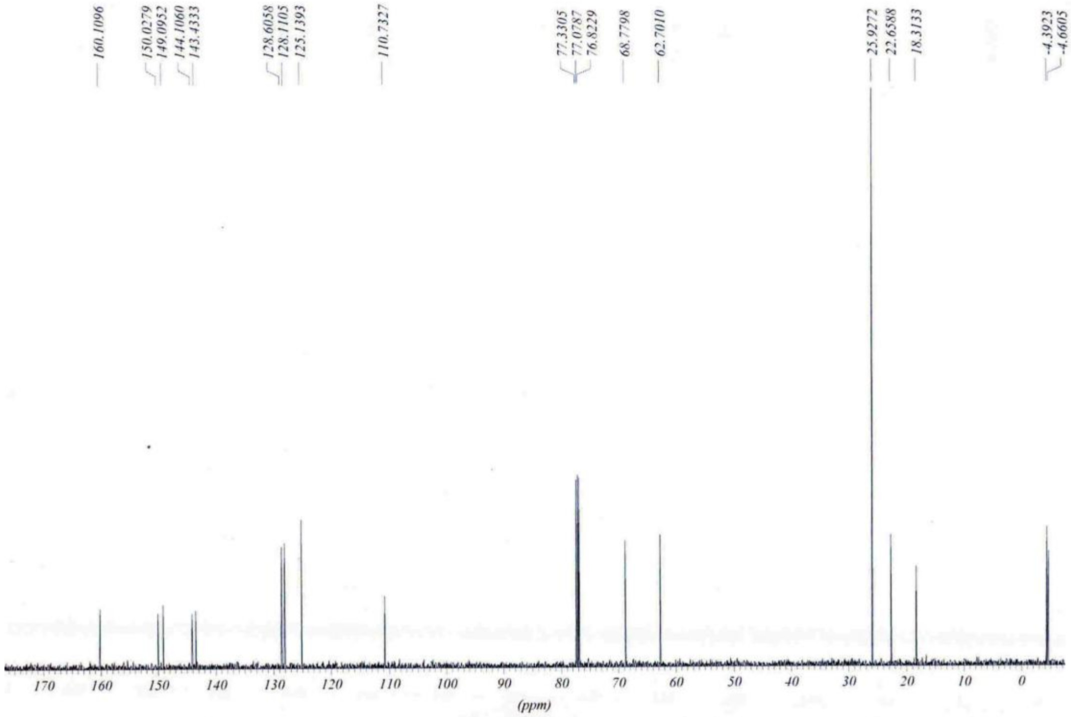

(*S*)-6-[1-(*tert*-Butyldimethylsiloxy)-ethyl]-5-methoxy-[2,2']bipyridinyl-4-yl nonaflate (**72**)

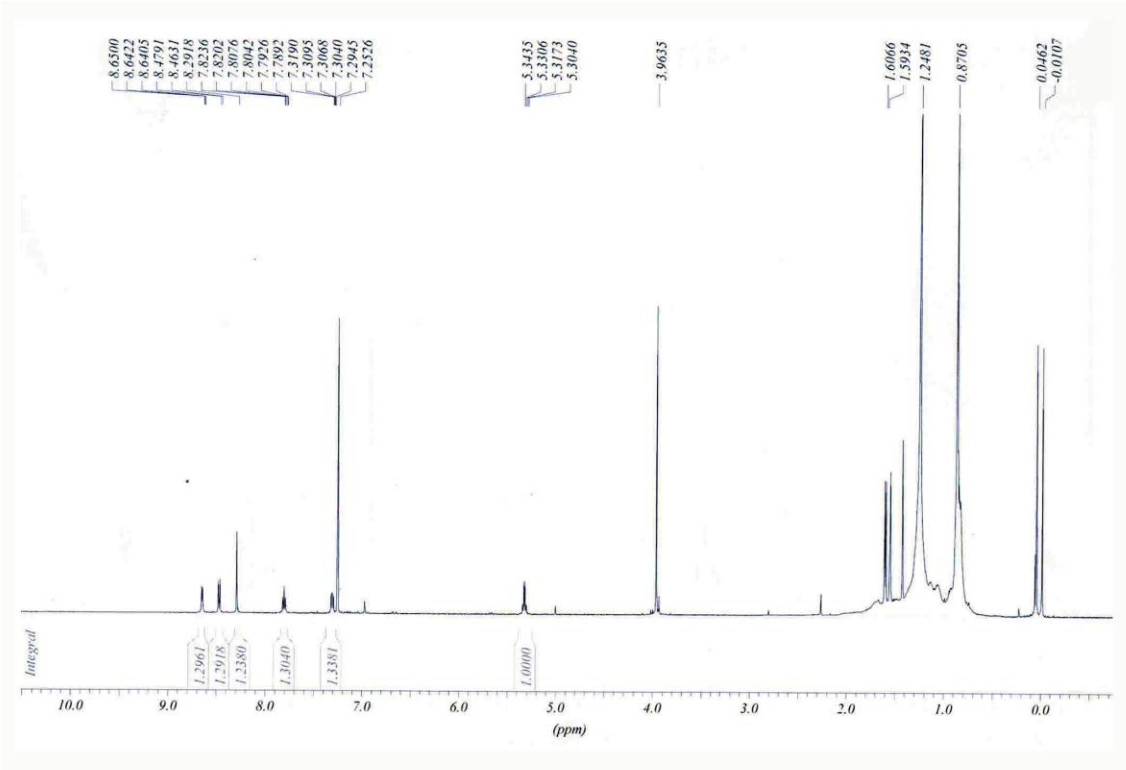

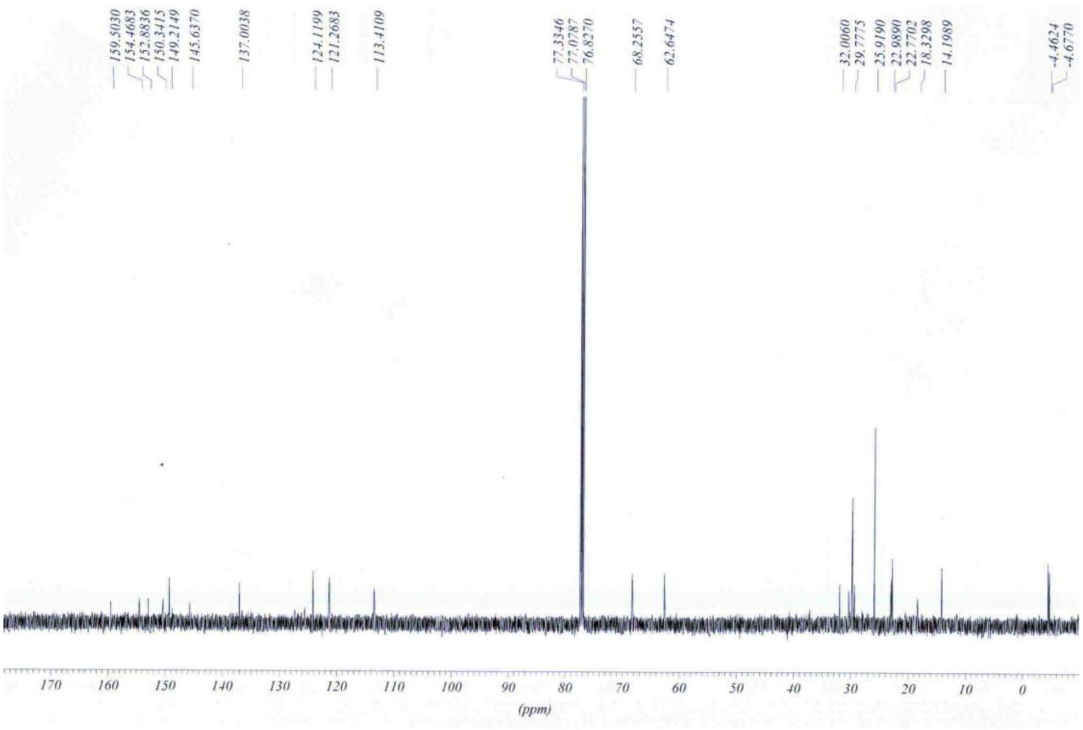

(*S*,*S*)- 2,6-bis-[1-(*tert*-Butyl-dimethyl-silyloxy)-ethyl]-3-methoxy-pyridin-4-yl nonaflate (**73**)

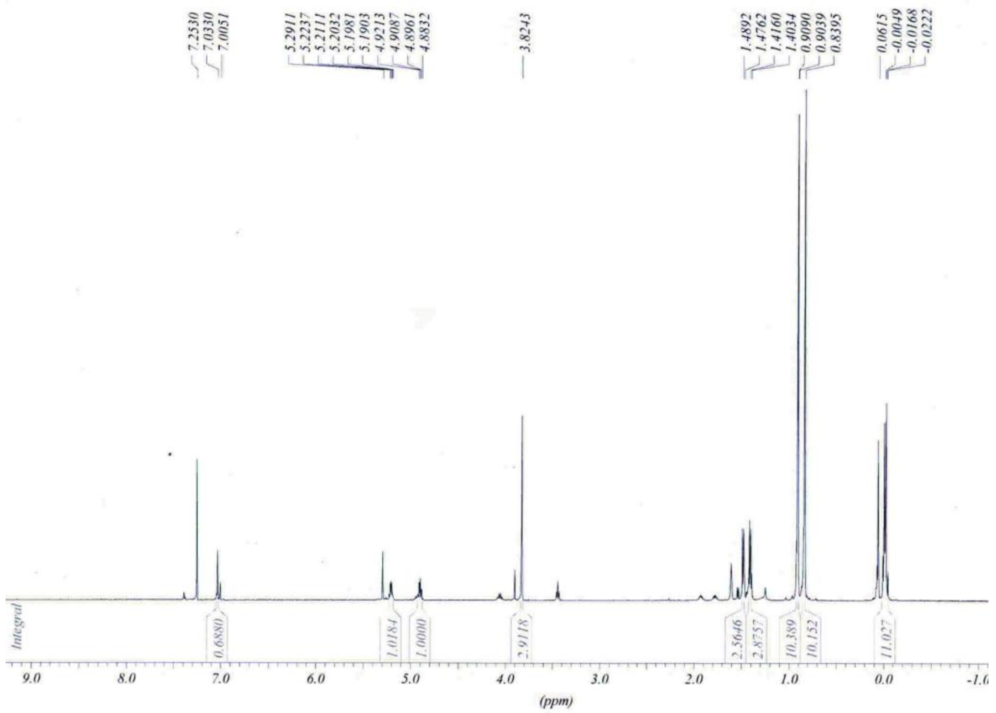

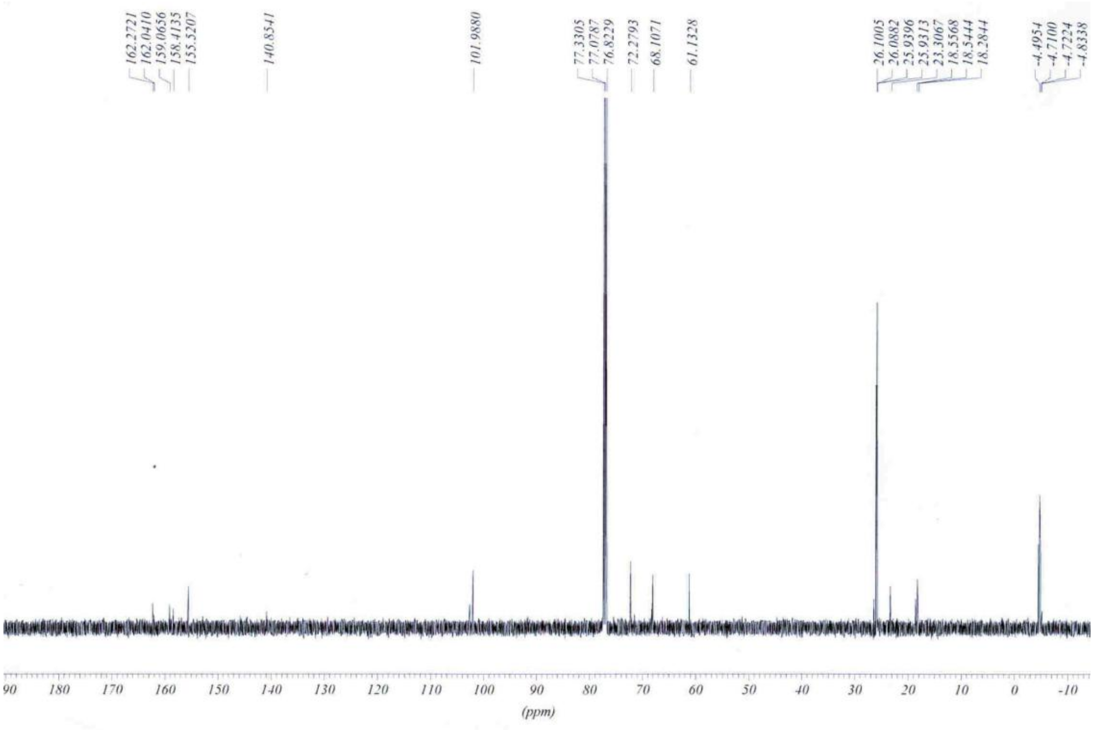

Supplement: File 2 — 1H NMR and 13C NMR spectra of synthesized compounds. [file Beilstein_J_Org_Chem-07-962-s002.pdf]
